# Early postmarketing reporting patterns of resmetirom in FAERS: disproportionality analyses with indication-restricted and active comparators

**DOI:** 10.3389/fphar.2026.1934274

**Published:** 2026-10-29

**Authors:** Xin Zhao, Bi Pan, Tianhao Chen, Jingyi Zhang, Rongxing Zhou

**Affiliations:** 1 Department of General Surgery, Division of Biliary Tract Surgery, West China, Hospital, Sichuan University, Chengdu, Sichuan, China; 2 Department of Ultrasonography, West China Hospital, Sichuan University, Chengdu, China

**Keywords:** disproportionality analysis, FAERS, MASH, MASLD, MedDRA, metabolic dysfunction-associated steatohepatitis, pharmacovigilance, post-marketing safety

## Abstract

**Background:**

Resmetirom is the first approved liver-directed thyroid hormone receptor-beta agonist for noncirrhotic metabolic dysfunction-associated steatohepatitis (MASH) with fibrosis. Early liver-event reporting may be distorted by indication leakage (the treated disease or its manifestations recorded as adverse events), disease progression, and surveillance bias.

**Methods:**

We analyzed FDA Adverse Event Reporting System (FAERS) data from 2024Q1 through 2026Q1. Deduplicated primary- or secondary-suspect reports were assessed at preferred-term, system-organ-class, and nine predefined clinical-cluster levels using frequentist and Bayesian disproportionality metrics. References included all FAERS, US MASH-indication reports, and an exploratory 144-report active comparator. Sensitivity analyses examined cirrhosis indications, serious outcomes, onset-data selection, and co-medications.

**Results:**

Among 3,155,054 deduplicated cases, 1,429 resmetirom reports were analyzed. Versus all non-resmetirom reports, RORs (95% CIs) were 12.13 (10.61–13.88) for hepatocellular injury and 11.46 (9.24–14.21) for cholestasis/biliary events. Gastrointestinal, thyroid/metabolic, and rash/hypersensitivity reporting was also enriched. Hepatic estimates remained elevated after excluding 35 cirrhosis-indication reports and in restricted comparisons, although sparse cells produced wide confidence intervals. The active comparator comprised 142 GLP-1/GIP and two pioglitazone reports. Against this reference, RORs were 1.36 (0.93–1.99) for gastrointestinal intolerance, 0.93 (0.59–1.47) for nausea, 1.42 (0.18–11.18) for cholelithiasis, and 1.95 (1.12–3.41) for diarrhoea. The first three attenuated, whereas diarrhoea remained elevated. Only 206 reports (14.4%) had valid exact onset dates, with systematic differences from reports lacking dates. Sex was missing in 94.6%. Gemfibrozil and clopidogrel were co-reported in three and 25 cases, respectively.

**Conclusion:**

Early FAERS reporting patterns recapitulated recognized gastrointestinal, hepatic, biliary, cutaneous, and thyroid/metabolic domains. Comparator-dependent attenuation suggests that crude all-FAERS estimates partly reflect treatment-context reporting. However, the small GLP-1/GIP-dominant comparator, missing data, target-organ confounding, and spontaneous-report limitations preclude incidence, comparative-risk, or causal inference. These hypothesis-generating patterns support clinical monitoring and denominator-based confirmation.

## Introduction

Metabolic dysfunction-associated steatotic liver disease (MASLD) and its inflammatory fibrotic phenotype, MASH, are major causes of chronic liver disease and cardiometabolic morbidity worldwide (Powell et al., 2021; Fouad et al., 2024; Younossi et al., 2025a; Lee et al., 2024). Contemporary guidelines emphasize fibrosis stage, cardiometabolic risk, non-invasive stratification, and longitudinal monitoring across hepatology, endocrinology, cardiology, and primary care (European Association for the Study of the Liver European Association for the Study of Diabetes and European Association for the Study of Obesity, 2024; Younossi et al., 2025b). Disease progression reflects interacting metabolic stress, hepatocyte injury, inflammation, fibrogenesis, and vascular and immune remodeling (Hagström et al., 2024; Huang et al., 2025; Steinberg et al., 2025; Kumar et al., 2021; Horn and Tacke, 2024); because fibrosis stage is the strongest predictor of liver-related outcomes, fibrosis-enriched eligibility and monitoring frameworks are central to disease-directed therapy (Feng et al., 2025).

The therapeutic field has changed rapidly. Current drug development in MASH targets incretin signaling, thyroid hormone receptor-beta biology, fibroblast growth factor pathways, lipid handling, mitochondrial stress, inflammation, and fibrosis (Do et al., 2025). Resmetirom is a liver-directed, selective thyroid hormone receptor-beta agonist intended to improve intrahepatic lipid metabolism and downstream steatohepatitis biology while limiting systemic thyroid hormone effects (Sinha et al., 2025; Ratziu et al., 2025). Early clinical and translational studies further established the rationale for targeting hepatic thyroid hormone receptor-beta biology in MASH (Li et al., 2023; Kannt et al., 2021). In the phase 3 MAESTRO-NASH trial, resmetirom improved histological endpoints in adults with nonalcoholic steatohepatitis and fibrosis, including MASH resolution and fibrosis improvement, and the broader MAESTRO program established the clinical foundation for the first approved pharmacotherapy in this indication (Harrison et al., 2024a; Harrison et al., 2023; Harrison et al., 2024b).

Approval has shifted the key safety question. The central issue is no longer only whether resmetirom can work under trial conditions, but how clinicians should interpret early safety reports in broad practice. Guidance and expert recommendations have begun to define candidate selection, cirrhosis exclusion, baseline assessment, dose and drug-interaction considerations, and follow-up monitoring (Chen V. L. et al., 2025; Noureddin et al., 2024). Nevertheless, post-marketing exposure differs from trial exposure. Treated patients may have incomplete baseline fibrosis assessment, uncertain or legacy indication coding, diabetes and obesity pharmacotherapy, statin exposure, or potential hepatotoxic co-medications. In addition, the target organ is already abnormal; aminotransferase increases, bilirubin increases, pruritus, gallbladder terms, cirrhosis terminology, and fatigue may represent adverse events, monitoring findings, disease progression, coding heterogeneity, or confounding by the treated condition.

Spontaneous-report pharmacovigilance cannot estimate incidence or prove causality, but it can identify reporting concentrations after a newly approved drug enters practice (Keam, 2024). We therefore analyzed FAERS reports for resmetirom from 2024Q1 through 2026Q1 using PT-, SOC-, and predefined cluster-level analyses. To address the central problem of indication bias, we paired the broad all-FAERS comparison with MASH-indication-restricted and active-comparator analyses, explicitly evaluated cirrhosis-related indication coding, and quantified onset-data selection and co-medication context. The goal was to distinguish known or expected reporting domains from observations that warrant targeted follow-up rather than to designate new causal adverse reactions.

## Methods

### Study design, data source, and reporting window

We conducted a retrospective observational pharmacovigilance study using public FAERS quarterly ASCII files from 2024Q1 through 2026Q1. This period captured the first available postapproval reporting window for resmetirom and the most recent locally available quarter at data lock. FAERS contains spontaneous reports submitted to the US Food and Drug Administration by manufacturers, healthcare professionals, consumers, and other reporters; because exposed-person denominators are unavailable, it supports signal detection rather than incidence estimation (U.S. Food and Drug Administration, 2026a). Analyses were implemented in Python 3.12.13 with NumPy 2.3.5.

The analysis processed the standard quarterly FAERS tables. These included DEMO for case identifiers, demographics, receipt dates, country, reporter occupation, and event dates; DRUG for drug names, drug-sequence numbers, role codes, and therapy start dates; REAC for reaction PTs; and INDI for reported indications linked to drug sequence when available. OUTC captured serious-outcome codes, THER captured therapy dates when present, RPSR captured report-source codes, and DELETE identified cases removed by FDA data maintenance. The study used deidentified public data and did not involve patient contact or identifiable private information.

### Deduplication and exposure definition

Case-level deduplication followed a conservative latest-version strategy. Within the same CASEID, we retained the record with the latest FDA_DT; if multiple records had the same FDA_DT, the highest case version was retained, followed by the highest PRIMARYID as a final tie-breaker. CASEIDs appearing in DELETE files were excluded before analytic case construction. DRUG records were standardized by uppercasing, trimming spaces, and searching normalized drug-name variants for resmetirom, including REZDIFFRA, RESMETIROM, and MGL-3196. The primary target set included cases in which resmetirom was coded as primary suspect or secondary suspect and at least one reaction PT was available. Cases with resmetirom in concomitant, interacting, or other roles were retained only for sensitivity analyses. The primary reference set comprised all non-resmetirom FAERS cases in the same reporting window with at least one reaction PT.

### Event coding, MedDRA SOC mapping, and clinical-cluster construction

Reaction terms were evaluated at three levels. After case-level deduplication, each unique case contributed no more than once to a given PT, primary SOC, or clinical cluster, regardless of duplicate resmetirom DRUG rows or a combined primary-suspect/secondary-suspect role pattern. PTs were mapped to MedDRA version 29.0 primary SOCs using the licensed ASCII dictionary (International Council for Harmonisation of Technical Requirements for Pharmaceuticals for Human Use, 2026). Nine clinical clusters were encoded in the analysis script before calculation of disproportionality outputs: hepatocellular injury, cholestasis/biliary events, gastrointestinal intolerance, thyroid/metabolic events, rash/hypersensitivity, neuropsychiatric events, muscle toxicity, cardiovascular events, and renal events. The clusters were selected for clinical relevance to MASH, resmetirom pharmacology, label-based monitoring, and differential diagnosis. They were not preregistered or independently adjudicated by a blinded external panel. Each PT was assigned to one dictionary cluster only, while one report could enter multiple clusters through different PTs. The full PT list, rationale, development timing, and overlap rule are provided in Supplementary Table S1.

### Disproportionality analysis

For each PT, SOC, or clinical cluster, we constructed a 2 × 2 table in which a was the number of target reports with the event, b target reports without the event, c reference reports with the event, and d reference reports without the event; N = a + b + c + d. For RORs and their log-Wald 95% confidence intervals only, 0.5 was added to all 4 cells if any cell was zero. PRR and Pearson chi-square used uncorrected counts, and Bayesian calculations used uncorrected counts with the stated priors. The disproportionality metrics were calculated as follows:

$$\text{ROR}=\left(a/b\right)/\left(c/d\right)=\text{ad}/\text{bc}$$

$$\text{ROR }95\%\text{ CI}=\exp\left[\ln\left(\text{ROR}\right)\;\pm \;1.96\surd \left(1/a+1/b+1/c+1/d\right)\right]$$

$$\text{PRR}=\left[a/\left(a+b\right)\right]/\left[c/\left(c+d\right)\right]$$

$$X^{2}=N{\left(\text{ad}-\text{bc}\right)}^{2}/\left[\left(a+b\right)\left(c+d\right)\left(a+c\right)\left(b+d\right)\right]$$

$${\gamma =\gamma }_{11}\left(N+\alpha \right)\left(N+\beta \right)/\left[\left(a+b+\alpha_{1}\right)\left(a+c+\beta_{1}\right)\right]$$

$$E\left(\text{IC}\right)=\log_{2}\left\{\left(a+\gamma_{11}\right)\left(N+\alpha \right)\left(N+\beta \right)/\left[\left(a+b+\alpha_{1}\right)\left(a+c+\beta_{1}\right)\left(N+\gamma \right)\right]\right\}$$

$$V\left(\text{IC}\right)={\left(T_{1}+T_{2}+T_{3}\right)/\left(\ln2\right)}^{2}$$

$$T_{1}=\left[N-a+\gamma -\gamma_{11}\right]/\left[\left(a+\gamma_{11}\right)\left(N+\gamma +1\right)\right]$$

$$T_{2}=\left[N-a-b+\alpha -\alpha_{1}\right]/\left[\left(a+b+\alpha_{1}\right)\left(N+\alpha +1\right)\right]$$

$$T_{3}=\left[N-a-c+\beta -\beta_{1}\right]/\left[\left(a+c+\beta_{1}\right)\left(N+\beta +1\right)\right]$$

$${\text{IC}}_{025}=E\left(\text{IC}\right)-2\surd V\left(\text{IC}\right)$$

The Bayesian confidence propagation neural network used α_1_ = β_1_ = 1, α = β = 2, and γ_11_ = 1 (Bate et al., 1998). IC and IC025 denote the posterior moment approximation and its two-standard-deviation lower bound; IC025 is not an exact posterior quantile. These are deterministic calculations, not Monte Carlo estimates. We report three conventional criteria separately: lower ROR confidence bound >1; PRR ≥2 with χ^2^ ≥ 4; and IC025 > 0, with at least three target cases (Evans et al., 2001; Bate et al., 1998; Chen H. et al., 2024).

### Time-to-onset, co-medication context, and sensitivity analyses

Time to onset was event date minus resmetirom therapy-start date when both dates were exact and internally valid. Missing or partial dates, negative intervals, and intervals over 3,650 days were excluded. We report medians and interquartile ranges descriptively. Because only 206 of 1,429 reports (14.4%) had valid intervals, we compared report characteristics according to onset availability. Weibull models were retained only as exploratory supplementary analyses for groups with at least 20 records. Day-0 values, which may reflect same-day events or date granularity, were shifted to 0.5 days solely because the Weibull likelihood requires positive values; a sensitivity analysis excluded day-0 records entirely.

Co-medication categories were defined by exact generic and brand-name lists before event aggregation. The potential-hepatotoxicity screen comprised methotrexate, amiodarone, valproate/valproic acid/divalproex, azathioprine, isoniazid, nitrofurantoin, tamoxifen, methimazole, propylthiouracil, ketoconazole, fluconazole, voriconazole, terbinafine, diclofenac, carbamazepine, phenytoin, allopurinol, minocycline, dantrolene, infliximab, cyclophosphamide, doxorubicin, paclitaxel, cisplatin, imatinib, pembrolizumab, and nivolumab. The CYP2C8-related screen comprised gemfibrozil, clopidogrel, trimethoprim, montelukast, deferasirox, rifampin/rifampicin, carbamazepine, phenytoin, phenobarbital, and efavirenz. This deliberately broad screen combined recognized CYP2C8 interaction terms with exploratory enzyme-modifier terms; not every entry is an FDA-listed CYP2C8 inhibitor or inducer. The FDA drug-interaction table identifies montelukast as a CYP2C8 substrate, while resmetirom-specific recommendations for gemfibrozil and clopidogrel were taken from the current REZDIFFRA prescribing information (DailyMed, 2026; U.S. Food and Drug Administration, 2026b); exact drug-level definitions are provided in Supplementary Table S2. Main disproportionality used all non-resmetirom FAERS reports. Clinical comparators were selected before execution of this comparator analysis to provide an indication-adjacent treatment context; they were not specified in the original protocol or preregistered. GLP-1/GIP therapies are widely used in patients with obesity, type 2 diabetes, and MASH and have a recognized gastrointestinal reporting background, whereas pioglitazone and vitamin E have been used in selected patients with MASH. These agents were not assumed to be pharmacologically interchangeable with resmetirom. Comparisons were restricted to US reports with MASH-related indication coding, first against other indication-restricted reports (target n = 1,017; reference n = 218) and then against active-comparator MASH reports (target n = 1,017; reference n = 144). The active comparator comprised 142 GLP-1/GIP and two pioglitazone reports; vitamin E contributed none. This analysis was exploratory, underpowered, and not a definitive safety benchmark. Because all target cases were US reports, geographic restriction did not create a distinct target population; the clinically restricted comparisons already used US reference reports, and no separate US-only scenario was presented. Restricting the reference alone could still change estimates and is not mathematically equivalent to the broad comparison. Additional analyses restricted target role, excluded selected co-medication groups, excluded cirrhosis indications, and assessed complete age/sex subsets.

Operationally, MASH-related indication coding included MASH/NASH, MASLD/NAFLD, steatohepatitis, nonalcoholic fatty liver disease, fatty liver/hepatic steatosis, and hepatic/liver fibrosis after text normalization. This was a broad coding proxy, not confirmation of biopsy-proven MASH or F2-F3 fibrosis. Eligible target reports required a qualifying indication linked to a resmetirom drug sequence and no resmetirom-linked cirrhosis indication. The indication-restricted reference required a qualifying indication anywhere in INDI, no cirrhosis indication, and no resmetirom in any role. The active reference additionally required a PS/SS-coded GLP-1/GIP agent, pioglitazone, or vitamin E with a qualifying indication linked to that comparator drug sequence. Country restriction used occurrence country, or reporter country when occurrence country was missing. All groups required at least one reaction PT. Differences in indication-linkage requirements and residual diagnostic misclassification may persist despite this restriction.

## Results

### Case selection and reporting characteristics

Across the selected FAERS quarters, 3,634,333 DEMO rows were processed. After latest-version deduplication and DELETE exclusion, 3,155,054 unique cases remained. We identified 1,519 cases with any resmetirom role and 1,429 main-analysis cases in which resmetirom was coded as primary or secondary suspect and at least one reaction PT was available. The reference population comprised 3,153,534 non-resmetirom reports with at least one PT (Figure 1; Supplementary Table S3).

**FIGURE 1:**
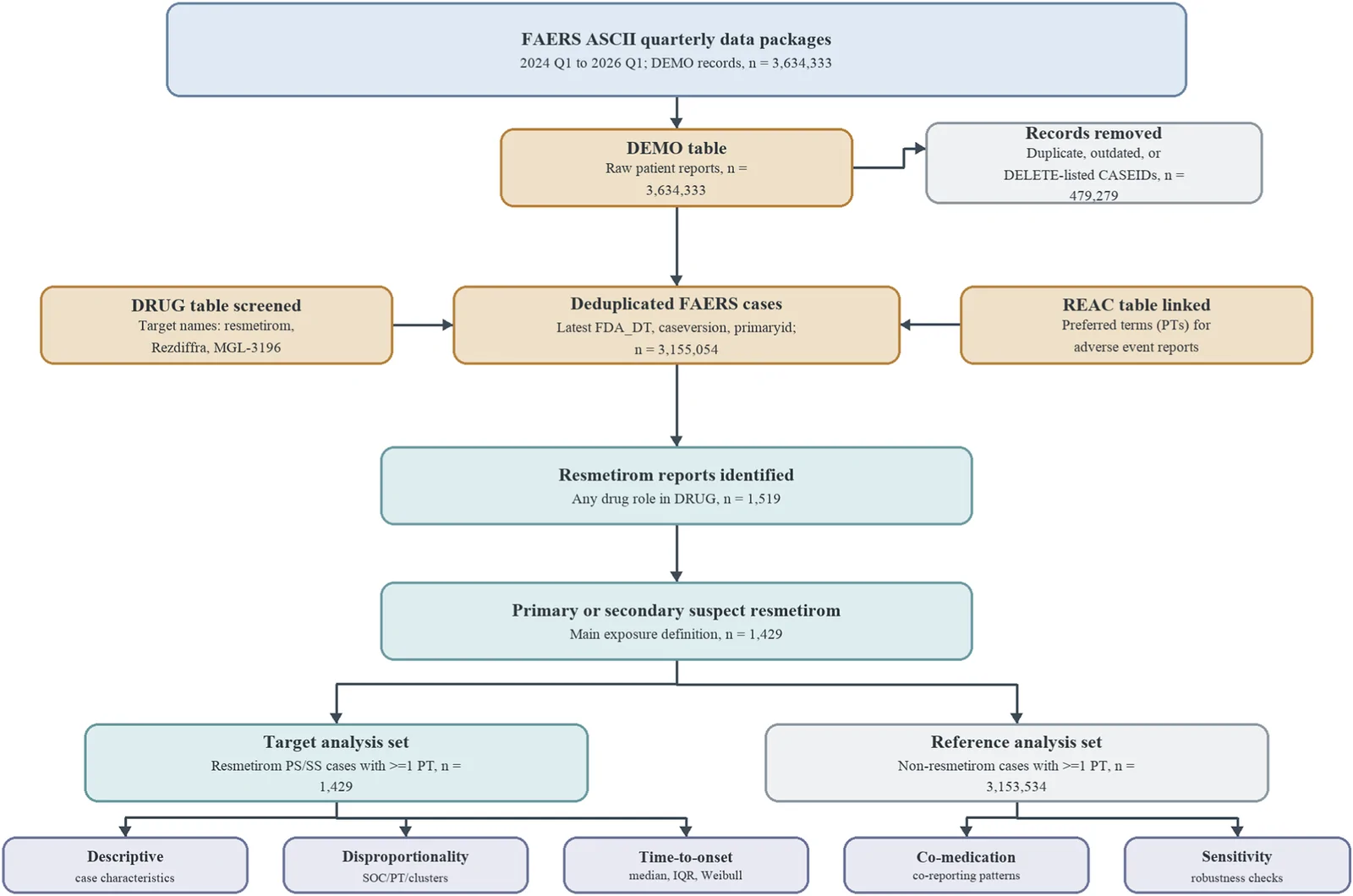
Case selection and analytic workflow for resmetirom reports in FAERS. Quarterly ASCII files from 2024 Q1 through 2026 Q1 contained 3,634,333 DEMO rows. After latest-version case-level deduplication and exclusion of deleted records, 3,155,054 unique FAERS cases remained. Linkage of the DRUG and REAC tables identified 1,519 reports naming resmetirom under any drug role. The main target set comprised 1,429 reports in which resmetirom was coded as primary or secondary suspect and at least one MedDRA Preferred Term was available; 3,153,534 non-resmetirom reports with at least one Preferred Term formed the all-FAERS reference set. The workflow also shows the descriptive, disproportionality, time-to-onset, co-medication, and sensitivity analyses. Counts represent spontaneous reports rather than exposed patients. DEMO, demographic and administrative table; DRUG, drug table; REAC, reaction table; FAERS, FDA Adverse Event Reporting System; MedDRA, Medical Dictionary for Regulatory Activities; PS, primary suspect; SS, secondary suspect.

All target reports were from the United States, a pattern most plausibly related to initial US market availability and the US-centered scope of FAERS; it should not be interpreted as evidence that resmetirom was not used or that adverse events did not occur elsewhere. The sex field was missing in 1,352/1,429 reports (94.6%), with one additional report coded UNK; 76 reports had female/male coding. Age was available in only 163 (11.4%), with median age 63 years (IQR 54–70). Reporter occupation in DEMO was available for 1,272 cases: consumers accounted for 558 (39.0%), other healthcare professionals 415 (29.0%), physicians 271 (19.0%), and pharmacists 28 (2.0%); occupation was unknown for 157 (11.0%). In contrast, a usable RPSR report-source row was absent for 1,358 reports (95.0%). Resmetirom was coded as primary suspect in 1,220 reports, as both primary and secondary suspect in 186, as secondary suspect alone in 22, and as both concomitant and primary suspect in one. At the case level, 426 reports (29.8%) contained any serious-outcome code and 65 (4.5%) contained a death code (Table 1; Supplementary Tables S4-S5; Supplementary Figure S1).

**TABLE 1:** Baseline reporting characteristics of resmetirom FAERS cases.

Characteristic	Category	n	%	Data note
Age	Median (IQR), years	163 available	11.4	63 (54–70); mean 61.4, SD 12.0
Sex	Unknown	1352	94.6	​
Sex	Female	49	3.4	​
Sex	Male	27	1.9	​
Country/region	United States	1429	100.0	​
Reporter occupation	Consumer	558	39.0	DEMO OCCP_COD
Reporter occupation	Other health professional	415	29.0	DEMO OCCP_COD
Reporter occupation	Physician	271	19.0	DEMO OCCP_COD
Reporter occupation	Pharmacist	28	2.0	DEMO OCCP_COD
Reporter occupation	Unknown	157	11.0	DEMO OCCP_COD
RPSR report-source code	No usable RPSR row	1358	95.0	Distinct from reporter occupation
Resmetirom role	Primary suspect only	1220	85.4	​
Resmetirom role	Primary and secondary suspect	186	13.0	​
Resmetirom role	Secondary suspect only	22	1.5	​
Serious outcome	Any serious outcome code	426	29.8	Case-level; categories below are not mutually exclusive
Serious outcome	Death	65	4.5	Unique case-level count
Serious outcome	Hospitalization	88	6.2	Unique case-level count
Serious outcome	Life-threatening	8	0.6	Unique case-level count
Serious outcome	Disability	7	0.5	Unique case-level count
Serious outcome	Required intervention	4	0.3	Unique case-level count
Serious outcome	Other serious	395	27.6	Unique case-level count
Time-to-onset	Valid exact start and event dates	206	14.4	36 of 206 were coded as day 0
Co-medication flag	Statin	267	18.7	Co-reporting context
Co-medication flag	GLP-1/GIP therapy	163	11.4	Co-reporting context
Co-medication flag	Potential hepatotoxic drug	79	5.5	Prespecified screening list
Co-medication flag	CYP2C8-related drug	70	4.9	Prespecified screening list

Percentages use 1,429 main-analysis resmetirom primary/secondary suspect reports with at least one reaction preferred term as the denominator unless otherwise indicated. The sex field was missing in 1,352 reports; one additional UNK-coded record leaves 76 female/male-coded reports. The resmetirom role categories shown exclude one additional C + PS-coded report.

### Temporal reporting and indication structure

Resmetirom reporting rose after market entry, from two reports in 2024Q2 to 327 in 2025Q3, followed by 263 in 2025Q4 and 317 in 2026Q1. This nonmonotonic early-postlaunch pattern is compatible with stimulated reporting and changing exposure but cannot establish a Weber effect without dispensing denominators. Leading indications were MASH (806 reports), unknown indication (306), non-alcoholic steatohepatitis (124), hepatic steatosis (80), hepatic fibrosis (62), and hepatic cirrhosis (35) (Figure 2; Supplementary Tables S6A-S6B).

**FIGURE 2:**
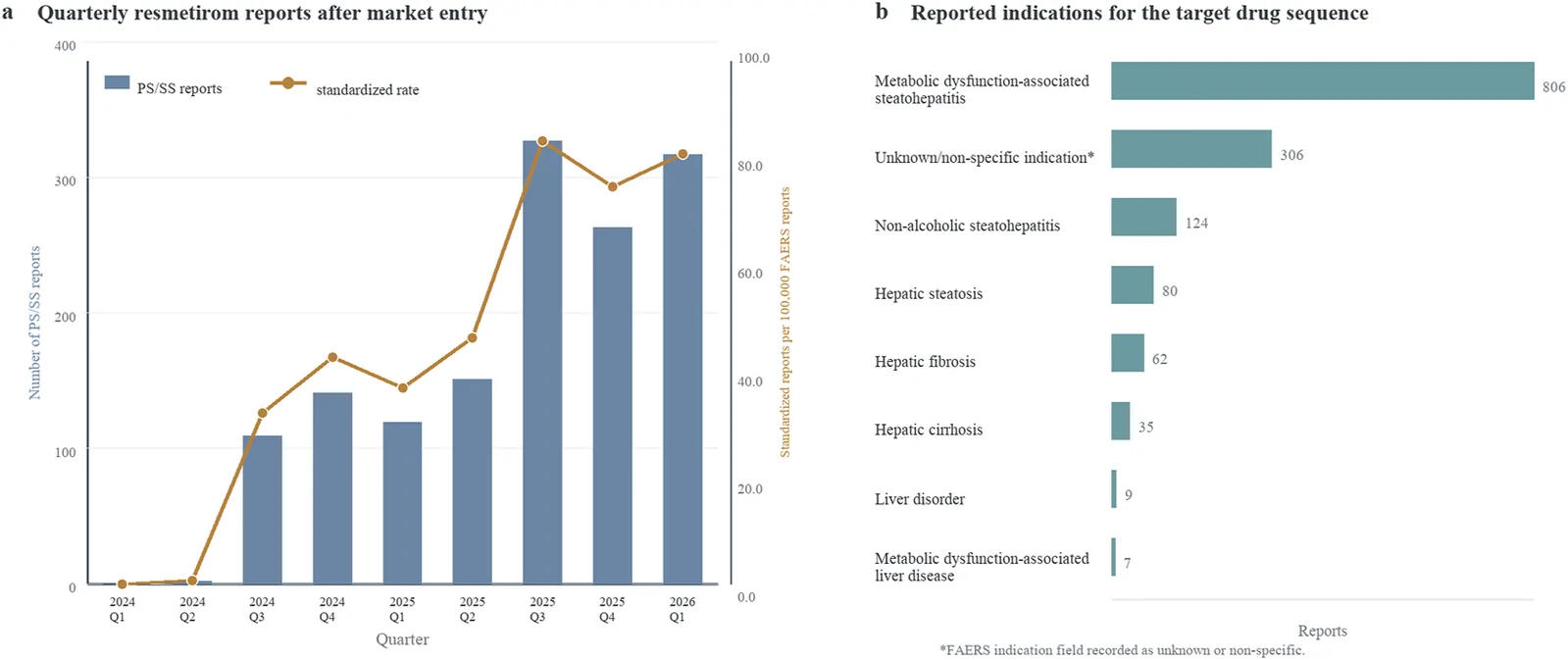
Quarterly reporting and indication structure of resmetirom FAERS cases. **(a)** Blue bars show quarterly counts of resmetirom primary- or secondary-suspect reports with at least one reaction Preferred Term from 2024 Q1 through 2026 Q1 on the left axis; orange points and lines show the corresponding number per 100,000 all-FAERS reports on the right axis. **(b)** Horizontal bars show the eight most frequently recorded indication Preferred Terms linked by INDI_DRUG_SEQ to a resmetirom drug sequence. A report may contribute more than one indication record. Unknown/non-specific indication denotes an absent or non-specific FAERS indication entry. Quarterly counts and standardized reporting rates describe spontaneous-report volume and coding patterns; they do not measure utilization, exposure-adjusted incidence, or temporal risk. FAERS, FDA Adverse Event Reporting System; INDI, indication table; PS, primary suspect; SS, secondary suspect.

### Event frequencies at PT, SOC, and clinical-cluster levels

The event-frequency profile was dominated by gastrointestinal, laboratory, cutaneous, and hepatobiliary terms. The most frequent PTs were diarrhoea (253, 17.7%), nausea (226, 15.8%), pruritus (221, 15.5%), alanine aminotransferase increased (128, 9.0%), aspartate aminotransferase increased (123, 8.6%), fatigue (107, 7.5%), abdominal pain upper (101, 7.1%), dizziness (93, 6.5%), vomiting (89, 6.2%), and weight decreased (85, 5.9%).

At the SOC level, gastrointestinal disorders were most common (548 reports, 38.3%), followed by general disorders and administration site conditions (457, 32.0%), investigations (438, 30.7%), skin and subcutaneous tissue disorders (342, 23.9%), hepatobiliary disorders (258, 18.1%), and nervous system disorders (217, 15.2%). Prespecified clinical clusters were led by gastrointestinal intolerance (486, 34.0%), rash/hypersensitivity (272, 19.0%), hepatocellular injury (261, 18.3%), neuropsychiatric events (171, 12.0%), thyroid/metabolic events (103, 7.2%), and cholestasis/biliary events (89, 6.2%). Representative PTs in the four most frequent clusters included diarrhoea, nausea, vomiting, abdominal pain, constipation, and dyspepsia for gastrointestinal intolerance; pruritus, rash, urticaria, and hypersensitivity for rash/hypersensitivity; ALT/AST or hepatic enzyme increases, drug-induced liver injury/liver injury, hepatitis, and hepatic failure for hepatocellular injury; and dizziness, headache, anxiety, depression, insomnia, confusional state, and suicidal ideation for neuropsychiatric events (Table 2; Figure 3; Supplementary Tables S1 and S7; Supplementary Figure S2).

**TABLE 2:** Leading adverse-event frequencies at PT, SOC, and clinical-cluster levels.

Level	Term	Target cases, n	% Of target reports
Preferred term	Diarrhoea	253	17.7
Preferred term	Nausea	226	15.8
Preferred term	Pruritus	221	15.5
Preferred term	Alanine aminotransferase increased	128	9.0
Preferred term	Aspartate aminotransferase increased	123	8.6
Preferred term	Fatigue	107	7.5
Preferred term	Abdominal pain upper	101	7.1
Preferred term	Dizziness	93	6.5
Preferred term	Vomiting	89	6.2
Preferred term	Weight decreased	85	5.9
Preferred term	Hepatic cirrhosis	71	5.0
Preferred term	Hepatic enzyme increased	64	4.5
Preferred term	Abdominal pain	63	4.4
Preferred term	Therapy interrupted	61	4.3
Preferred term	Constipation	60	4.2
System organ class	Gastrointestinal disorders	548	38.3
System organ class	General disorders and administration site conditions	457	32.0
System organ class	Investigations	438	30.7
System organ class	Skin and subcutaneous tissue disorders	342	23.9
System organ class	Hepatobiliary disorders	258	18.1
System organ class	Nervous system disorders	217	15.2
System organ class	Injury, poisoning and procedural complications	134	9.4
System organ class	Musculoskeletal and connective tissue disorders	86	6.0
System organ class	Psychiatric disorders	77	5.4
System organ class	Surgical and medical procedures	77	5.4
Clinical event cluster	Hepatocellular injury	261	18.3
Clinical event cluster	Cholestasis/biliary events	89	6.2
Clinical event cluster	Gastrointestinal intolerance	486	34.0
Clinical event cluster	Thyroid/metabolic events	103	7.2
Clinical event cluster	Muscle toxicity	34	2.4
Clinical event cluster	Neuropsychiatric events	171	12.0
Clinical event cluster	Cardiovascular events	19	1.3
Clinical event cluster	Renal events	10	0.7
Clinical event cluster	Rash/hypersensitivity	272	19.0

A single report could contribute to multiple PTs, SOCs, or clinical clusters.

**FIGURE 3:**
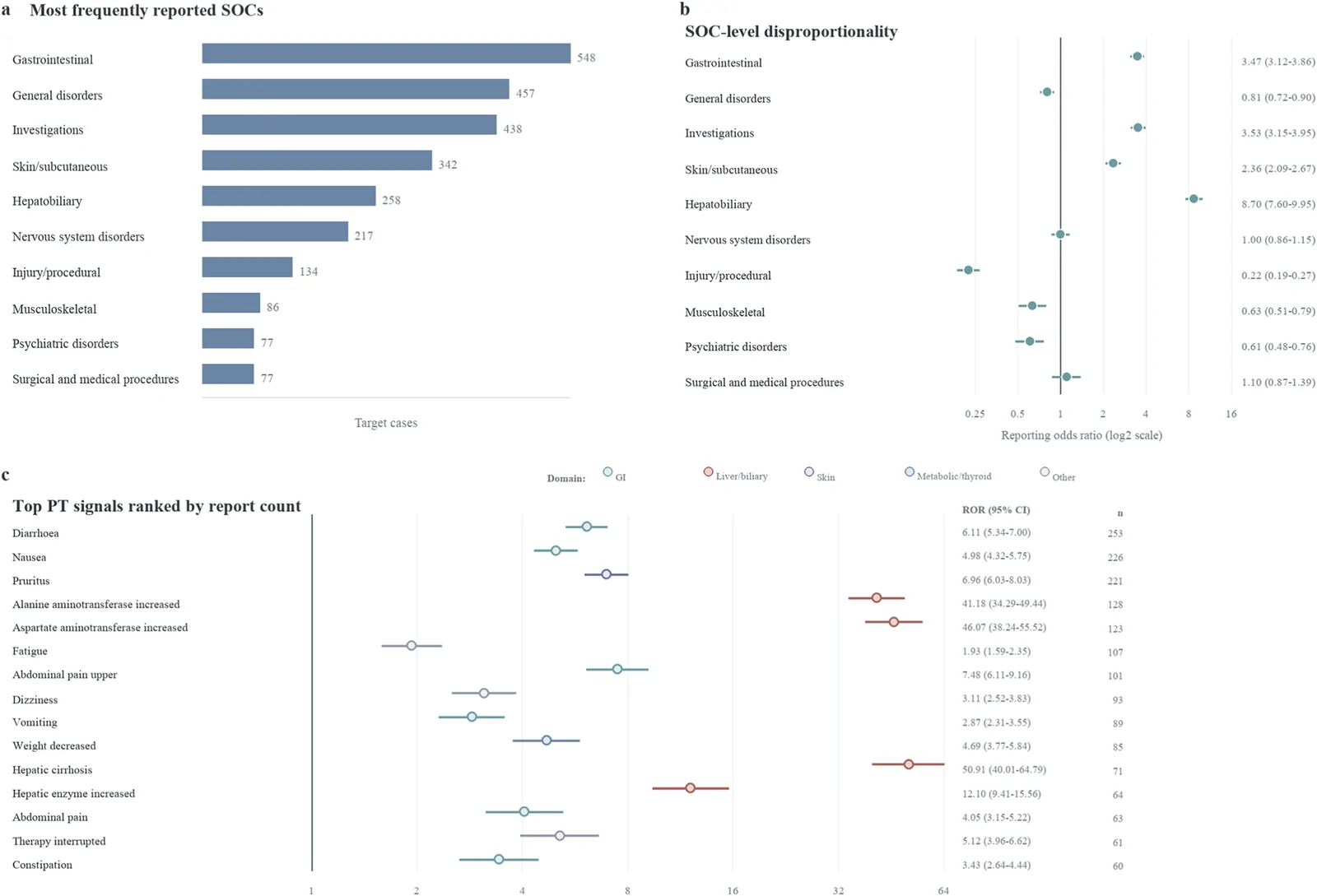
Frequency and disproportionality profile of resmetirom adverse-event reports. **(a)** The 10 most frequently reported MedDRA primary system organ classes (SOCs), ranked by target-case count. **(b)** All-FAERS RORs and 95% CIs for the same SOCs. **(c)** The 15 Preferred Terms (PTs) with the largest target-report counts; points and horizontal lines show RORs and 95% CIs on a base-2 logarithmic axis, colors identify clinical domains, and the right-hand columns provide numerical estimates and target counts. All panels use 1,429 primary- or secondary-suspect target reports with at least one PT and an all-FAERS reference of 3,153,534 reports. After case-level deduplication, a unique case was counted once within each PT or SOC regardless of duplicate resmetirom DRUG records or PS/SS role combinations; one case could contribute to multiple different PTs or SOCs. The vertical reference line denotes ROR = 1. RORs indicate reporting disproportionality and do not estimate incidence or establish causality. CI, confidence interval; FAERS, FDA Adverse Event Reporting System; MedDRA, Medical Dictionary for Regulatory Activities; PS, primary suspect; SS, secondary suspect.

### SOC-, cluster-, and PT-level reporting disproportionality

At the SOC level, reporting was enriched for hepatobiliary disorders (ROR 8.70, 95% CI 7.60–9.95), investigations (ROR 3.53, 95% CI 3.15–3.95), gastrointestinal disorders (ROR 3.47, 95% CI 3.12–3.86), and skin and subcutaneous tissue disorders (ROR 2.36, 95% CI 2.09–2.67) *versus* all non-resmetirom FAERS reports. These broad-reference estimates are reporting associations and are particularly sensitive to target-organ disease and monitoring.

At the predefined-cluster level, hepatocellular injury (261 reports; ROR 12.13, 95% CI 10.61–13.88; IC025 3.09), cholestasis/biliary events (89; ROR 11.46, 95% CI 9.24–14.21; IC025 2.96), gastrointestinal intolerance (486; ROR 4.54, 95% CI 4.07–5.07; IC025 1.58), thyroid/metabolic events (103; ROR 4.46, 95% CI 3.65–5.45; IC025 1.73), and rash/hypersensitivity (272; ROR 3.72, 95% CI 3.26–4.25) met all three prespecified statistical criteria. Neuropsychiatric events met the ROR and IC025 criteria but not the PRR/chi-square criterion. Muscle, cardiovascular, and renal clusters did not meet the ROR criterion (Table 3; Figure 4; Supplementary Tables S8-S11; Supplementary Figure S3).

**TABLE 3:** Key SOC, clinical-cluster, and representative PT reporting disproportionality.

Level and event	Target n (main)	All-FAERS ROR (95% CI)	All-FAERS IC025	US MASH-indication ROR (95% CI)	Active-comparator ROR (95% CI)	Interpretive emphasis
Cluster: hepatocellular injury	261	12.13 (10.61–13.88)†	3.09	9.58 (4.20–21.87)†	38.79 (5.40–278.85)†	Persists, but hepatic surveillance and indication confounding remain
Cluster: cholestasis/biliary	89	11.46 (9.24–14.21)†	2.96	9.22 (2.25–37.80)†	12.21 (1.69–88.43)†	Persists with imprecise matched estimates
Cluster: gastrointestinal intolerance	486	4.54 (4.07–5.07)†	1.58	1.93 (1.37–2.72)†	1.36 (0.93–1.99)	Attenuates to null *versus* active comparator
Cluster: thyroid/metabolic	103	4.46 (3.65–5.45)†	1.73	2.61 (1.19–5.73)†	3.03 (1.09–8.40)†	Positive but imprecise *versus* active comparator
Cluster: rash/hypersensitivity	272	3.72 (3.26–4.25)†	1.48	7.02 (3.25–15.14)†	16.52 (4.06–67.31)†	Positive with wide comparator confidence intervals
Cluster: neuropsychiatric	171	1.75 (1.50–2.06)†	0.49	1.66 (1.00–2.74)	3.64 (1.58–8.41)†	Modest main signal; sparse comparator estimates
PT: AST increased	123	46.07 (38.24–55.52)†	4.69	12.57 (3.08–51.31)†	33.77 (2.09–546.41)†	Monitoring/indication-sensitive hepatic laboratory term
PT: ALT increased	128	41.18 (34.29–49.44)†	4.59	13.10 (3.21–53.45)†	35.19 (2.18–569.30)†	Monitoring/indication-sensitive hepatic laboratory term
PT: blood bilirubin increased	55	41.95 (31.97–55.06)†	4.16	22.81 (1.40–371.15)†	15.08 (0.93–245.85)	Sparse matched-reference cells; very wide interval
PT: hepatic cirrhosis	71	50.91 (40.01–64.79)†	4.50	12.41 (1.71–90.15)†	8.18 (1.12–59.54)†	Cannot distinguish indication leakage, pre-existing disease, or progression
PT: cholelithiasis	11	5.72 (3.16–10.36)†	1.19	1.07 (0.23–4.93)	1.42 (0.18–11.18)	Attenuates to null in both clinical comparators
PT: diarrhoea	253	6.11 (5.34–7.00)†	2.16	2.52 (1.52–4.19)†	1.95 (1.12–3.41)†	Attenuates but remains elevated *versus* active comparator
PT: nausea	226	4.98 (4.32–5.75)†	1.89	1.28 (0.85–1.95)	0.93 (0.59–1.47)	Attenuates to null *versus* active comparator
PT: pruritus	221	6.96 (6.03–8.03)†	2.35	9.91 (3.63–27.04)†	26.50 (3.68–190.81)†	Persists, but active-comparator estimate is sparse
PT: rash	55	1.67 (1.28–2.19)†	0.31	4.19 (1.00–17.51)	5.55 (0.76–40.74)	Comparator intervals include or approach unity

Main target n = 1,429 and all-FAERS, reference n = 3,153,534. US, MASH-indication analyses used target n = 1,017 and reference n = 218. The active-comparator reference contained only 144 reports (142 GLP-1/GIP, two pioglitazone, and 0 vitamin E reports); these estimates are exploratory, underpowered, and not definitive safety benchmarks. IC025 is shown only for the broad main analysis. †95% CI, excludes 1.00; this visual marker does not imply causality or account for multiple comparisons. Restricted-comparator estimates may be unstable when event cells are sparse.

**FIGURE 4:**
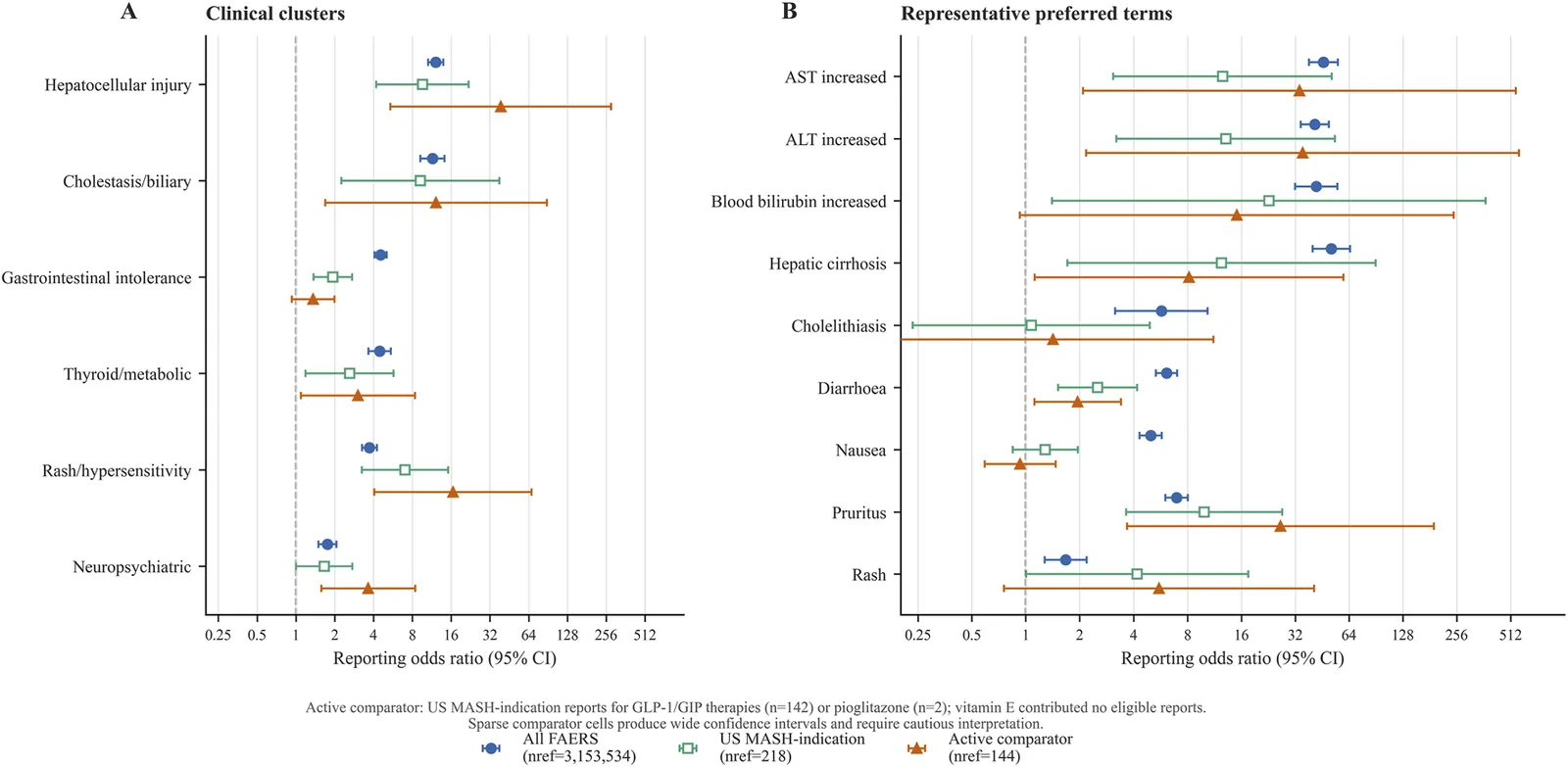
Comparator-dependent disproportionality for clinical clusters and representative Preferred Terms. **(A)** Six prespecified clinical clusters. **(B)** Nine clinically relevant Preferred Terms. Blue filled circles compare 1,429 resmetirom primary- or secondary-suspect reports with 3,153,534 non-resmetirom FAERS reports; green open squares compare 1,017 US resmetirom reports with a MASH indication with 218 non-resmetirom US MASH-indication reports; orange triangles use the same 1,017 target reports and a 144-report active-comparator reference. The active-comparator reference comprised GLP-1/GIP therapies (n = 142) and pioglitazone (n = 2); vitamin E contributed no eligible report. Points denote RORs and horizontal lines 95% CIs on a base-2 logarithmic axis; the dashed vertical line denotes ROR = 1. Wide intervals reflect sparse comparator cells. The active-comparator estimates are exploratory, underpowered, and not definitive safety benchmarks. Disproportionality does not estimate incidence or establish causality. CI, confidence interval; FAERS, FDA Adverse Event Reporting System; GLP-1/GIP, glucagon-like peptide-1/glucose-dependent insulinotropic polypeptide; MASH, metabolic dysfunction-associated steatohepatitis; ROR, reporting odds ratio.

Representative hepatic PTs had large crude estimates versus all FAERS: AST increased (ROR 46.07), ALT increased (ROR 41.18), blood bilirubin increased (ROR 41.95), and hepatic cirrhosis (ROR 50.91). After restricting the reference to US MASH-indication reports, corresponding RORs were 12.57, 13.10, 22.81, and 12.41, respectively, with wide confidence intervals. Excluding the 35 target reports with cirrhosis-related indications changed hepatic cluster estimates minimally (hepatocellular injury ROR 12.10; cholestasis/biliary ROR 11.34). Among these 35 reports, 15 (42.9%) contained any serious-outcome code, 1 (2.9%) contained a death code, and 8 (22.9%) also contained a cirrhosis adverse-event PT. Their leading clinical clusters were gastrointestinal intolerance (9/35, 25.7%), hepatocellular injury (7/35, 20.0%), neuropsychiatric events (6/35, 17.1%), cholestasis/biliary events (3/35, 8.6%), thyroid/metabolic events (2/35, 5.7%), and rash/hypersensitivity (2/35, 5.7%); no muscle, cardiovascular, or renal cluster event was recorded. FAERS cannot adjudicate whether cirrhosis terms represent pre-existing disease, miscoding, off-label use, or disease progression (Supplementary Tables S11, S12).

### Sensitivity analyses

Clinical comparators materially changed several nonhepatic estimates. Against the active-comparator MASH reference, gastrointestinal intolerance attenuated from ROR 4.54 to 1.36 (95% CI 0.93–1.99), nausea from 4.98 to 0.93 (95% CI 0.59–1.47), diarrhoea from 6.11 to 1.95 (95% CI 1.12–3.41), and cholelithiasis from 5.72 to 1.42 (95% CI 0.18–11.18). Hepatocellular and cholestasis/biliary cluster estimates remained elevated, but the active reference had only one corresponding event in each cluster, yielding very wide intervals. Because the 144-report active comparator was predominantly GLP-1/GIP based, these analyses are exploratory and underpowered rather than definitive comparative-safety benchmarks. The three reference analyses are therefore presented together rather than treated as interchangeable confirmations (Table 3; Figure 4; Supplementary Tables S11, S13; Supplementary Figure S4). A domain-level comparison with premarketing and label information is provided in Table 4.

**TABLE 4:** Contextual comparison of premarketing/label safety information with FAERS observations.

Safety domain	Premarketing/label context	FAERS observation	Interpretation
Gastrointestinal	Diarrhoea and nausea are common in MAESTRO-NASH and prescribing information (Harrison et al., 2024a; DailyMed, 2026)	Frequent in FAERS; cluster ROR 4.54 *versus* all FAERS but 1.36 *versus* active comparator	Primarily concordant with known tolerability; nausea lost disproportionality and diarrhoea was attenuated in clinical comparisons
Liver tests/hepatotoxicity	The label warns about hepatotoxicity and recommends liver-test monitoring (DailyMed, 2026)	ALT, AST, and bilirubin PTs had large crude RORs	Hypothesis-generating only; target-organ disease, monitoring, and indication leakage can inflate magnitude
Gallbladder/biliary	Cholelithiasis and cholecystitis are recognized label events (DailyMed, 2026)	Cholestasis/biliary cluster remained elevated; cholelithiasis ROR attenuated to 1.42 *versus* active comparator	Cluster warrants clinical review, but the specific gallstone term was not enriched in matched comparisons
Rash/pruritus	Pruritus and hypersensitivity-type events are represented in postapproval labeling (DailyMed, 2026)	Pruritus was frequent; rash/hypersensitivity cluster remained elevated	Consistent with recognized reporting domains; separate isolated rash from cholestatic pruritus
Thyroid/metabolic	Resmetirom is a selective THR-beta agonist; thyroid monitoring is clinically contextual (Sinha et al., 2025; Ratziu et al., 2025; DailyMed, 2026)	Cluster ROR 4.46 *versus* all FAERS and 3.03 *versus* active comparator	Biologically plausible reporting enrichment, not proof of systemic thyroid toxicity
Cirrhosis	FDA indication is noncirrhotic F2-F3 disease; the label says avoid decompensated cirrhosis and that safety/effectiveness are not established in NASH cirrhosis. AASLD guidance recommends against use in cirrhosis (Chen V. L. et al., 2025; DailyMed, 2026)	35 reports coded a cirrhosis-related indication; 8 also contained a cirrhosis AE PT	Potential off-label or miscoded use and indication leakage materially complicate hepatic interpretation
CYP2C8 interactions	Gemfibrozil coadministration is not recommended; clopidogrel requires resmetirom dose reduction (DailyMed, 2026)	Gemfibrozil was co-reported in 3 cases and clopidogrel in 25	Medication-review reminder only; FAERS cannot confirm concurrent exposure, dose, adherence, or interaction causality

FAERS, observations describe reporting patterns and hypothesis-generating reporting signals, not adverse-event incidence or validated causal effects. Label-based medication recommendations should be applied using current prescribing information and clinical judgment.

### Time-to-onset, co-medication context, and serious-outcome exploration

Valid exact onset data were available for 206/1,429 reports (14.4%). Median observed onset was 14 days (IQR 1–60), including gastrointestinal intolerance at 2 days, rash/hypersensitivity at 6 days, hepatocellular injury at 28 days, and cholestasis/biliary events at 48.5 days (Figure 5; Supplementary Figure S5). Reports with valid onset dates differed from those without valid dates: serious outcomes were more frequent (35.9% vs. 28.8%; OR 1.39, 95% CI 1.02–1.89), age was more often available (16.0% vs. 10.6%; OR 1.60, 95% CI 1.06–2.43), and female/male sex coding was more common (16.5% vs. 3.4%; OR 5.56, 95% CI 3.44–8.98). Of the valid intervals, 36 (17.5%) were day 0. Excluding these shifted the overall median from 14 to 26 days and the biliary Weibull shape from 0.85 to 1.01; both biliary confidence intervals included 1. All onset medians are descriptive summaries of the selected complete-date subset and do not estimate a causal drug-event latency. The exploratory Weibull fits and onset-selection analyses are provided in Supplementary Tables S14, S15A, S15B.

**FIGURE 5:**
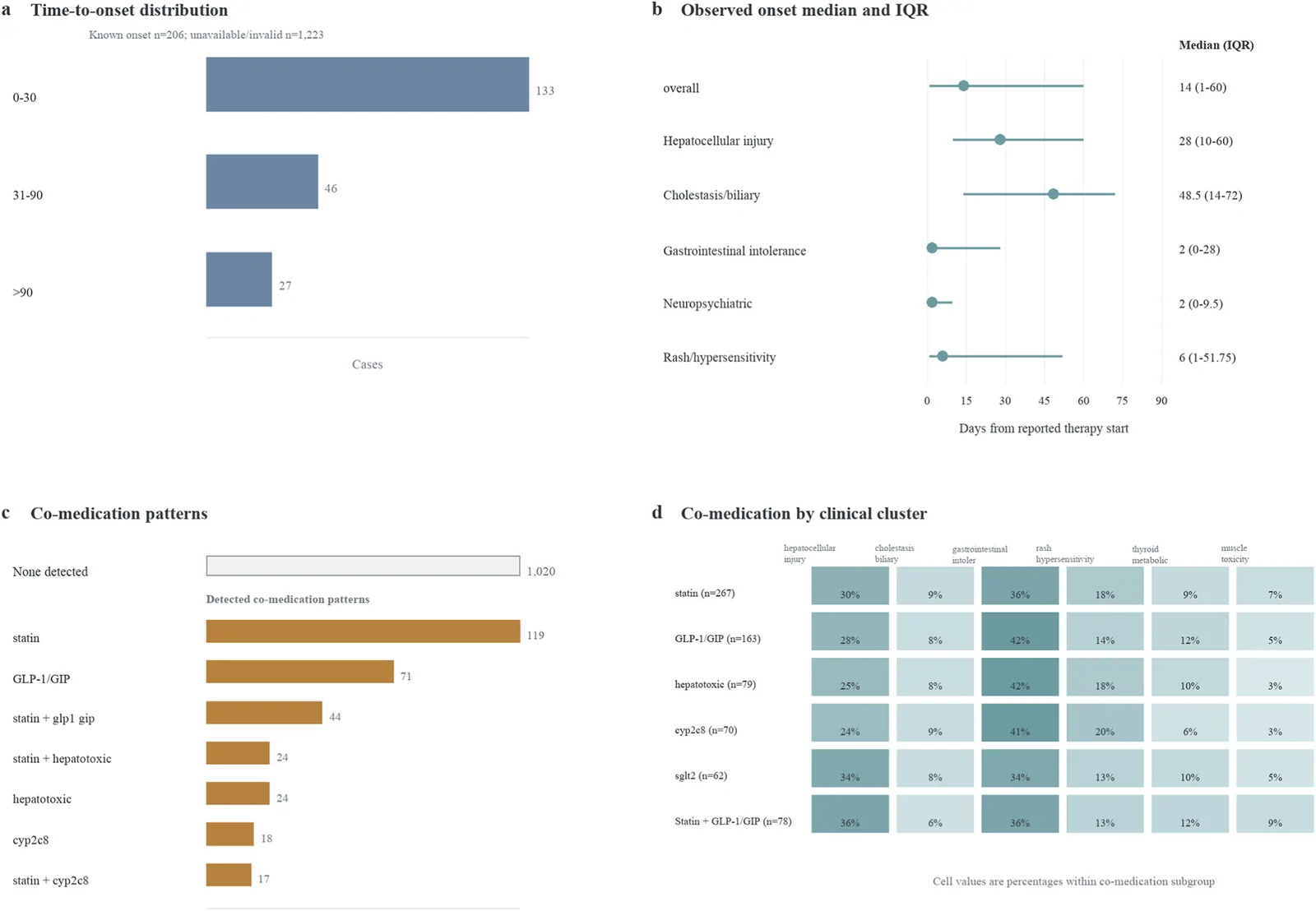
Time-to-onset and co-medication context. **(a)** Distribution of 206 valid exact onset intervals among 1,429 target reports: 0–30 days (n = 133), 31–90 days (n = 46), and >90 days (n = 27); onset was unavailable or invalid in 1,223 reports. **(b)** Median observed onset and interquartile range for the overall target set and clusters with at least 20 valid records: hepatocellular injury (n = 57), cholestasis/biliary events (n = 24), gastrointestinal intolerance (n = 75), neuropsychiatric events (n = 31), and rash/hypersensitivity (n = 40). **(c)** Leading mutually exclusive exact co-medication patterns; no prespecified co-medication was detected in 1,020 reports. **(d)** Percentages of reports containing each clinical cluster within prespecified, overlapping co-medication subgroups; reports may contribute to multiple subgroups and clusters. Because exact onset was available for only 14.4% of target reports, timing summaries are descriptive and vulnerable to selection bias. The exploratory Weibull fits, including the day-0 sensitivity analysis, are reported in Supplementary Tables S14, S15B. Co-reporting does not confirm concomitant exposure, a drug interaction, or causality.

The most frequent prespecified co-medication groups were statins (267 reports), GLP-1/GIP therapies (163), potential hepatotoxic drugs (79), CYP2C8-related drugs (70), and SGLT2 inhibitors (62); complete co-medication-pattern distributions are shown in Supplementary Table S16 and Supplementary Figure S6. Exact potential-hepatotoxic co-reports were led by allopurinol (31), diclofenac (19), ketoconazole (10), and fluconazole (5). CYP2C8-related co-reports were led by montelukast (35), clopidogrel (25), carbamazepine (3), trimethoprim (3), gemfibrozil (3), and phenytoin (2). All gemfibrozil and clopidogrel records were coded as concomitant. One gemfibrozil co-report contained jaundice/ocular icterus and gastrointestinal symptoms, but FAERS cannot confirm concurrent exposure, dose, adherence, temporal sequence, or a causal interaction. These co-reports should therefore be interpreted as a medication-review reminder, not as a confirmed interaction or clinical-risk estimate. Exact drug-level definitions are provided in Supplementary Table S2, and deidentified case summaries in Supplementary Table S17. Exploratory complete-case serious-outcome models are reported in Supplementary Table S18. Case-level seriousness disproportionality was also exploratory: any serious outcome ROR was 0.35 (95% CI 0.32–0.40) and death ROR 0.58 (95% CI 0.45–0.74) versus all FAERS. The lower serious-outcome ROR may reflect enhanced early reporting of mild symptoms and tolerability events for a newly marketed drug, together with a different report composition in the target and all-FAERS groups; it cannot be interpreted as lower hospitalization, mortality, or overall clinical risk (Supplementary Table S19).

## Discussion

### Established safety context and interpretive contribution

Early resmetirom FAERS reports accumulated in domains already recognized from premarketing evidence and prescribing information, including gastrointestinal intolerance, liver-test/hepatobiliary events, pruritus/rash, and thyroid/metabolic terms (Harrison et al., 2024a; DailyMed, 2026; Guirguis et al., 2025). In the 966-patient MAESTRO-NASH trial, diarrhoea occurred in 27.0% and 33.4% of participants receiving 80 and 100 mg, respectively, versus 15.6% with placebo; nausea occurred in 22.0% and 18.9% versus 12.5%, and serious adverse events occurred in 10.9% and 12.7% versus 11.5% (Harrison et al., 2024a). The current label also reports early gastrointestinal symptoms and emphasizes hepatotoxicity monitoring, gallbladder events, hypersensitivity, and CYP2C8-related precautions. The potentially novel contribution is therefore not identification of a new causal reaction. It is the demonstration that apparent enrichment changes with the reference population, together with documentation of cirrhosis-indication coding and incomplete onset data in early practice. Broad all-FAERS disproportionality was pronounced, whereas clinically contextual comparisons attenuated several gastrointestinal and biliary PTs. Evidence syntheses and trial-based quality-of-life analyses place the main tolerability domains within the known premarketing context (Suvarna et al., 2024; Dutta et al., 2024; Younossi et al., 2025c). FAERS report proportions cannot be compared numerically with these trial incidences because their denominators, ascertainment, follow-up, and coding differ. These observations support pharmacovigilance prioritization, medication review, and targeted hypothesis generation rather than regulatory or clinical-risk conclusions.

### Hepatic and cirrhosis-related reporting patterns

The hepatic findings require the greatest restraint. ALT, AST, bilirubin, hepatic cirrhosis, and the hepatocellular and cholestasis/biliary clusters had large crude RORs. However, resmetirom is prescribed precisely in a population with abnormal liver tests, fibrosis surveillance, fluctuating disease activity, and intensive laboratory follow-up. FAERS provides neither patient-specific baseline ALT, AST, or bilirubin values nor serial laboratory trajectories, so a reported increase cannot be separated reliably from a pre-existing abnormality, natural fluctuation, disease progression, or intensified monitoring. This creates confounding by indication, indication leakage, channeling, and surveillance bias. The reduction in several PT estimates after MASH restriction is consistent with these mechanisms. For context, the label’s F2-F3 trial population had ALT elevations above 3 times the upper limit of normal in 11% and 13% of the 80- and 100-mg groups versus 10% with placebo, and AST elevations above 3 times the upper limit in 9% and 12% versus 10%; mean ALT and AST increases were less than 1.5 times baseline at week four and returned toward baseline by week 8 (DailyMed, 2026). These trial observations provide clinical context but cannot validate or refute individual FAERS reports. Persistence of cluster-level associations after cirrhosis-indication exclusion and in active-comparator analyses supports clinical review, but sparse reference cells and wide intervals preclude interpretation as quantified hepatotoxic risk. Liver-test abnormalities should be evaluated against each patient’s pretreatment baseline and longitudinal change rather than against FAERS ROR magnitude alone (The Lancet Gastroenterology and Hepatology, 2024; Petta et al., 2024).

Cirrhosis coding illustrates the problem directly. Thirty-five target reports listed a cirrhosis-related indication despite approval for noncirrhotic MASH with F2-F3 fibrosis; eight of these also listed a cirrhosis adverse-event PT. Such records may reflect off-label use, delayed recognition of advanced disease, legacy terminology, coding errors, or disease progression. They cannot be classified automatically as incident drug-induced cirrhosis. The FDA label says to avoid decompensated cirrhosis and notes that safety and effectiveness have not been established in NASH cirrhosis, whereas AASLD practice guidance recommends against resmetirom use in patients with cirrhosis (Chen V. L. et al., 2025; DailyMed, 2026). Real-world treatment eligibility and cirrhosis staging are themselves uncertain (Le et al., 2025; Noureddin et al., 2025a), and progression to cirrhosis can arise from the underlying disease trajectory (Newsome et al., 2026; Tilg et al., 2026). Cirrhosis-indication coding is therefore interpreted as a medication-use observation requiring verification of treatment eligibility and disease stage.

### Comparator-dependent gastrointestinal and biliary patterns

The gastrointestinal results show why an active comparator matters. Diarrhoea and nausea are common in MAESTRO-NASH and are recognized in prescribing information (Harrison et al., 2024a; DailyMed, 2026). Versus all FAERS, both PTs were strongly enriched, but nausea and the overall gastrointestinal cluster attenuated to null versus MASH active comparators; diarrhoea remained only modestly elevated. Because 142 of 144 active-comparator reports involved GLP-1/GIP therapies, which themselves have substantial gastrointestinal symptom reporting (Sanyal et al., 2025; Newsome et al., 2021), attenuation may reflect a gastrointestinal reporting background shared across treatments used in metabolically complex patients with MASH. Trial and secondary-analysis data support this overlapping treatment context (Noureddin et al., 2025b; Harrison et al., 2025), as do evidence syntheses and reviews of broader GLP-1-based MASH therapy (Wang Y. et al., 2025; Singh et al., 2024). Attenuation does not demonstrate that resmetirom lacks gastrointestinal risk. The analysis is exploratory and underpowered; it supports a prominent and clinically relevant tolerability burden while showing that the crude all-FAERS ROR magnitude is not a definitive comparative-risk estimate.

The biliary profile is similarly mixed. The label reports gallbladder events more often with resmetirom than placebo, although exposure-adjusted rates were below one per 100 person-years in each arm (DailyMed, 2026). Cholelithiasis did not meet the ROR criterion in either restricted comparison, while the broader cholestasis/biliary cluster remained enriched. Its active-comparator interval was very wide, and the cluster also included bilirubin increases, jaundice, and cholestasis rather than gallstones alone. This divergence cannot establish a new gallstone risk or negate the labeled warning. It favors PT-specific clinical review and symptom-triggered evaluation of jaundice, right upper-quadrant pain, or fever instead of treating the aggregate cluster as a single causal syndrome.

Pruritus/rash and thyroid/metabolic terms also require separation of established effects from unresolved observations. Rash and urticaria already appear in prescribing information. The label also describes mean free-T4 decreases of 13% and 17% at 12 months with 80 and 100 mg, versus 2% with placebo, with little T3/TSH change and no associated clinical findings (DailyMed, 2026). Thus, an enriched thyroid/metabolic cluster is not itself evidence of clinical thyroid toxicity. Pruritus has competing explanations including cholestasis, hypersensitivity, and nonspecific symptom reporting. Sparse active-comparator estimates do not resolve these mechanisms. Baseline thyroid status, dose, concomitant medicines, and longitudinal testing are needed to interpret individual reports in the context of liver-directed THR-beta pharmacology (Sinha et al., 2025; Ratziu et al., 2025). Neuropsychiatric enrichment likewise remains an unadjudicated observation requiring PT-level follow-up, not designation of a new adverse reaction.

### Medication and postlaunch reporting context

Medication context provides a practical medication-review reminder. Gemfibrozil, a strong CYP2C8 inhibitor whose coadministration with resmetirom is not recommended, appeared in three reports; clopidogrel, for which the label recommends resmetirom dose reduction, appeared in 25 (DailyMed, 2026; U.S. Food and Drug Administration, 2026b). These records do not prove concurrent exposure or an interaction because FAERS lacks reliable confirmation of dose, adherence, plasma exposure, and temporal sequence. They should prompt careful medication reconciliation at initiation and follow-up, not be presented as a confirmed interaction or clinical-risk estimate. The broader potential-hepatotoxic and CYP2C8 lists are fully disclosed so that readers can distinguish label-based interaction pairs from exploratory screening terms. Statins are also common in patients with steatotic liver disease and remain an important background medication class during reconciliation (Zhou X. D. et al., 2024; Athyros et al., 2020).

The reporting trajectory peaked in 2025Q3, declined in 2025Q4, and rose again in 2026Q1. This pattern can be influenced by increasing uptake, manufacturer reporting, publicity, and stimulated reporting after launch. Although early peaks are often discussed as the Weber effect, FAERS report counts without dispensing denominators cannot establish that phenomenon. Consumer reporters comprised 39.0% by reporter occupation, which may increase sensitivity to symptomatic tolerability events. Published GLP-1 pharmacovigilance analyses further illustrate how gastrointestinal and psychiatric patterns vary with coding and reference choices (Shu et al., 2022; Zhou J. et al., 2024; Chen W. et al., 2024), with similar constraints for neurological and other reported events (Chen H. et al., 2025; Wang M. et al., 2025; Procacci et al., 2025). The lower serious-outcome ROR is consistent with a report mix enriched for mild symptoms during early market adoption and should not be interpreted as lower clinical severity or mortality risk. These features further separate reporting volume and composition from incidence.

### Limitations

#### Missingness and time-to-onset selection

Missingness is a major limitation rather than a routine footnote. Sex was missing in 94.6% of reports, with one additional UNK-coded record; age was available in 11.4%, usable RPSR source coding was absent in 95.0%, and exact onset was available in 14.4%. Onset-available reports were selectively enriched for complete demographics, serious outcomes, and hepatic/biliary clusters. The 24-record biliary Weibull model was unstable, and excluding two day-0 records moved its shape estimate from 0.85 to 1.01, with both confidence intervals crossing 1. Consequently, all onset medians are descriptive summaries of a selected subset and cannot be interpreted as causal drug-event latency.

#### Spontaneous-report and comparator constraints

FAERS is affected by underreporting, duplicate or incomplete information, lack of exposure denominators, absent baseline and longitudinal laboratory data, no case adjudication, residual confounding, and differential reporting. The broad indication-code proxy does not establish MASH histology or fibrosis stage, and the investigator-defined clinical clusters are not independently validated syndromes or standardized MedDRA queries. The all-US target pattern limits geographic generalizability and primarily reflects early US marketing and the scope of FAERS. The active-comparator analysis included only 144 reports, 142 involving GLP-1/GIP therapies, with sparse event cells and wide confidence intervals. Similar interpretive constraints apply across contemporary FAERS disproportionality studies in other therapeutic areas (Li D. et al., 2024; Chen Y. et al., 2025), as illustrated by additional comparative analyses (Yin et al., 2022; Xu et al., 2025). The active comparator is therefore exploratory, underpowered, and not a definitive safety benchmark. Accordingly, the estimates represent reporting patterns or hypothesis-generating reporting signals rather than validated drug-event risks.

#### Clinical implications and future research

Taken together, the analysis supports focused monitoring rather than alarm. Baseline cirrhosis assessment, patient-specific liver-test trends, biliary symptoms, gastrointestinal counseling, pruritus/rash evaluation, thyroid context, and review of CYP2C8 modifiers are reasonable clinical priorities (Chen V. L. et al., 2025; Noureddin et al., 2024; DailyMed, 2026). The findings do not identify incidence, comparative safety, or a definitive new adverse reaction. Confirmation requires denominator-based cohorts with verified exposure, baseline fibrosis and cirrhosis status, longitudinal laboratory values, dose and adherence data, and adequately powered active comparators. Recent AASLD and global recommendations and therapeutic reviews reinforce the need for risk-stratified, longitudinal assessment as the treatment landscape evolves (Bansal et al., 2026; Younossi et al., 2026; Rinella and Sookoian, 2026).

## Conclusion

Early resmetirom FAERS reporting patterns largely recapitulated recognized gastrointestinal, hepatic, biliary, cutaneous, and thyroid/metabolic domains. Clinically contextual comparators attenuated gastrointestinal intolerance, nausea, and cholelithiasis, while hepatic reporting patterns remained elevated but imprecise and highly vulnerable to target-organ confounding and surveillance bias. Cirrhosis-indication coding and co-reported CYP2C8 modifiers are medication-review reminders. These hypothesis-generating reporting signals should guide monitoring and denominator-based follow-up rather than causal, incidence, or comparative-risk claims.

## Data Availability

The original contributions presented in the study are included in the article/Supplementary Material, further inquiries can be directed to the corresponding author.
