# Supplementary material for: Early postmarketing reporting patterns of resmetirom in FAERS: disproportionality analyses with indication-restricted and active comparators
Source: Front Pharmacol. 2026 Oct 29;17:1934274. doi: 10.3389/fphar.2026.1934274 (PMC13754820; doi:10.3389/fphar.2026.1934274)
Supplement: Supplementary file 1 [file Table1.docx]

**Supplementary Material**

# Supplementary Material Contents

| **Order** | **Item** | **Content** |
| --- | --- | --- |
| 1 | Supplementary Figure S1 | Reporting characteristics and data completeness |
| 2 | Supplementary Figure S2 | Extended adverse-event frequency profiles |
| 3 | Supplementary Figure S3 | Extended disproportionality profiles |
| 4 | Supplementary Figure S4 | Sensitivity analyses of cluster- and SOC-level reporting odds ratios |
| 5 | Supplementary Figure S5 | Time-to-onset data availability and observed timing |
| 6 | Supplementary Figure S6 | Co-medication context and exploratory serious-outcome associations |
| 1 | Supplementary Table S1 | Full PT-to-clinical-cluster dictionary, rationale, development timing, and overlap rule. |
| 2 | Supplementary Table S2 | Exact prespecified co-medications, FAERS role codes, and CYP2C8 source framework. |
| 3 | Supplementary Table S3 | Case selection and data processing flow. |
| 4 | Supplementary Table S4 | Complete characteristics of the main analysis set. |
| 5 | Supplementary Table S5 | Report-source and target-drug name audit table. |
| 6 | Supplementary Table S6A | Quarterly reporting trend. |
| 7 | Supplementary Table S6B | Reported indications for resmetirom-associated reports. |
| 8 | Supplementary Table S7 | Complete event-frequency table by PT, SOC, and predefined clinical cluster. |
| 9 | Supplementary Table S8 | Complete SOC-level disproportionality results. |
| 10 | Supplementary Table S9 | PT-level disproportionality results for PTs with at least three target reports. |
| 11 | Supplementary Table S10 | Complete clinical-cluster disproportionality results. |
| 12 | Supplementary Table S11 | Cluster sensitivity analyses, including the exploratory 144-report active comparator. |
| 13 | Supplementary Table S12 | Cirrhosis-indication subgroup and cirrhosis-PT context. |
| 14 | Supplementary Table S13 | Sensitivity analyses for SOC-level signals. |
| 15 | Supplementary Table S14 | Descriptive time-to-onset summary and exploratory Weibull output. |
| 16 | Supplementary Table S15A | Selection analysis for valid exact time-to-onset data. |
| 17 | Supplementary Table S15B | Zero-day sensitivity analyses for time-to-onset and Weibull fitting. |
| 18 | Supplementary Table S16 | Co-medication patterns and co-medication-by-cluster distributions. |
| 19 | Supplementary Table S17 | Deidentified gemfibrozil and clopidogrel co-report summaries. |
| 20 | Supplementary Table S18 | Exploratory serious-outcome analysis. |
| 21 | Supplementary Table S19 | Case-level serious-outcome disproportionality. |

**Supplementary Figure S1. Reporting characteristics and data completeness.** (A) Availability of age and sex information among the 1,429 resmetirom primary- or secondary-suspect reports with at least one MedDRA preferred term (PT) in the US Food and Drug Administration Adverse Event Reporting System (FAERS). Age was available for 163 reports and female/male sex was recorded in 76 reports; 1,352 sex fields were missing and one additional record was coded UNK. (B) Reporter occupation as a percentage of the 1,429 target reports. (C) FAERS drug-role coding for resmetirom and detected target-drug name variants in DRUG records. PS, SS, I, and C denote primary suspect, secondary suspect, interacting, and concomitant roles, respectively; drug-name counts are DRUG-row counts and may exceed report counts. For analytic outcomes, each deduplicated case contributed no more than once to a given PT, system organ class (SOC), or clinical cluster regardless of repeated resmetirom DRUG rows or combined PS/SS coding. (D) Presence of any serious-outcome code and available report-source coding; "unknown/no RPSR row" indicates that no usable report-source row was identified. (E) Quarterly composition of the 1,429 target reports with PTs. Percentages are report based. Missing demographic or source fields reflect spontaneous-report data completeness and should not be interpreted as clinical absence, population incidence, or causal evidence.

**Supplementary Figure S2. Extended adverse-event frequency profiles.** (A) The 30 most frequently reported MedDRA preferred terms (PTs), ranked by the number of target reports containing each PT. (B) Frequencies of the nine prespecified clinical event clusters, expressed as percentages of the 1,429 resmetirom primary- or secondary-suspect reports with at least one PT. (C) Frequencies of 16 MedDRA system organ classes (SOCs) beyond those emphasized in the main figure, using the same denominator. After case-level deduplication, each unique case was counted once within a given PT, SOC, or clinical cluster, regardless of duplicate resmetirom DRUG rows or a combined primary-suspect/secondary-suspect role pattern. A case could contribute to multiple different PTs, SOCs, or clusters; percentages across rows are consequently not expected to sum to 100%. Clinical clusters aggregate predefined, clinically related PTs and complement rather than replace PT- and SOC-level review. Counts describe reporting frequency within this selected spontaneous-report set and do not estimate adverse-event incidence, comparative risk, or causality.

**Supplementary Figure S3. Extended disproportionality profiles.** (A) Reporting odds ratios (RORs) for 23 frequently reported MedDRA preferred terms (PTs). Points show ROR estimates, horizontal lines show 95% confidence intervals (CIs), the right-hand column gives the number of target reports containing the PT, and point colors indicate broad clinical domains. (B) ROR estimates for 16 MedDRA system organ classes (SOCs). (C) ROR estimates for the nine prespecified clinical event clusters, ordered by effect size. The main comparison included 1,429 resmetirom primary- or secondary-suspect reports and 3,153,534 non-resmetirom FAERS reports with at least one PT. Each unique case was counted once within each evaluated PT, SOC, or cluster regardless of duplicate resmetirom DRUG rows or PS/SS role combinations. All forest plots use a base-2 logarithmic ROR axis; the vertical line at ROR=1 denotes equal reporting odds in target and reference reports. Estimates with wide CIs should be interpreted cautiously, particularly for sparse PTs. Disproportionality identifies relative reporting enrichment and does not establish event incidence, excess clinical risk, drug attribution, or causality.

**Supplementary Figure S4. Cluster-level sensitivity analyses across case definitions and comparators.** Cell values are reporting odds ratios (RORs) for nine predefined clinical clusters. Columns show the all-FAERS main comparison, exclusion of target reports with cirrhosis-related indications, a US MASH-indication-restricted comparison, a US MASH active-comparator comparison, primary-suspect-only reports, and exclusions of GLP-1/GIP, statin, or potential-hepatotoxic co-reports. The active comparator contained 144 reports: 142 involving GLP-1/GIP therapies and 2 involving pioglitazone; vitamin E contributed no eligible report. These classes were selected before execution of this comparator analysis to provide an indication-adjacent treatment context; they were not specified in the original protocol or preregistered and were not assumed to be pharmacologically interchangeable with resmetirom. The active-comparator estimates are exploratory, underpowered, and not definitive safety benchmarks because event cells are sparse and confidence intervals are wide. Attenuation of gastrointestinal RORs may reflect the gastrointestinal reporting background of GLP-1/GIP therapies and does not demonstrate that resmetirom lacks gastrointestinal risk. Confidence intervals are reported in Supplementary Table S11. Because all target reports were from the United States, geographic restriction did not create a distinct target population; a separate US-only scenario was therefore not reported. RORs describe reporting disproportionality, not incidence or causality.

**Supplementary Figure S5. Time-to-onset availability and observed timing.** (A) Availability among 1,429 target reports; only 206 (14.4%) had internally valid exact therapy-start and event dates. (B) Distribution of the 206 valid intervals. (C) Median and interquartile range overall and by predefined clinical cluster. All medians are descriptive summaries of this selected complete-date subset and must not be interpreted as causal drug-event latency. Thirty-six valid intervals were coded as day 0. Day-0 values were retained in descriptive summaries, shifted to 0.5 days only for exploratory Weibull likelihood fitting, and excluded in a dedicated sensitivity analysis. Onset-available reports differed from onset-unavailable reports in demographic completeness, serious outcomes, quarter, and hepatic/biliary cluster prevalence (Supplementary Tables S15A-S15B), indicating meaningful selection bias. These data cannot define a causal latency distribution.

**Supplementary Figure S6. Co-medication context and exploratory serious-outcome associations.** (A) Mutually exclusive co-medication patterns among 1,429 target reports. (B) Cluster percentages within overlapping medication subgroups. (C) Unadjusted serious-outcome odds ratios and 95% confidence intervals in 63 age/sex-complete reports (39 serious); the US-country predictor is omitted because all reports were US reports. These sparse analyses do not establish causal severity predictors. Potential-hepatotoxic and CYP2C8-related categories are screening lists, not validated interaction groupings. FDA's interaction table supports recognized CYP2C8 terms, while the REZDIFFRA label supplies gemfibrozil/clopidogrel guidance; broader enzyme modifiers remain exploratory (DailyMed, 2026; U.S. Food and Drug Administration, 2026b). Exact definitions and roles appear in Supplementary Table S2, subgroup denominators in S16, deidentified co-reports in S17, and complete-case models in S18. Co-reporting is a medication-review reminder, not proof of concurrent exposure, dose, adherence, pharmacokinetic interaction or causality.

# Supplementary Tables

**Supplementary Table S1. Full PT-to-clinical-cluster dictionary.**

Note: The nine clinical clusters were investigator defined and encoded in the analysis script before calculation of disproportionality outputs; they were not preregistered or independently adjudicated by a blinded external panel. Each PT was assigned to one dictionary cluster only. A single report could enter multiple clusters through different PTs, but it was counted only once within any given cluster. The table provides the complete PT attribution, clinical rationale, development timing, and overlap rule.

| **Clinical cluster** | **Preferred term** | **Clinical rationale** | **Development timing** | **Overlap rule** |
| --- | --- | --- | --- | --- |
| hepatocellular_injury | acute hepatic failure | Hepatocellular laboratory abnormalities and clinically coded liver injury or failure. | Encoded in the analysis script before calculation of disproportionality outputs; not preregistered. | Each PT occurs in one dictionary cluster; a report may contribute to multiple clusters through different PTs. |
| hepatocellular_injury | alanine aminotransferase increased | Hepatocellular laboratory abnormalities and clinically coded liver injury or failure. | Encoded in the analysis script before calculation of disproportionality outputs; not preregistered. | Each PT occurs in one dictionary cluster; a report may contribute to multiple clusters through different PTs. |
| hepatocellular_injury | aspartate aminotransferase increased | Hepatocellular laboratory abnormalities and clinically coded liver injury or failure. | Encoded in the analysis script before calculation of disproportionality outputs; not preregistered. | Each PT occurs in one dictionary cluster; a report may contribute to multiple clusters through different PTs. |
| hepatocellular_injury | drug-induced liver injury | Hepatocellular laboratory abnormalities and clinically coded liver injury or failure. | Encoded in the analysis script before calculation of disproportionality outputs; not preregistered. | Each PT occurs in one dictionary cluster; a report may contribute to multiple clusters through different PTs. |
| hepatocellular_injury | hepatic cytolysis | Hepatocellular laboratory abnormalities and clinically coded liver injury or failure. | Encoded in the analysis script before calculation of disproportionality outputs; not preregistered. | Each PT occurs in one dictionary cluster; a report may contribute to multiple clusters through different PTs. |
| hepatocellular_injury | hepatic enzyme increased | Hepatocellular laboratory abnormalities and clinically coded liver injury or failure. | Encoded in the analysis script before calculation of disproportionality outputs; not preregistered. | Each PT occurs in one dictionary cluster; a report may contribute to multiple clusters through different PTs. |
| hepatocellular_injury | hepatic failure | Hepatocellular laboratory abnormalities and clinically coded liver injury or failure. | Encoded in the analysis script before calculation of disproportionality outputs; not preregistered. | Each PT occurs in one dictionary cluster; a report may contribute to multiple clusters through different PTs. |
| hepatocellular_injury | hepatitis | Hepatocellular laboratory abnormalities and clinically coded liver injury or failure. | Encoded in the analysis script before calculation of disproportionality outputs; not preregistered. | Each PT occurs in one dictionary cluster; a report may contribute to multiple clusters through different PTs. |
| hepatocellular_injury | hepatitis acute | Hepatocellular laboratory abnormalities and clinically coded liver injury or failure. | Encoded in the analysis script before calculation of disproportionality outputs; not preregistered. | Each PT occurs in one dictionary cluster; a report may contribute to multiple clusters through different PTs. |
| hepatocellular_injury | hepatocellular injury | Hepatocellular laboratory abnormalities and clinically coded liver injury or failure. | Encoded in the analysis script before calculation of disproportionality outputs; not preregistered. | Each PT occurs in one dictionary cluster; a report may contribute to multiple clusters through different PTs. |
| hepatocellular_injury | hepatotoxicity | Hepatocellular laboratory abnormalities and clinically coded liver injury or failure. | Encoded in the analysis script before calculation of disproportionality outputs; not preregistered. | Each PT occurs in one dictionary cluster; a report may contribute to multiple clusters through different PTs. |
| hepatocellular_injury | liver function test increased | Hepatocellular laboratory abnormalities and clinically coded liver injury or failure. | Encoded in the analysis script before calculation of disproportionality outputs; not preregistered. | Each PT occurs in one dictionary cluster; a report may contribute to multiple clusters through different PTs. |
| hepatocellular_injury | liver injury | Hepatocellular laboratory abnormalities and clinically coded liver injury or failure. | Encoded in the analysis script before calculation of disproportionality outputs; not preregistered. | Each PT occurs in one dictionary cluster; a report may contribute to multiple clusters through different PTs. |
| hepatocellular_injury | transaminases increased | Hepatocellular laboratory abnormalities and clinically coded liver injury or failure. | Encoded in the analysis script before calculation of disproportionality outputs; not preregistered. | Each PT occurs in one dictionary cluster; a report may contribute to multiple clusters through different PTs. |
| cholestasis_biliary | bile duct obstruction | Cholestatic laboratory findings, jaundice, gallstone disease, and biliary tract complications. | Encoded in the analysis script before calculation of disproportionality outputs; not preregistered. | Each PT occurs in one dictionary cluster; a report may contribute to multiple clusters through different PTs. |
| cholestasis_biliary | bile duct stone | Cholestatic laboratory findings, jaundice, gallstone disease, and biliary tract complications. | Encoded in the analysis script before calculation of disproportionality outputs; not preregistered. | Each PT occurs in one dictionary cluster; a report may contribute to multiple clusters through different PTs. |
| cholestasis_biliary | biliary colic | Cholestatic laboratory findings, jaundice, gallstone disease, and biliary tract complications. | Encoded in the analysis script before calculation of disproportionality outputs; not preregistered. | Each PT occurs in one dictionary cluster; a report may contribute to multiple clusters through different PTs. |
| cholestasis_biliary | biliary tract disorder | Cholestatic laboratory findings, jaundice, gallstone disease, and biliary tract complications. | Encoded in the analysis script before calculation of disproportionality outputs; not preregistered. | Each PT occurs in one dictionary cluster; a report may contribute to multiple clusters through different PTs. |
| cholestasis_biliary | blood bilirubin increased | Cholestatic laboratory findings, jaundice, gallstone disease, and biliary tract complications. | Encoded in the analysis script before calculation of disproportionality outputs; not preregistered. | Each PT occurs in one dictionary cluster; a report may contribute to multiple clusters through different PTs. |
| cholestasis_biliary | cholecystitis | Cholestatic laboratory findings, jaundice, gallstone disease, and biliary tract complications. | Encoded in the analysis script before calculation of disproportionality outputs; not preregistered. | Each PT occurs in one dictionary cluster; a report may contribute to multiple clusters through different PTs. |
| cholestasis_biliary | cholecystitis acute | Cholestatic laboratory findings, jaundice, gallstone disease, and biliary tract complications. | Encoded in the analysis script before calculation of disproportionality outputs; not preregistered. | Each PT occurs in one dictionary cluster; a report may contribute to multiple clusters through different PTs. |
| cholestasis_biliary | choledocholithiasis | Cholestatic laboratory findings, jaundice, gallstone disease, and biliary tract complications. | Encoded in the analysis script before calculation of disproportionality outputs; not preregistered. | Each PT occurs in one dictionary cluster; a report may contribute to multiple clusters through different PTs. |
| cholestasis_biliary | cholelithiasis | Cholestatic laboratory findings, jaundice, gallstone disease, and biliary tract complications. | Encoded in the analysis script before calculation of disproportionality outputs; not preregistered. | Each PT occurs in one dictionary cluster; a report may contribute to multiple clusters through different PTs. |
| cholestasis_biliary | cholestasis | Cholestatic laboratory findings, jaundice, gallstone disease, and biliary tract complications. | Encoded in the analysis script before calculation of disproportionality outputs; not preregistered. | Each PT occurs in one dictionary cluster; a report may contribute to multiple clusters through different PTs. |
| cholestasis_biliary | gallbladder disease | Cholestatic laboratory findings, jaundice, gallstone disease, and biliary tract complications. | Encoded in the analysis script before calculation of disproportionality outputs; not preregistered. | Each PT occurs in one dictionary cluster; a report may contribute to multiple clusters through different PTs. |
| cholestasis_biliary | gallbladder disorder | Cholestatic laboratory findings, jaundice, gallstone disease, and biliary tract complications. | Encoded in the analysis script before calculation of disproportionality outputs; not preregistered. | Each PT occurs in one dictionary cluster; a report may contribute to multiple clusters through different PTs. |
| cholestasis_biliary | gallstone pancreatitis | Cholestatic laboratory findings, jaundice, gallstone disease, and biliary tract complications. | Encoded in the analysis script before calculation of disproportionality outputs; not preregistered. | Each PT occurs in one dictionary cluster; a report may contribute to multiple clusters through different PTs. |
| cholestasis_biliary | hyperbilirubinaemia | Cholestatic laboratory findings, jaundice, gallstone disease, and biliary tract complications. | Encoded in the analysis script before calculation of disproportionality outputs; not preregistered. | Each PT occurs in one dictionary cluster; a report may contribute to multiple clusters through different PTs. |
| cholestasis_biliary | jaundice | Cholestatic laboratory findings, jaundice, gallstone disease, and biliary tract complications. | Encoded in the analysis script before calculation of disproportionality outputs; not preregistered. | Each PT occurs in one dictionary cluster; a report may contribute to multiple clusters through different PTs. |
| gastrointestinal_intolerance | abdominal discomfort | Common gastrointestinal tolerability symptoms relevant to treatment persistence. | Encoded in the analysis script before calculation of disproportionality outputs; not preregistered. | Each PT occurs in one dictionary cluster; a report may contribute to multiple clusters through different PTs. |
| gastrointestinal_intolerance | abdominal pain | Common gastrointestinal tolerability symptoms relevant to treatment persistence. | Encoded in the analysis script before calculation of disproportionality outputs; not preregistered. | Each PT occurs in one dictionary cluster; a report may contribute to multiple clusters through different PTs. |
| gastrointestinal_intolerance | abdominal pain upper | Common gastrointestinal tolerability symptoms relevant to treatment persistence. | Encoded in the analysis script before calculation of disproportionality outputs; not preregistered. | Each PT occurs in one dictionary cluster; a report may contribute to multiple clusters through different PTs. |
| gastrointestinal_intolerance | constipation | Common gastrointestinal tolerability symptoms relevant to treatment persistence. | Encoded in the analysis script before calculation of disproportionality outputs; not preregistered. | Each PT occurs in one dictionary cluster; a report may contribute to multiple clusters through different PTs. |
| gastrointestinal_intolerance | dehydration | Common gastrointestinal tolerability symptoms relevant to treatment persistence. | Encoded in the analysis script before calculation of disproportionality outputs; not preregistered. | Each PT occurs in one dictionary cluster; a report may contribute to multiple clusters through different PTs. |
| gastrointestinal_intolerance | diarrhea | Common gastrointestinal tolerability symptoms relevant to treatment persistence. | Encoded in the analysis script before calculation of disproportionality outputs; not preregistered. | Each PT occurs in one dictionary cluster; a report may contribute to multiple clusters through different PTs. |
| gastrointestinal_intolerance | diarrhoea | Common gastrointestinal tolerability symptoms relevant to treatment persistence. | Encoded in the analysis script before calculation of disproportionality outputs; not preregistered. | Each PT occurs in one dictionary cluster; a report may contribute to multiple clusters through different PTs. |
| gastrointestinal_intolerance | dyspepsia | Common gastrointestinal tolerability symptoms relevant to treatment persistence. | Encoded in the analysis script before calculation of disproportionality outputs; not preregistered. | Each PT occurs in one dictionary cluster; a report may contribute to multiple clusters through different PTs. |
| gastrointestinal_intolerance | eructation | Common gastrointestinal tolerability symptoms relevant to treatment persistence. | Encoded in the analysis script before calculation of disproportionality outputs; not preregistered. | Each PT occurs in one dictionary cluster; a report may contribute to multiple clusters through different PTs. |
| gastrointestinal_intolerance | flatulence | Common gastrointestinal tolerability symptoms relevant to treatment persistence. | Encoded in the analysis script before calculation of disproportionality outputs; not preregistered. | Each PT occurs in one dictionary cluster; a report may contribute to multiple clusters through different PTs. |
| gastrointestinal_intolerance | gastrointestinal disorder | Common gastrointestinal tolerability symptoms relevant to treatment persistence. | Encoded in the analysis script before calculation of disproportionality outputs; not preregistered. | Each PT occurs in one dictionary cluster; a report may contribute to multiple clusters through different PTs. |
| gastrointestinal_intolerance | gastrooesophageal reflux disease | Common gastrointestinal tolerability symptoms relevant to treatment persistence. | Encoded in the analysis script before calculation of disproportionality outputs; not preregistered. | Each PT occurs in one dictionary cluster; a report may contribute to multiple clusters through different PTs. |
| gastrointestinal_intolerance | nausea | Common gastrointestinal tolerability symptoms relevant to treatment persistence. | Encoded in the analysis script before calculation of disproportionality outputs; not preregistered. | Each PT occurs in one dictionary cluster; a report may contribute to multiple clusters through different PTs. |
| gastrointestinal_intolerance | vomiting | Common gastrointestinal tolerability symptoms relevant to treatment persistence. | Encoded in the analysis script before calculation of disproportionality outputs; not preregistered. | Each PT occurs in one dictionary cluster; a report may contribute to multiple clusters through different PTs. |
| thyroid_metabolic | blood cholesterol abnormal | Thyroid-function, weight, glycaemic, and lipid terms related to THR-beta pharmacology and monitoring. | Encoded in the analysis script before calculation of disproportionality outputs; not preregistered. | Each PT occurs in one dictionary cluster; a report may contribute to multiple clusters through different PTs. |
| thyroid_metabolic | blood thyroid stimulating hormone decreased | Thyroid-function, weight, glycaemic, and lipid terms related to THR-beta pharmacology and monitoring. | Encoded in the analysis script before calculation of disproportionality outputs; not preregistered. | Each PT occurs in one dictionary cluster; a report may contribute to multiple clusters through different PTs. |
| thyroid_metabolic | blood thyroid stimulating hormone increased | Thyroid-function, weight, glycaemic, and lipid terms related to THR-beta pharmacology and monitoring. | Encoded in the analysis script before calculation of disproportionality outputs; not preregistered. | Each PT occurs in one dictionary cluster; a report may contribute to multiple clusters through different PTs. |
| thyroid_metabolic | hyperglycaemia | Thyroid-function, weight, glycaemic, and lipid terms related to THR-beta pharmacology and monitoring. | Encoded in the analysis script before calculation of disproportionality outputs; not preregistered. | Each PT occurs in one dictionary cluster; a report may contribute to multiple clusters through different PTs. |
| thyroid_metabolic | hypoglycaemia | Thyroid-function, weight, glycaemic, and lipid terms related to THR-beta pharmacology and monitoring. | Encoded in the analysis script before calculation of disproportionality outputs; not preregistered. | Each PT occurs in one dictionary cluster; a report may contribute to multiple clusters through different PTs. |
| thyroid_metabolic | lipids abnormal | Thyroid-function, weight, glycaemic, and lipid terms related to THR-beta pharmacology and monitoring. | Encoded in the analysis script before calculation of disproportionality outputs; not preregistered. | Each PT occurs in one dictionary cluster; a report may contribute to multiple clusters through different PTs. |
| thyroid_metabolic | thyroid function test abnormal | Thyroid-function, weight, glycaemic, and lipid terms related to THR-beta pharmacology and monitoring. | Encoded in the analysis script before calculation of disproportionality outputs; not preregistered. | Each PT occurs in one dictionary cluster; a report may contribute to multiple clusters through different PTs. |
| thyroid_metabolic | thyroid stimulating hormone abnormal | Thyroid-function, weight, glycaemic, and lipid terms related to THR-beta pharmacology and monitoring. | Encoded in the analysis script before calculation of disproportionality outputs; not preregistered. | Each PT occurs in one dictionary cluster; a report may contribute to multiple clusters through different PTs. |
| thyroid_metabolic | thyroxine abnormal | Thyroid-function, weight, glycaemic, and lipid terms related to THR-beta pharmacology and monitoring. | Encoded in the analysis script before calculation of disproportionality outputs; not preregistered. | Each PT occurs in one dictionary cluster; a report may contribute to multiple clusters through different PTs. |
| thyroid_metabolic | thyroxine free decreased | Thyroid-function, weight, glycaemic, and lipid terms related to THR-beta pharmacology and monitoring. | Encoded in the analysis script before calculation of disproportionality outputs; not preregistered. | Each PT occurs in one dictionary cluster; a report may contribute to multiple clusters through different PTs. |
| thyroid_metabolic | thyroxine free increased | Thyroid-function, weight, glycaemic, and lipid terms related to THR-beta pharmacology and monitoring. | Encoded in the analysis script before calculation of disproportionality outputs; not preregistered. | Each PT occurs in one dictionary cluster; a report may contribute to multiple clusters through different PTs. |
| thyroid_metabolic | weight decreased | Thyroid-function, weight, glycaemic, and lipid terms related to THR-beta pharmacology and monitoring. | Encoded in the analysis script before calculation of disproportionality outputs; not preregistered. | Each PT occurs in one dictionary cluster; a report may contribute to multiple clusters through different PTs. |
| muscle_toxicity | blood creatine phosphokinase increased | Muscle symptoms and injury terms relevant to concomitant statin exposure. | Encoded in the analysis script before calculation of disproportionality outputs; not preregistered. | Each PT occurs in one dictionary cluster; a report may contribute to multiple clusters through different PTs. |
| muscle_toxicity | muscle injury | Muscle symptoms and injury terms relevant to concomitant statin exposure. | Encoded in the analysis script before calculation of disproportionality outputs; not preregistered. | Each PT occurs in one dictionary cluster; a report may contribute to multiple clusters through different PTs. |
| muscle_toxicity | muscle spasms | Muscle symptoms and injury terms relevant to concomitant statin exposure. | Encoded in the analysis script before calculation of disproportionality outputs; not preregistered. | Each PT occurs in one dictionary cluster; a report may contribute to multiple clusters through different PTs. |
| muscle_toxicity | muscular weakness | Muscle symptoms and injury terms relevant to concomitant statin exposure. | Encoded in the analysis script before calculation of disproportionality outputs; not preregistered. | Each PT occurs in one dictionary cluster; a report may contribute to multiple clusters through different PTs. |
| muscle_toxicity | musculoskeletal pain | Muscle symptoms and injury terms relevant to concomitant statin exposure. | Encoded in the analysis script before calculation of disproportionality outputs; not preregistered. | Each PT occurs in one dictionary cluster; a report may contribute to multiple clusters through different PTs. |
| muscle_toxicity | myalgia | Muscle symptoms and injury terms relevant to concomitant statin exposure. | Encoded in the analysis script before calculation of disproportionality outputs; not preregistered. | Each PT occurs in one dictionary cluster; a report may contribute to multiple clusters through different PTs. |
| muscle_toxicity | myopathy | Muscle symptoms and injury terms relevant to concomitant statin exposure. | Encoded in the analysis script before calculation of disproportionality outputs; not preregistered. | Each PT occurs in one dictionary cluster; a report may contribute to multiple clusters through different PTs. |
| muscle_toxicity | myositis | Muscle symptoms and injury terms relevant to concomitant statin exposure. | Encoded in the analysis script before calculation of disproportionality outputs; not preregistered. | Each PT occurs in one dictionary cluster; a report may contribute to multiple clusters through different PTs. |
| muscle_toxicity | rhabdomyolysis | Muscle symptoms and injury terms relevant to concomitant statin exposure. | Encoded in the analysis script before calculation of disproportionality outputs; not preregistered. | Each PT occurs in one dictionary cluster; a report may contribute to multiple clusters through different PTs. |
| neuropsychiatric | anxiety | Neurological and psychiatric symptoms reported during postmarketing treatment. | Encoded in the analysis script before calculation of disproportionality outputs; not preregistered. | Each PT occurs in one dictionary cluster; a report may contribute to multiple clusters through different PTs. |
| neuropsychiatric | confusional state | Neurological and psychiatric symptoms reported during postmarketing treatment. | Encoded in the analysis script before calculation of disproportionality outputs; not preregistered. | Each PT occurs in one dictionary cluster; a report may contribute to multiple clusters through different PTs. |
| neuropsychiatric | depression | Neurological and psychiatric symptoms reported during postmarketing treatment. | Encoded in the analysis script before calculation of disproportionality outputs; not preregistered. | Each PT occurs in one dictionary cluster; a report may contribute to multiple clusters through different PTs. |
| neuropsychiatric | dizziness | Neurological and psychiatric symptoms reported during postmarketing treatment. | Encoded in the analysis script before calculation of disproportionality outputs; not preregistered. | Each PT occurs in one dictionary cluster; a report may contribute to multiple clusters through different PTs. |
| neuropsychiatric | headache | Neurological and psychiatric symptoms reported during postmarketing treatment. | Encoded in the analysis script before calculation of disproportionality outputs; not preregistered. | Each PT occurs in one dictionary cluster; a report may contribute to multiple clusters through different PTs. |
| neuropsychiatric | insomnia | Neurological and psychiatric symptoms reported during postmarketing treatment. | Encoded in the analysis script before calculation of disproportionality outputs; not preregistered. | Each PT occurs in one dictionary cluster; a report may contribute to multiple clusters through different PTs. |
| neuropsychiatric | suicidal ideation | Neurological and psychiatric symptoms reported during postmarketing treatment. | Encoded in the analysis script before calculation of disproportionality outputs; not preregistered. | Each PT occurs in one dictionary cluster; a report may contribute to multiple clusters through different PTs. |
| cardiovascular | atrial fibrillation | Cardiac rhythm, blood-pressure, ischaemic, and heart-failure terms. | Encoded in the analysis script before calculation of disproportionality outputs; not preregistered. | Each PT occurs in one dictionary cluster; a report may contribute to multiple clusters through different PTs. |
| cardiovascular | cardiac failure | Cardiac rhythm, blood-pressure, ischaemic, and heart-failure terms. | Encoded in the analysis script before calculation of disproportionality outputs; not preregistered. | Each PT occurs in one dictionary cluster; a report may contribute to multiple clusters through different PTs. |
| cardiovascular | hypertension | Cardiac rhythm, blood-pressure, ischaemic, and heart-failure terms. | Encoded in the analysis script before calculation of disproportionality outputs; not preregistered. | Each PT occurs in one dictionary cluster; a report may contribute to multiple clusters through different PTs. |
| cardiovascular | hypotension | Cardiac rhythm, blood-pressure, ischaemic, and heart-failure terms. | Encoded in the analysis script before calculation of disproportionality outputs; not preregistered. | Each PT occurs in one dictionary cluster; a report may contribute to multiple clusters through different PTs. |
| cardiovascular | myocardial infarction | Cardiac rhythm, blood-pressure, ischaemic, and heart-failure terms. | Encoded in the analysis script before calculation of disproportionality outputs; not preregistered. | Each PT occurs in one dictionary cluster; a report may contribute to multiple clusters through different PTs. |
| cardiovascular | palpitations | Cardiac rhythm, blood-pressure, ischaemic, and heart-failure terms. | Encoded in the analysis script before calculation of disproportionality outputs; not preregistered. | Each PT occurs in one dictionary cluster; a report may contribute to multiple clusters through different PTs. |
| cardiovascular | tachycardia | Cardiac rhythm, blood-pressure, ischaemic, and heart-failure terms. | Encoded in the analysis script before calculation of disproportionality outputs; not preregistered. | Each PT occurs in one dictionary cluster; a report may contribute to multiple clusters through different PTs. |
| renal | acute kidney injury | Acute renal injury, renal failure, and renal-function laboratory terms. | Encoded in the analysis script before calculation of disproportionality outputs; not preregistered. | Each PT occurs in one dictionary cluster; a report may contribute to multiple clusters through different PTs. |
| renal | blood creatinine increased | Acute renal injury, renal failure, and renal-function laboratory terms. | Encoded in the analysis script before calculation of disproportionality outputs; not preregistered. | Each PT occurs in one dictionary cluster; a report may contribute to multiple clusters through different PTs. |
| renal | glomerular filtration rate decreased | Acute renal injury, renal failure, and renal-function laboratory terms. | Encoded in the analysis script before calculation of disproportionality outputs; not preregistered. | Each PT occurs in one dictionary cluster; a report may contribute to multiple clusters through different PTs. |
| renal | renal failure | Acute renal injury, renal failure, and renal-function laboratory terms. | Encoded in the analysis script before calculation of disproportionality outputs; not preregistered. | Each PT occurs in one dictionary cluster; a report may contribute to multiple clusters through different PTs. |
| rash_hypersensitivity | anaphylactic reaction | Cutaneous and systemic hypersensitivity manifestations observed in clinical or postmarketing use. | Encoded in the analysis script before calculation of disproportionality outputs; not preregistered. | Each PT occurs in one dictionary cluster; a report may contribute to multiple clusters through different PTs. |
| rash_hypersensitivity | angioedema | Cutaneous and systemic hypersensitivity manifestations observed in clinical or postmarketing use. | Encoded in the analysis script before calculation of disproportionality outputs; not preregistered. | Each PT occurs in one dictionary cluster; a report may contribute to multiple clusters through different PTs. |
| rash_hypersensitivity | hypersensitivity | Cutaneous and systemic hypersensitivity manifestations observed in clinical or postmarketing use. | Encoded in the analysis script before calculation of disproportionality outputs; not preregistered. | Each PT occurs in one dictionary cluster; a report may contribute to multiple clusters through different PTs. |
| rash_hypersensitivity | pruritus | Cutaneous and systemic hypersensitivity manifestations observed in clinical or postmarketing use. | Encoded in the analysis script before calculation of disproportionality outputs; not preregistered. | Each PT occurs in one dictionary cluster; a report may contribute to multiple clusters through different PTs. |
| rash_hypersensitivity | rash | Cutaneous and systemic hypersensitivity manifestations observed in clinical or postmarketing use. | Encoded in the analysis script before calculation of disproportionality outputs; not preregistered. | Each PT occurs in one dictionary cluster; a report may contribute to multiple clusters through different PTs. |
| rash_hypersensitivity | urticaria | Cutaneous and systemic hypersensitivity manifestations observed in clinical or postmarketing use. | Encoded in the analysis script before calculation of disproportionality outputs; not preregistered. | Each PT occurs in one dictionary cluster; a report may contribute to multiple clusters through different PTs. |

**Supplementary Table S2. Exact prespecified co-medications, FAERS role codes, and CYP2C8 source framework.**

Note: These are investigator-defined screening lists, not validated hepatotoxicity or interaction groupings. The broad CYP2C8-related screen includes recognized interaction terms and exploratory enzyme modifiers; not every entry is an FDA-listed CYP2C8 inhibitor or inducer. FDA lists montelukast as a CYP2C8 substrate. Resmetirom-specific guidance for gemfibrozil and clopidogrel comes from the REZDIFFRA label (DailyMed, 2026; U.S. Food and Drug Administration, 2026b). The grouping does not establish concurrent exposure or a causal interaction.

| **Category** | **Pharmacologic subgroup** | **Drug** | **Target cases** | **% of target** | **FAERS roles** | **Definition note** |
| --- | --- | --- | --- | --- | --- | --- |
| statin |  | atorvastatin | 113 | 7.9 | C:111; I:2 | Prespecified co-medication class. |
| statin |  | rosuvastatin | 70 | 4.9 | C:64; SS:6 | Prespecified co-medication class. |
| statin |  | crestor | 26 | 1.8 | C:24; SS:2 | Prespecified co-medication class. |
| statin |  | lipitor | 26 | 1.8 | C:26 | Prespecified co-medication class. |
| statin |  | pravastatin | 17 | 1.2 | C:16; SS:1 | Prespecified co-medication class. |
| statin |  | simvastatin | 13 | 0.9 | C:13 | Prespecified co-medication class. |
| statin |  | lovastatin | 5 | 0.3 | C:5 | Prespecified co-medication class. |
| statin |  | pitavastatin | 2 | 0.1 | C:2 | Prespecified co-medication class. |
| statin |  | mevacor | 1 | 0.1 | C:1 | Prespecified co-medication class. |
| statin |  | pravachol | 1 | 0.1 | C:1 | Prespecified co-medication class. |
| statin |  | livalo | 1 | 0.1 | C:1 | Prespecified co-medication class. |
| statin |  | zocor | 1 | 0.1 | C:1 | Prespecified co-medication class. |
| glp1_gip |  | mounjaro | 58 | 4.1 | C:53; SS:5 | Prespecified co-medication class. |
| glp1_gip |  | ozempic | 46 | 3.2 | C:43; PS:1; SS:2 | Prespecified co-medication class. |
| glp1_gip |  | trulicity | 18 | 1.3 | C:13; PS:1; SS:4 | Prespecified co-medication class. |
| glp1_gip |  | wegovy | 13 | 0.9 | C:11; SS:2 | Prespecified co-medication class. |
| glp1_gip |  | semaglutide | 13 | 0.9 | C:13 | Prespecified co-medication class. |
| glp1_gip |  | tirzepatide | 9 | 0.6 | C:8; SS:1 | Prespecified co-medication class. |
| glp1_gip |  | zepbound | 6 | 0.4 | C:6 | Prespecified co-medication class. |
| glp1_gip |  | rybelsus | 5 | 0.3 | C:5 | Prespecified co-medication class. |
| glp1_gip |  | victoza | 2 | 0.1 | C:2 | Prespecified co-medication class. |
| glp1_gip |  | liraglutide | 1 | 0.1 | C:1 | Prespecified co-medication class. |
| sglt2 |  | jardiance | 35 | 2.4 | C:35 | Prespecified co-medication class. |
| sglt2 |  | farxiga | 16 | 1.1 | C:15; SS:1 | Prespecified co-medication class. |
| sglt2 |  | empagliflozin | 8 | 0.6 | C:8 | Prespecified co-medication class. |
| sglt2 |  | invokana | 2 | 0.1 | C:2 | Prespecified co-medication class. |
| sglt2 |  | dapagliflozin | 1 | 0.1 | C:1 | Prespecified co-medication class. |
| hepatotoxic |  | allopurinol | 31 | 2.2 | C:31 | Investigator-defined exploratory list; exact terms disclosed for reproducibility. |
| hepatotoxic |  | diclofenac | 19 | 1.3 | C:19 | Investigator-defined exploratory list; exact terms disclosed for reproducibility. |
| hepatotoxic |  | ketoconazole | 10 | 0.7 | C:10 | Investigator-defined exploratory list; exact terms disclosed for reproducibility. |
| hepatotoxic |  | fluconazole | 5 | 0.3 | C:3; SS:2 | Investigator-defined exploratory list; exact terms disclosed for reproducibility. |
| hepatotoxic |  | tamoxifen | 3 | 0.2 | C:3 | Investigator-defined exploratory list; exact terms disclosed for reproducibility. |
| hepatotoxic |  | carbamazepine | 3 | 0.2 | C:3 | Investigator-defined exploratory list; exact terms disclosed for reproducibility. |
| hepatotoxic |  | azathioprine | 3 | 0.2 | C:2; SS:1 | Investigator-defined exploratory list; exact terms disclosed for reproducibility. |
| hepatotoxic |  | nitrofurantoin | 3 | 0.2 | C:3 | Investigator-defined exploratory list; exact terms disclosed for reproducibility. |
| hepatotoxic |  | amiodarone | 3 | 0.2 | C:3 | Investigator-defined exploratory list; exact terms disclosed for reproducibility. |
| hepatotoxic |  | minocycline | 2 | 0.1 | C:2 | Investigator-defined exploratory list; exact terms disclosed for reproducibility. |
| hepatotoxic |  | methotrexate | 2 | 0.1 | C:1; SS:1 | Investigator-defined exploratory list; exact terms disclosed for reproducibility. |
| hepatotoxic |  | phenytoin | 2 | 0.1 | C:2 | Investigator-defined exploratory list; exact terms disclosed for reproducibility. |
| hepatotoxic |  | infliximab | 1 | 0.1 | C:1 | Investigator-defined exploratory list; exact terms disclosed for reproducibility. |
| hepatotoxic |  | methimazole | 1 | 0.1 | C:1 | Investigator-defined exploratory list; exact terms disclosed for reproducibility. |
| cyp2c8 | CYP2C8 substrate | montelukast | 35 | 2.4 | C:35 | FDA example of a moderately sensitive CYP2C8 substrate; not an inhibitor or confirmed resmetirom interaction. |
| cyp2c8 | moderate_inhibitor_label_example | clopidogrel | 25 | 1.7 | C:25 | FDA CYP interaction framework and current REZDIFFRA label; co-reporting is a medication-review reminder, not interaction evidence. |
| cyp2c8 | Exploratory modifier | carbamazepine | 3 | 0.2 | C:3 | Investigator screen, not FDA classification; no confirmed resmetirom interaction. |
| cyp2c8 | Exploratory modifier | trimethoprim | 3 | 0.2 | C:3 | Investigator screen, not FDA classification; no confirmed resmetirom interaction. |
| cyp2c8 | strong_inhibitor_label_example | gemfibrozil | 3 | 0.2 | C:3 | FDA CYP interaction framework and current REZDIFFRA label; co-reporting is a medication-review reminder, not interaction evidence. |
| cyp2c8 | Exploratory modifier | phenytoin | 2 | 0.1 | C:2 | Investigator screen, not FDA classification; no confirmed resmetirom interaction. |
| cyp2c8 | Exploratory modifier | rifampin | 1 | 0.1 | C:1 | Investigator screen, not FDA classification; no confirmed resmetirom interaction. |
| cyp2c8 | Exploratory modifier | deferasirox | 0 | 0.0 |  | Investigator screen, not FDA classification; no confirmed resmetirom interaction. |
| cyp2c8 | Exploratory modifier | rifampicin | 0 | 0.0 |  | Investigator screen, not FDA classification; no confirmed resmetirom interaction. |
| cyp2c8 | Exploratory modifier | phenobarbital | 0 | 0.0 |  | Investigator screen, not FDA classification; no confirmed resmetirom interaction. |
| cyp2c8 | Exploratory modifier | efavirenz | 0 | 0.0 |  | Investigator screen, not FDA classification; no confirmed resmetirom interaction. |

**Supplementary Table S3. Case selection and data processing flow.**

| **Step** | **Reports/cases, n** |
| --- | --- |
| DEMO rows in selected quarterly files | 3,634,333 |
| Unique CASEID after latest FDA_DT/caseversion deduplication and DELETE exclusion | 3,155,054 |
| Cases with any resmetirom role | 1,519 |
| Cases with resmetirom primary or secondary suspect | 1,429 |
| Main-analysis resmetirom PS/SS cases with at least one PT | 1,429 |
| Reference non-resmetirom cases with at least one PT | 3,153,534 |

Note: FAERS quarterly ASCII files were deduplicated by latest FDA_DT/case version and DELETE exclusions before target-drug selection. After case-level deduplication, each unique case contributed no more than once to a given PT, primary SOC, or clinical cluster, regardless of duplicate resmetirom DRUG rows or a combined PS/SS role pattern.

**Supplementary Table S4. Complete characteristics of the main analysis set.**

| **Variable** | **Category** | **n** | **%** | **Note** |
| --- | --- | --- | --- | --- |
| Age | median (IQR), years |  |  | 63.0 (54.0-70.0); mean 61.4, SD 12.0; non-missing n=163 |
| Sex | Unknown | 1,352 | 94.6 |  |
| Sex | F | 49 | 3.4 |  |
| Sex | M | 27 | 1.9 |  |
| Sex | UNK | 1 | 0.1 |  |
| Country/region of occurrence | US | 1,429 | 100.0 |  |
| Reporter occupation | Consumer | 558 | 39.0 |  |
| Reporter occupation | Other health professional | 415 | 29.0 |  |
| Reporter occupation | Physician | 271 | 19.0 |  |
| Reporter occupation | Unknown | 157 | 11.0 |  |
| Reporter occupation | Pharmacist | 28 | 2.0 |  |
| RPSR report source | Unknown/no RPSR row | 1,358 | 95.0 | Case-level source-code count; cases may have multiple source codes. |
| RPSR report source | Consumer | 44 | 3.1 | Case-level source-code count; cases may have multiple source codes. |
| RPSR report source | Health professional | 27 | 1.9 | Case-level source-code count; cases may have multiple source codes. |
| Resmetirom role combination | PS | 1,220 | 85.4 |  |
| Resmetirom role combination | PS+SS | 186 | 13.0 |  |
| Resmetirom role combination | SS | 22 | 1.5 |  |
| Resmetirom role combination | C+PS | 1 | 0.1 |  |
| FDA report quarter | 2024q2 | 2 | 0.1 |  |
| FDA report quarter | 2024q3 | 109 | 7.6 |  |
| FDA report quarter | 2024q4 | 141 | 9.9 |  |
| FDA report quarter | 2025q1 | 119 | 8.3 |  |
| FDA report quarter | 2025q2 | 151 | 10.6 |  |
| FDA report quarter | 2025q3 | 327 | 22.9 |  |
| FDA report quarter | 2025q4 | 263 | 18.4 |  |
| FDA report quarter | 2026q1 | 317 | 22.2 |  |
| Serious outcome | Any serious outcome code | 426 | 29.8 |  |
| Serious outcome code rows | Death | 65 | 11.5 | Percent denominator is outcome-code rows, not cases. |
| Serious outcome code rows | Life-threatening | 8 | 1.4 | Percent denominator is outcome-code rows, not cases. |
| Serious outcome code rows | Hospitalization | 88 | 15.5 | Percent denominator is outcome-code rows, not cases. |
| Serious outcome code rows | Disability | 7 | 1.2 | Percent denominator is outcome-code rows, not cases. |
| Serious outcome code rows | Congenital anomaly | 0 | 0.0 | Percent denominator is outcome-code rows, not cases. |
| Serious outcome code rows | Required intervention | 4 | 0.7 | Percent denominator is outcome-code rows, not cases. |
| Serious outcome code rows | Other serious | 395 | 69.7 | Percent denominator is outcome-code rows, not cases. |
| Co-medication flag | Statin | 267 | 18.7 |  |
| Co-medication flag | GLP-1RA/GIP-GLP-1RA | 163 | 11.4 |  |
| Co-medication flag | SGLT2 inhibitor | 62 | 4.3 |  |
| Co-medication flag | Potential hepatotoxic drug | 79 | 5.5 |  |
| Co-medication flag | CYP2C8-related drug | 70 | 4.9 |  |

*Percentages use n=1,429 target PS/SS reports unless otherwise noted.*

**Supplementary Table S5. Report-source and target-drug name audit table.**

| **Domain** | **Item** | **n** | **%** | **Note** |
| --- | --- | --- | --- | --- |
| Report source | Unknown/no RPSR row | 1,358 | 95.0 | Case-level source-code count; cases may have multiple source codes. |
| Report source | Consumer | 44 | 3.1 | Case-level source-code count; cases may have multiple source codes. |
| Report source | Health professional | 27 | 1.9 | Case-level source-code count; cases may have multiple source codes. |
| Drug name variant | REZDIFFRA | 1,753 |  | Rows in DRUG table |
| Drug name variant | RESMETIROM | 6 |  | Rows in DRUG table |

*This table documents source-code completeness and target-drug string variants.*

**Supplementary Table S6A. Quarterly reporting trend.**

| **Quarter** | **All latest FAERS reports** | **Any-role resmetirom cases** | **PS/SS resmetirom cases** | **PS/SS with PT** | **PS/SS reports per 100,000 latest FAERS reports** |
| --- | --- | --- | --- | --- | --- |
| 2024q1 | 335,787 | 0 | 0 | 0 | 0.00 |
| 2024q2 | 329,816 | 2 | 2 | 2 | 0.61 |
| 2024q3 | 344,230 | 109 | 109 | 109 | 31.66 |
| 2024q4 | 335,086 | 144 | 141 | 141 | 42.08 |
| 2025q1 | 327,328 | 126 | 119 | 119 | 36.35 |
| 2025q2 | 330,587 | 164 | 151 | 151 | 45.68 |
| 2025q3 | 397,848 | 347 | 327 | 327 | 82.19 |
| 2025q4 | 357,148 | 282 | 263 | 263 | 73.64 |
| 2026q1 | 397,224 | 345 | 317 | 317 | 79.80 |

*Quarterly counts are based on deduplicated latest FAERS reports in each quarterly file.*

**Supplementary Table S6B. Reported indications for resmetirom-associated reports.**

| **Reported indication** | **Reports, n** |
| --- | --- |
| Metabolic dysfunction-associated steatohepatitis | 806 |
| Product used for unknown indication | 306 |
| Non-alcoholic steatohepatitis | 124 |
| Hepatic steatosis | 80 |
| Hepatic fibrosis | 62 |
| Hepatic cirrhosis | 35 |
| Liver disorder | 9 |
| Metabolic dysfunction-associated liver disease | 7 |
| Steatohepatitis | 6 |
| Liver function test abnormal | 4 |
| Non-alcoholic fatty liver | 3 |
| Product use in unapproved indication | 2 |
| Enzyme level abnormal | 2 |
| Fibrosis | 2 |
| Illness | 1 |
| Gastrointestinal inflammation | 1 |
| Metabolic disorder | 1 |
| Uterine leiomyoma | 1 |
| Biliary tract disorder | 1 |
| Hepatitis | 1 |
| Hepatitis C | 1 |
| Blood test abnormal | 1 |
| Alcoholic liver disease | 1 |
| Enzyme abnormality | 1 |
| Hepatitis B | 1 |
| Autoimmune hepatitis | 1 |

*Reported indications are not mutually exclusive and may include unknown or off-label indication terms.*

**Supplementary Table S7. Complete event-frequency table by PT, SOC, and predefined clinical cluster.**

| **Level** | **Term** | **Target cases, n** | **% of target reports** |
| --- | --- | --- | --- |
| Preferred term | Diarrhoea | 253 | 17.7 |
| Preferred term | Nausea | 226 | 15.8 |
| Preferred term | Pruritus | 221 | 15.5 |
| Preferred term | Alanine aminotransferase increased | 128 | 9.0 |
| Preferred term | Aspartate aminotransferase increased | 123 | 8.6 |
| Preferred term | Fatigue | 107 | 7.5 |
| Preferred term | Abdominal pain upper | 101 | 7.1 |
| Preferred term | Dizziness | 93 | 6.5 |
| Preferred term | Vomiting | 89 | 6.2 |
| Preferred term | Weight decreased | 85 | 5.9 |
| Preferred term | Hepatic cirrhosis | 71 | 5.0 |
| Preferred term | Hepatic enzyme increased | 64 | 4.5 |
| Preferred term | Abdominal pain | 63 | 4.4 |
| Preferred term | Therapy interrupted | 61 | 4.3 |
| Preferred term | Constipation | 60 | 4.2 |
| Preferred term | Blood bilirubin increased | 55 | 3.8 |
| Preferred term | Rash | 55 | 3.8 |
| Preferred term | Headache | 50 | 3.5 |
| Preferred term | Product use in unapproved indication | 49 | 3.4 |
| Preferred term | Abdominal discomfort | 45 | 3.1 |
| Preferred term | Death | 45 | 3.1 |
| Preferred term | Disease progression | 37 | 2.6 |
| Preferred term | No adverse event | 34 | 2.4 |
| Preferred term | Blood alkaline phosphatase increased | 32 | 2.2 |
| Preferred term | Yellow skin | 30 | 2.1 |
| Preferred term | Ocular icterus | 29 | 2.0 |
| Preferred term | Decreased appetite | 28 | 2.0 |
| Preferred term | Insomnia | 27 | 1.9 |
| Preferred term | Unevaluable event | 27 | 1.9 |
| Preferred term | Jaundice | 26 | 1.8 |
| Preferred term | Malaise | 24 | 1.7 |
| Preferred term | Asthenia | 24 | 1.7 |
| Preferred term | Metabolic dysfunction-associated steatohepatitis | 24 | 1.7 |
| Preferred term | Flatulence | 23 | 1.6 |
| Preferred term | Condition aggravated | 23 | 1.6 |
| Preferred term | Pain | 23 | 1.6 |
| Preferred term | Drug ineffective | 22 | 1.5 |
| Preferred term | Chromaturia | 22 | 1.5 |
| Preferred term | Adverse event | 22 | 1.5 |
| Preferred term | Urticaria | 22 | 1.5 |
| Preferred term | Blood glucose increased | 20 | 1.4 |
| Preferred term | Back pain | 20 | 1.4 |
| Preferred term | Abdominal distension | 19 | 1.3 |
| Preferred term | Hepatic pain | 18 | 1.3 |
| Preferred term | Weight increased | 18 | 1.3 |
| Preferred term | Dyspnoea | 17 | 1.2 |
| Preferred term | Somnolence | 17 | 1.2 |
| Preferred term | Liver function test increased | 17 | 1.2 |
| Preferred term | Illness | 17 | 1.2 |
| Preferred term | Hypersensitivity | 15 | 1.0 |
| Preferred term | Liver disorder | 14 | 1.0 |
| Preferred term | Hepatic failure | 14 | 1.0 |
| Preferred term | Muscle spasms | 14 | 1.0 |
| Preferred term | Therapeutic response changed | 13 | 0.9 |
| Preferred term | Inappropriate schedule of product administration | 12 | 0.8 |
| Preferred term | Alopecia | 12 | 0.8 |
| Preferred term | Feeling abnormal | 12 | 0.8 |
| Preferred term | Anxiety | 12 | 0.8 |
| Preferred term | Arthralgia | 12 | 0.8 |
| Preferred term | Therapeutic response unexpected | 12 | 0.8 |
| Preferred term | Liver injury | 12 | 0.8 |
| Preferred term | Syncope | 11 | 0.8 |
| Preferred term | Blood pressure increased | 11 | 0.8 |
| Preferred term | Chest pain | 11 | 0.8 |
| Preferred term | Cholelithiasis | 11 | 0.8 |
| Preferred term | Brain fog | 11 | 0.8 |
| Preferred term | Drug hypersensitivity | 11 | 0.8 |
| Preferred term | Platelet count decreased | 11 | 0.8 |
| Preferred term | Rash pruritic | 11 | 0.8 |
| Preferred term | Hepatic fibrosis | 11 | 0.8 |
| Preferred term | Faeces discoloured | 10 | 0.7 |
| Preferred term | Depression | 10 | 0.7 |
| Preferred term | Product dose omission issue | 9 | 0.6 |
| Preferred term | International normalised ratio increased | 9 | 0.6 |
| Preferred term | Dyspepsia | 9 | 0.6 |
| Preferred term | Myalgia | 9 | 0.6 |
| Preferred term | Pancreatitis | 9 | 0.6 |
| Preferred term | Off label use | 9 | 0.6 |
| Preferred term | Pain in extremity | 9 | 0.6 |
| Preferred term | Tremor | 9 | 0.6 |
| Preferred term | Hepatic fibrosis marker increased | 9 | 0.6 |
| Preferred term | Discomfort | 8 | 0.6 |
| Preferred term | Nasopharyngitis | 8 | 0.6 |
| Preferred term | Erythema | 8 | 0.6 |
| Preferred term | Palpitations | 8 | 0.6 |
| Preferred term | Adverse drug reaction | 8 | 0.6 |
| Preferred term | Pyrexia | 8 | 0.6 |
| Preferred term | Drug-induced liver injury | 8 | 0.6 |
| Preferred term | Hepatotoxicity | 8 | 0.6 |
| Preferred term | Anal incontinence | 8 | 0.6 |
| Preferred term | Wrong technique in product usage process | 8 | 0.6 |
| Preferred term | Lethargy | 7 | 0.5 |
| Preferred term | Hypoglycaemia | 7 | 0.5 |
| Preferred term | Rash erythematous | 7 | 0.5 |
| Preferred term | Transaminases increased | 7 | 0.5 |
| Preferred term | Pneumonia | 7 | 0.5 |
| Preferred term | Hyperhidrosis | 7 | 0.5 |
| Preferred term | Hepatic enzyme decreased | 7 | 0.5 |
| Preferred term | COVID-19 | 7 | 0.5 |
| Preferred term | Drug interaction | 7 | 0.5 |
| System organ class | Gastrointestinal disorders | 548 | 38.3 |
| System organ class | General disorders and administration site conditions | 457 | 32.0 |
| System organ class | Investigations | 438 | 30.7 |
| System organ class | Skin and subcutaneous tissue disorders | 342 | 23.9 |
| System organ class | Hepatobiliary disorders | 258 | 18.1 |
| System organ class | Nervous system disorders | 217 | 15.2 |
| System organ class | Injury, poisoning and procedural complications | 134 | 9.4 |
| System organ class | Musculoskeletal and connective tissue disorders | 86 | 6.0 |
| System organ class | Psychiatric disorders | 77 | 5.4 |
| System organ class | Surgical and medical procedures | 77 | 5.4 |
| System organ class | Infections and infestations | 68 | 4.8 |
| System organ class | Metabolism and nutrition disorders | 64 | 4.5 |
| System organ class | Renal and urinary disorders | 52 | 3.6 |
| System organ class | Respiratory, thoracic and mediastinal disorders | 43 | 3.0 |
| System organ class | Immune system disorders | 33 | 2.3 |
| System organ class | Eye disorders | 32 | 2.2 |
| System organ class | Neoplasms benign, malignant and unspecified (incl cysts and polyps) | 20 | 1.4 |
| System organ class | Cardiac disorders | 19 | 1.3 |
| System organ class | Vascular disorders | 18 | 1.3 |
| System organ class | Blood and lymphatic system disorders | 16 | 1.1 |
| System organ class | Social circumstances | 11 | 0.8 |
| System organ class | Reproductive system and breast disorders | 8 | 0.6 |
| System organ class | Ear and labyrinth disorders | 8 | 0.6 |
| System organ class | Endocrine disorders | 5 | 0.3 |
| System organ class | Product issues | 4 | 0.3 |
| System organ class | Congenital, familial and genetic disorders | 2 | 0.1 |
| Clinical event cluster | Hepatocellular injury | 261 | 18.3 |
| Clinical event cluster | Cholestasis/biliary events | 89 | 6.2 |
| Clinical event cluster | Gastrointestinal intolerance | 486 | 34.0 |
| Clinical event cluster | Thyroid/metabolic events | 103 | 7.2 |
| Clinical event cluster | Muscle toxicity | 34 | 2.4 |
| Clinical event cluster | Neuropsychiatric events | 171 | 12.0 |
| Clinical event cluster | Cardiovascular events | 19 | 1.3 |
| Clinical event cluster | Renal events | 10 | 0.7 |
| Clinical event cluster | Rash/hypersensitivity | 272 | 19.0 |

Note: Rows are case-level counts. After deduplication, a unique case was counted once within each PT, SOC, or clinical cluster regardless of repeated resmetirom DRUG records or PS/SS role combinations; one case could contribute to multiple different PTs, SOCs, or clusters.

**Supplementary Table S8. Complete SOC-level disproportionality results.**

| **Event** | **a** | **c** | **ROR (95% CI)** | **PRR** | **Chi-square** | **IC** | **IC025** | **Criteria met** | **All 3 criteria** |
| --- | --- | --- | --- | --- | --- | --- | --- | --- | --- |
| Gastrointestinal disorders | 548 | 479527 | 3.47 (3.12-3.86) | 2.52 | 592.96 | 1.33 | 1.18 | 3 | Yes |
| General disorders and administration site conditions | 457 | 1161805 | 0.81 (0.72-0.90) | 0.87 | 14.51 | -0.20 | -0.36 | 0 | No |
| Investigations | 438 | 351080 | 3.53 (3.15-3.95) | 2.75 | 549.60 | 1.45 | 1.30 | 3 | Yes |
| Skin and subcutaneous tissue disorders | 342 | 370802 | 2.36 (2.09-2.67) | 2.04 | 203.96 | 1.02 | 0.85 | 3 | Yes |
| Hepatobiliary disorders | 258 | 77914 | 8.70 (7.60-9.95) | 7.31 | 1435.58 | 2.83 | 2.63 | 3 | Yes |
| Nervous system disorders | 217 | 479908 | 1.00 (0.86-1.15) | 1.00 | 0.00 | -0.00 | -0.21 | 0 | No |
| Injury, poisoning and procedural complications | 134 | 997506 | 0.22 (0.19-0.27) | 0.30 | 327.16 | -1.75 | -2.01 | 0 | No |
| Musculoskeletal and connective tissue disorders | 86 | 289676 | 0.63 (0.51-0.79) | 0.66 | 17.18 | -0.61 | -0.92 | 0 | No |
| Surgical and medical procedures | 77 | 155123 | 1.10 (0.87-1.39) | 1.10 | 0.67 | 0.13 | -0.21 | 0 | No |
| Psychiatric disorders | 77 | 270660 | 0.61 (0.48-0.76) | 0.63 | 18.58 | -0.67 | -1.00 | 0 | No |
| Infections and infestations | 68 | 390576 | 0.35 (0.28-0.45) | 0.38 | 76.58 | -1.37 | -1.72 | 0 | No |
| Metabolism and nutrition disorders | 64 | 160622 | 0.87 (0.68-1.12) | 0.88 | 1.12 | -0.18 | -0.55 | 0 | No |
| Renal and urinary disorders | 52 | 121194 | 0.94 (0.72-1.25) | 0.95 | 0.16 | -0.08 | -0.48 | 0 | No |
| Respiratory, thoracic and mediastinal disorders | 43 | 299269 | 0.30 (0.22-0.40) | 0.32 | 69.87 | -1.64 | -2.08 | 0 | No |
| Immune system disorders | 33 | 107490 | 0.67 (0.47-0.95) | 0.68 | 5.24 | -0.55 | -1.05 | 0 | No |
| Eye disorders | 32 | 147964 | 0.47 (0.33-0.66) | 0.48 | 19.22 | -1.04 | -1.55 | 0 | No |
| Neoplasms benign, malignant and unspecified (incl cysts and polyps) | 20 | 144181 | 0.30 (0.19-0.46) | 0.31 | 32.96 | -1.66 | -2.29 | 0 | No |
| Cardiac disorders | 19 | 144400 | 0.28 (0.18-0.44) | 0.29 | 34.53 | -1.73 | -2.38 | 0 | No |
| Vascular disorders | 18 | 159911 | 0.24 (0.15-0.38) | 0.25 | 43.11 | -1.95 | -2.62 | 0 | No |
| Blood and lymphatic system disorders | 16 | 138429 | 0.25 (0.15-0.40) | 0.26 | 36.40 | -1.91 | -2.61 | 0 | No |
| Social circumstances | 11 | 46547 | 0.52 (0.29-0.94) | 0.52 | 4.90 | -0.88 | -1.72 | 0 | No |
| Ear and labyrinth disorders | 8 | 34761 | 0.51 (0.25-1.01) | 0.51 | 3.86 | -0.90 | -1.86 | 0 | No |
| Reproductive system and breast disorders | 8 | 47421 | 0.37 (0.18-0.74) | 0.37 | 8.59 | -1.32 | -2.29 | 0 | No |
| Endocrine disorders | 5 | 26342 | 0.42 (0.17-1.00) | 0.42 | 4.06 | -1.11 | -2.29 | 0 | No |
| Product issues | 4 | 184346 | 0.05 (0.02-0.12) | 0.05 | 80.42 | -4.08 | -5.37 | 0 | No |
| Congenital, familial and genetic disorders | 2 | 18671 | 0.24 (0.06-0.94) | 0.24 | 4.96 | -1.66 | -3.33 | 0 | No |

*Note: a=target reports with event; b=target reports without event; c=reference reports with event; d=reference reports without event.*

**Supplementary Table S9. PT-level disproportionality results for PTs with at least three target reports.**

| **Event** | **a** | **c** | **ROR (95% CI)** | **PRR** | **Chi-square** | **IC** | **IC025** | **Criteria met** | **All 3 criteria** |
| --- | --- | --- | --- | --- | --- | --- | --- | --- | --- |
| Diarrhoea | 253 | 107198 | 6.11 (5.34-7.00) | 5.21 | 888.52 | 2.35 | 2.16 | 3 | Yes |
| Nausea | 226 | 114528 | 4.98 (4.32-5.75) | 4.35 | 604.92 | 2.10 | 1.89 | 3 | Yes |
| Pruritus | 221 | 80818 | 6.96 (6.03-8.03) | 6.03 | 950.15 | 2.56 | 2.35 | 3 | Yes |
| Alanine aminotransferase increased | 128 | 7517 | 41.18 (34.29-49.44) | 37.58 | 4491.94 | 4.85 | 4.59 | 3 | Yes |
| Aspartate aminotransferase increased | 123 | 6433 | 46.07 (38.24-55.52) | 42.19 | 4864.15 | 4.96 | 4.69 | 3 | Yes |
| Fatigue | 107 | 126760 | 1.93 (1.59-2.35) | 1.86 | 44.51 | 0.88 | 0.60 | 2 | No |
| Abdominal pain upper | 101 | 31743 | 7.48 (6.11-9.16) | 7.02 | 525.22 | 2.72 | 2.43 | 3 | Yes |
| Dizziness | 93 | 69140 | 3.11 (2.52-3.83) | 2.97 | 123.95 | 1.54 | 1.23 | 3 | Yes |
| Vomiting | 89 | 71422 | 2.87 (2.31-3.55) | 2.75 | 101.28 | 1.43 | 1.12 | 3 | Yes |
| Weight decreased | 85 | 41962 | 4.69 (3.77-5.84) | 4.47 | 231.61 | 2.10 | 1.78 | 3 | Yes |
| Hepatic cirrhosis | 71 | 3235 | 50.91 (40.01-64.79) | 48.43 | 3230.82 | 4.85 | 4.50 | 3 | Yes |
| Hepatic enzyme increased | 64 | 12171 | 12.10 (9.41-15.56) | 11.60 | 619.35 | 3.31 | 2.94 | 3 | Yes |
| Abdominal pain | 63 | 35473 | 4.05 (3.15-5.22) | 3.92 | 138.30 | 1.90 | 1.53 | 3 | Yes |
| Therapy interrupted | 61 | 27240 | 5.12 (3.96-6.62) | 4.94 | 193.04 | 2.21 | 1.84 | 3 | Yes |
| Constipation | 60 | 39835 | 3.43 (2.64-4.44) | 3.32 | 98.59 | 1.68 | 1.30 | 3 | Yes |
| Blood bilirubin increased | 55 | 3006 | 41.95 (31.97-55.06) | 40.38 | 2076.19 | 4.55 | 4.16 | 3 | Yes |
| Rash | 55 | 73725 | 1.67 (1.28-2.19) | 1.65 | 14.28 | 0.70 | 0.31 | 2 | No |
| Headache | 50 | 88507 | 1.26 (0.95-1.67) | 1.25 | 2.51 | 0.31 | -0.10 | 0 | No |
| Product use in unapproved indication | 49 | 64263 | 1.71 (1.28-2.27) | 1.68 | 13.84 | 0.73 | 0.31 | 2 | No |
| Abdominal discomfort | 45 | 26881 | 3.78 (2.81-5.09) | 3.69 | 89.04 | 1.80 | 1.37 | 3 | Yes |
| Death | 45 | 119382 | 0.83 (0.61-1.11) | 0.83 | 1.59 | -0.26 | -0.69 | 0 | No |
| Disease progression | 37 | 19992 | 4.17 (3.00-5.78) | 4.08 | 86.57 | 1.91 | 1.44 | 3 | Yes |
| No adverse event | 34 | 47095 | 1.61 (1.14-2.26) | 1.59 | 7.62 | 0.65 | 0.15 | 2 | No |
| Blood alkaline phosphatase increased | 32 | 2503 | 28.84 (20.27-41.03) | 28.21 | 830.02 | 3.94 | 3.43 | 3 | Yes |
| Yellow skin | 30 | 523 | 129.28 (89.15-187.48) | 126.59 | 3535.66 | 4.63 | 4.09 | 3 | Yes |
| Ocular icterus | 29 | 434 | 150.49 (102.96-219.97) | 147.46 | 3954.88 | 4.63 | 4.08 | 3 | Yes |
| Decreased appetite | 28 | 39424 | 1.58 (1.09-2.30) | 1.57 | 5.82 | 0.62 | 0.08 | 2 | No |
| Unevaluable event | 27 | 7339 | 8.26 (5.64-12.09) | 8.12 | 168.31 | 2.69 | 2.14 | 3 | Yes |
| Insomnia | 27 | 34256 | 1.75 (1.20-2.57) | 1.74 | 8.57 | 0.76 | 0.21 | 2 | No |
| Jaundice | 26 | 2638 | 22.13 (14.99-32.69) | 21.75 | 510.11 | 3.61 | 3.05 | 3 | Yes |
| Metabolic dysfunction-associated steatohepatitis | 24 | 143 | 376.68 (243.69-582.26) | 370.37 | 7570.87 | 4.54 | 3.91 | 3 | Yes |
| Asthenia | 24 | 55228 | 0.96 (0.64-1.43) | 0.96 | 0.04 | -0.06 | -0.64 | 0 | No |
| Malaise | 24 | 55348 | 0.96 (0.64-1.43) | 0.96 | 0.05 | -0.06 | -0.64 | 0 | No |
| Flatulence | 23 | 8757 | 5.87 (3.89-8.87) | 5.80 | 91.29 | 2.27 | 1.67 | 3 | Yes |
| Condition aggravated | 23 | 64234 | 0.79 (0.52-1.19) | 0.79 | 1.31 | -0.33 | -0.92 | 0 | No |
| Pain | 23 | 79192 | 0.64 (0.42-0.96) | 0.64 | 4.74 | -0.62 | -1.21 | 0 | No |
| Chromaturia | 22 | 2665 | 18.49 (12.11-28.22) | 18.22 | 355.37 | 3.37 | 2.77 | 3 | Yes |
| Adverse event | 22 | 12892 | 3.81 (2.50-5.81) | 3.77 | 44.80 | 1.75 | 1.14 | 3 | Yes |
| Urticaria | 22 | 28203 | 1.73 (1.14-2.64) | 1.72 | 6.71 | 0.74 | 0.13 | 2 | No |
| Drug ineffective | 22 | 185056 | 0.25 (0.16-0.38) | 0.26 | 48.47 | -1.88 | -2.49 | 0 | No |
| Blood glucose increased | 20 | 20205 | 2.20 (1.42-3.42) | 2.18 | 12.91 | 1.05 | 0.41 | 3 | Yes |
| Back pain | 20 | 32333 | 1.37 (0.88-2.13) | 1.37 | 1.97 | 0.42 | -0.21 | 0 | No |
| Abdominal distension | 19 | 16647 | 2.54 (1.61-3.99) | 2.52 | 17.47 | 1.23 | 0.58 | 3 | Yes |
| Hepatic pain | 18 | 608 | 66.15 (41.28-106.02) | 65.33 | 1107.71 | 3.89 | 3.21 | 3 | Yes |
| Weight increased | 18 | 35146 | 1.13 (0.71-1.80) | 1.13 | 0.27 | 0.17 | -0.50 | 0 | No |
| Liver function test increased | 17 | 5018 | 7.55 (4.68-12.20) | 7.48 | 95.20 | 2.46 | 1.77 | 3 | Yes |
| Somnolence | 17 | 27945 | 1.35 (0.83-2.17) | 1.34 | 1.50 | 0.40 | -0.29 | 0 | No |
| Illness | 17 | 41923 | 0.89 (0.55-1.44) | 0.89 | 0.21 | -0.15 | -0.84 | 0 | No |
| Dyspnoea | 17 | 81362 | 0.45 (0.28-0.73) | 0.46 | 10.99 | -1.07 | -1.76 | 0 | No |
| Hypersensitivity | 15 | 26236 | 1.26 (0.76-2.10) | 1.26 | 0.82 | 0.31 | -0.41 | 0 | No |
| Hepatic failure | 14 | 3487 | 8.94 (5.27-15.15) | 8.86 | 97.34 | 2.54 | 1.78 | 3 | Yes |
| Liver disorder | 14 | 7060 | 4.41 (2.60-7.47) | 4.38 | 36.47 | 1.83 | 1.08 | 3 | Yes |
| Muscle spasms | 14 | 24893 | 1.24 (0.73-2.11) | 1.24 | 0.66 | 0.29 | -0.46 | 0 | No |
| Therapeutic response changed | 13 | 2815 | 10.28 (5.94-17.76) | 10.19 | 107.36 | 2.62 | 1.84 | 3 | Yes |
| Therapeutic response unexpected | 12 | 5294 | 5.04 (2.85-8.89) | 5.00 | 38.40 | 1.93 | 1.13 | 3 | Yes |
| Liver injury | 12 | 6630 | 4.02 (2.28-7.10) | 3.99 | 26.94 | 1.70 | 0.89 | 3 | Yes |
| Alopecia | 12 | 25383 | 1.04 (0.59-1.84) | 1.04 | 0.02 | 0.06 | -0.75 | 0 | No |
| Feeling abnormal | 12 | 25891 | 1.02 (0.58-1.81) | 1.02 | 0.01 | 0.03 | -0.78 | 0 | No |
| Anxiety | 12 | 35453 | 0.74 (0.42-1.31) | 0.75 | 1.04 | -0.39 | -1.20 | 0 | No |
| Inappropriate schedule of product administration | 12 | 58843 | 0.45 (0.25-0.79) | 0.45 | 8.22 | -1.09 | -1.89 | 0 | No |
| Arthralgia | 12 | 72562 | 0.36 (0.20-0.63) | 0.36 | 13.57 | -1.38 | -2.19 | 0 | No |
| Hepatic fibrosis | 11 | 567 | 43.14 (23.70-78.52) | 42.81 | 440.73 | 3.25 | 2.40 | 3 | Yes |
| Cholelithiasis | 11 | 4270 | 5.72 (3.16-10.36) | 5.68 | 42.42 | 2.03 | 1.19 | 3 | Yes |
| Rash pruritic | 11 | 9118 | 2.68 (1.48-4.84) | 2.66 | 11.44 | 1.22 | 0.39 | 3 | Yes |
| Brain fog | 11 | 10556 | 2.31 (1.28-4.18) | 2.30 | 8.10 | 1.05 | 0.21 | 3 | Yes |
| Syncope | 11 | 12338 | 1.97 (1.09-3.58) | 1.97 | 5.25 | 0.86 | 0.03 | 2 | No |
| Platelet count decreased | 11 | 16042 | 1.52 (0.84-2.75) | 1.51 | 1.92 | 0.54 | -0.30 | 0 | No |
| Chest pain | 11 | 22214 | 1.09 (0.60-1.98) | 1.09 | 0.09 | 0.12 | -0.72 | 0 | No |
| Drug hypersensitivity | 11 | 23530 | 1.03 (0.57-1.87) | 1.03 | 0.01 | 0.04 | -0.80 | 0 | No |
| Blood pressure increased | 11 | 23535 | 1.03 (0.57-1.87) | 1.03 | 0.01 | 0.04 | -0.80 | 0 | No |
| Faeces discoloured | 10 | 2692 | 8.25 (4.42-15.38) | 8.20 | 63.02 | 2.31 | 1.43 | 3 | Yes |
| Depression | 10 | 24137 | 0.91 (0.49-1.70) | 0.91 | 0.08 | -0.12 | -0.99 | 0 | No |
| Hepatic fibrosis marker increased | 9 | 11 | 1817.01 (751.79-4391.55) | 1805.57 | 8927.75 | 3.31 | 2.20 | 3 | Yes |
| International normalised ratio increased | 9 | 1258 | 15.88 (8.23-30.66) | 15.79 | 123.83 | 2.67 | 1.75 | 3 | Yes |
| Pancreatitis | 9 | 6704 | 2.98 (1.54-5.73) | 2.96 | 11.71 | 1.31 | 0.39 | 3 | Yes |
| Dyspepsia | 9 | 15271 | 1.30 (0.68-2.51) | 1.30 | 0.63 | 0.34 | -0.58 | 0 | No |
| Tremor | 9 | 20215 | 0.98 (0.51-1.89) | 0.98 | 0.00 | -0.02 | -0.94 | 0 | No |
| Myalgia | 9 | 23790 | 0.83 (0.43-1.61) | 0.83 | 0.30 | -0.24 | -1.15 | 0 | No |
| Pain in extremity | 9 | 36968 | 0.53 (0.28-1.03) | 0.54 | 3.63 | -0.83 | -1.74 | 0 | No |
| Product dose omission issue | 9 | 114857 | 0.17 (0.09-0.32) | 0.17 | 36.95 | -2.41 | -3.32 | 0 | No |
| Off label use | 9 | 231211 | 0.08 (0.04-0.15) | 0.09 | 94.46 | -3.40 | -4.32 | 0 | No |
| Anal incontinence | 8 | 2177 | 8.15 (4.06-16.35) | 8.11 | 49.71 | 2.18 | 1.21 | 3 | Yes |
| Hepatotoxicity | 8 | 3674 | 4.83 (2.41-9.68) | 4.81 | 24.08 | 1.75 | 0.79 | 3 | Yes |
| Drug-induced liver injury | 8 | 8170 | 2.17 (1.08-4.34) | 2.16 | 5.00 | 0.94 | -0.03 | 2 | No |
| Discomfort | 8 | 12994 | 1.36 (0.68-2.73) | 1.36 | 0.76 | 0.38 | -0.58 | 0 | No |
| Palpitations | 8 | 16347 | 1.08 (0.54-2.17) | 1.08 | 0.05 | 0.10 | -0.87 | 0 | No |
| Adverse drug reaction | 8 | 18184 | 0.97 (0.48-1.95) | 0.97 | 0.01 | -0.04 | -1.00 | 0 | No |
| Erythema | 8 | 28951 | 0.61 (0.30-1.22) | 0.61 | 2.02 | -0.65 | -1.62 | 0 | No |
| Nasopharyngitis | 8 | 35234 | 0.50 (0.25-1.00) | 0.50 | 4.02 | -0.92 | -1.88 | 0 | No |
| Wrong technique in product usage process | 8 | 40434 | 0.43 (0.22-0.87) | 0.44 | 5.89 | -1.10 | -2.07 | 0 | No |
| Pyrexia | 8 | 51037 | 0.34 (0.17-0.69) | 0.35 | 10.06 | -1.42 | -2.39 | 0 | No |
| Hepatic enzyme decreased | 7 | 108 | 143.73 (66.80-309.26) | 143.03 | 927.23 | 2.93 | 1.87 | 3 | Yes |
| Transaminases increased | 7 | 2914 | 5.32 (2.53-11.19) | 5.30 | 24.39 | 1.78 | 0.76 | 3 | Yes |
| Hepatitis | 7 | 3712 | 4.18 (1.99-8.78) | 4.16 | 16.80 | 1.57 | 0.55 | 3 | Yes |
| Eructation | 7 | 4186 | 3.70 (1.76-7.79) | 3.69 | 13.72 | 1.46 | 0.44 | 3 | Yes |
| Laboratory test abnormal | 7 | 4299 | 3.61 (1.71-7.58) | 3.59 | 13.10 | 1.44 | 0.41 | 3 | Yes |
| Hypoglycaemia | 7 | 5590 | 2.77 (1.32-5.83) | 2.76 | 7.88 | 1.18 | 0.15 | 3 | Yes |
| Lethargy | 7 | 7009 | 2.21 (1.05-4.65) | 2.20 | 4.61 | 0.94 | -0.09 | 2 | No |
| Rash erythematous | 7 | 10265 | 1.51 (0.72-3.17) | 1.50 | 1.19 | 0.50 | -0.52 | 0 | No |
| Hyperhidrosis | 7 | 15335 | 1.01 (0.48-2.12) | 1.01 | 0.00 | 0.01 | -1.01 | 0 | No |
| Drug interaction | 7 | 20819 | 0.74 (0.35-1.56) | 0.74 | 0.63 | -0.38 | -1.41 | 0 | No |
| COVID-19 | 7 | 45795 | 0.33 (0.16-0.70) | 0.34 | 9.25 | -1.44 | -2.47 | 0 | No |
| Fall | 7 | 47843 | 0.32 (0.15-0.67) | 0.32 | 10.09 | -1.50 | -2.53 | 0 | No |
| Pneumonia | 7 | 54342 | 0.28 (0.13-0.59) | 0.28 | 12.83 | -1.68 | -2.70 | 0 | No |
| Unintentional use for unapproved indication | 6 | 165 | 80.58 (35.62-182.27) | 80.25 | 453.11 | 2.70 | 1.58 | 3 | Yes |
| Faeces pale | 6 | 280 | 47.48 (21.12-106.78) | 47.29 | 266.18 | 2.63 | 1.52 | 3 | Yes |
| Blood thyroid stimulating hormone decreased | 6 | 477 | 27.87 (12.44-62.46) | 27.76 | 152.87 | 2.52 | 1.42 | 3 | Yes |
| Splenomegaly | 6 | 1543 | 8.61 (3.86-19.23) | 8.58 | 40.05 | 2.04 | 0.94 | 3 | Yes |
| Abdominal pain lower | 6 | 3265 | 4.07 (1.82-9.08) | 4.06 | 13.80 | 1.50 | 0.40 | 3 | Yes |
| Ascites | 6 | 3934 | 3.38 (1.51-7.53) | 3.37 | 9.97 | 1.33 | 0.23 | 3 | Yes |
| Night sweats | 6 | 5773 | 2.30 (1.03-5.13) | 2.29 | 4.38 | 0.95 | -0.14 | 2 | No |
| Taste disorder | 6 | 6529 | 2.03 (0.91-4.53) | 2.03 | 3.13 | 0.82 | -0.27 | 0 | No |
| Nephrolithiasis | 6 | 8308 | 1.60 (0.72-3.56) | 1.59 | 1.33 | 0.55 | -0.54 | 0 | No |
| Blood pressure decreased | 6 | 9548 | 1.39 (0.62-3.10) | 1.39 | 0.65 | 0.39 | -0.70 | 0 | No |
| Gastrooesophageal reflux disease | 6 | 12097 | 1.09 (0.49-2.44) | 1.09 | 0.05 | 0.11 | -0.98 | 0 | No |
| Loss of personal independence in daily activities | 6 | 12516 | 1.06 (0.47-2.36) | 1.06 | 0.02 | 0.07 | -1.03 | 0 | No |
| Dry mouth | 6 | 12545 | 1.06 (0.47-2.35) | 1.06 | 0.02 | 0.07 | -1.03 | 0 | No |
| Heart rate increased | 6 | 15547 | 0.85 (0.38-1.90) | 0.85 | 0.16 | -0.20 | -1.30 | 0 | No |
| Chills | 6 | 16255 | 0.81 (0.36-1.81) | 0.81 | 0.25 | -0.26 | -1.35 | 0 | No |
| Migraine | 6 | 16349 | 0.81 (0.36-1.80) | 0.81 | 0.27 | -0.27 | -1.36 | 0 | No |
| Maternal exposure during pregnancy | 6 | 19930 | 0.66 (0.30-1.48) | 0.66 | 1.02 | -0.52 | -1.61 | 0 | No |
| Gastrointestinal disorder | 6 | 21088 | 0.63 (0.28-1.40) | 0.63 | 1.33 | -0.59 | -1.69 | 0 | No |
| Cough | 6 | 49386 | 0.27 (0.12-0.59) | 0.27 | 12.18 | -1.74 | -2.83 | 0 | No |
| Hepatomegaly | 5 | 937 | 11.81 (4.90-28.49) | 11.78 | 49.06 | 2.07 | 0.89 | 3 | Yes |
| Cholecystitis | 5 | 1729 | 6.40 (2.66-15.42) | 6.38 | 22.64 | 1.75 | 0.57 | 3 | Yes |
| Faeces soft | 5 | 2221 | 4.98 (2.07-12.00) | 4.97 | 15.82 | 1.58 | 0.40 | 3 | Yes |
| Musculoskeletal chest pain | 5 | 2366 | 4.68 (1.94-11.26) | 4.66 | 14.37 | 1.53 | 0.35 | 3 | Yes |
| Thirst | 5 | 2643 | 4.19 (1.74-10.08) | 4.17 | 12.06 | 1.45 | 0.27 | 3 | Yes |
| Retching | 5 | 3192 | 3.47 (1.44-8.34) | 3.46 | 8.73 | 1.29 | 0.11 | 3 | Yes |
| Nightmare | 5 | 3956 | 2.80 (1.16-6.73) | 2.79 | 5.74 | 1.10 | -0.08 | 2 | No |
| Pollakiuria | 5 | 5950 | 1.86 (0.77-4.47) | 1.85 | 1.97 | 0.70 | -0.48 | 0 | No |
| Therapy cessation | 5 | 8439 | 1.31 (0.54-3.15) | 1.31 | 0.36 | 0.31 | -0.87 | 0 | No |
| Renal failure | 5 | 11884 | 0.93 (0.39-2.23) | 0.93 | 0.03 | -0.09 | -1.27 | 0 | No |
| Oedema peripheral | 5 | 12085 | 0.91 (0.38-2.20) | 0.91 | 0.04 | -0.11 | -1.29 | 0 | No |
| Contusion | 5 | 14844 | 0.74 (0.31-1.79) | 0.74 | 0.45 | -0.37 | -1.55 | 0 | No |
| Muscular weakness | 5 | 16251 | 0.68 (0.28-1.63) | 0.68 | 0.76 | -0.48 | -1.66 | 0 | No |
| Loss of consciousness | 5 | 16408 | 0.67 (0.28-1.62) | 0.67 | 0.80 | -0.49 | -1.67 | 0 | No |
| Gait disturbance | 5 | 25169 | 0.44 (0.18-1.05) | 0.44 | 3.63 | -1.05 | -2.23 | 0 | No |
| Urinary tract infection | 5 | 29022 | 0.38 (0.16-0.91) | 0.38 | 5.10 | -1.24 | -2.42 | 0 | No |
| Aspartate aminotransferase decreased | 4 | 91 | 97.27 (35.69-265.12) | 97.00 | 364.06 | 2.26 | 0.93 | 3 | Yes |
| Suspected drug-induced liver injury | 4 | 133 | 66.55 (24.58-180.19) | 66.37 | 250.03 | 2.23 | 0.92 | 3 | Yes |
| Alanine aminotransferase decreased | 4 | 139 | 63.68 (23.54-172.30) | 63.51 | 239.21 | 2.23 | 0.92 | 3 | Yes |
| Intentional underdose | 4 | 761 | 11.63 (4.35-31.11) | 11.60 | 38.55 | 1.89 | 0.60 | 3 | Yes |
| Hepatic enzyme abnormal | 4 | 781 | 11.33 (4.24-30.31) | 11.30 | 37.38 | 1.88 | 0.59 | 3 | Yes |
| Serum ferritin increased | 4 | 829 | 10.68 (3.99-28.55) | 10.65 | 34.81 | 1.86 | 0.56 | 3 | Yes |
| Autoimmune hepatitis | 4 | 1210 | 7.31 (2.74-19.54) | 7.30 | 21.67 | 1.69 | 0.39 | 3 | Yes |
| Urosepsis | 4 | 1375 | 6.44 (2.41-17.19) | 6.42 | 18.26 | 1.62 | 0.33 | 3 | Yes |
| Abnormal faeces | 4 | 1440 | 6.14 (2.30-16.42) | 6.13 | 17.13 | 1.60 | 0.30 | 3 | Yes |
| Gastrointestinal pain | 4 | 1981 | 4.47 (1.67-11.93) | 4.46 | 10.71 | 1.40 | 0.10 | 3 | Yes |
| Gamma-glutamyltransferase increased | 4 | 2234 | 3.96 (1.48-10.57) | 3.95 | 8.81 | 1.31 | 0.02 | 3 | Yes |
| Cold sweat | 4 | 2246 | 3.94 (1.47-10.52) | 3.93 | 8.73 | 1.31 | 0.01 | 3 | Yes |
| Gout | 4 | 2601 | 3.40 (1.27-9.08) | 3.39 | 6.75 | 1.20 | -0.10 | 2 | No |
| Diverticulitis | 4 | 4312 | 2.05 (0.77-5.47) | 2.05 | 2.14 | 0.76 | -0.54 | 0 | No |
| Blood glucose decreased | 4 | 4986 | 1.77 (0.66-4.73) | 1.77 | 1.34 | 0.62 | -0.68 | 0 | No |
| Rhabdomyolysis | 4 | 5623 | 1.57 (0.59-4.19) | 1.57 | 0.83 | 0.49 | -0.80 | 0 | No |
| Rash macular | 4 | 6179 | 1.43 (0.54-3.82) | 1.43 | 0.51 | 0.39 | -0.90 | 0 | No |
| Frequent bowel movements | 4 | 6709 | 1.32 (0.49-3.51) | 1.32 | 0.30 | 0.31 | -0.99 | 0 | No |
| Dysgeusia | 4 | 7200 | 1.23 (0.46-3.27) | 1.23 | 0.17 | 0.23 | -1.06 | 0 | No |
| Colitis | 4 | 7761 | 1.14 (0.43-3.04) | 1.14 | 0.07 | 0.15 | -1.15 | 0 | No |
| Feeling hot | 4 | 7829 | 1.13 (0.42-3.01) | 1.13 | 0.06 | 0.14 | -1.16 | 0 | No |
| Diabetes mellitus | 4 | 8847 | 1.00 (0.37-2.66) | 1.00 | 0.00 | -0.00 | -1.30 | 0 | No |
| Bronchitis | 4 | 9928 | 0.89 (0.33-2.37) | 0.89 | 0.06 | -0.14 | -1.43 | 0 | No |
| Eye pain | 4 | 11165 | 0.79 (0.30-2.11) | 0.79 | 0.22 | -0.28 | -1.57 | 0 | No |
| Arthritis | 4 | 12142 | 0.73 (0.27-1.94) | 0.73 | 0.41 | -0.38 | -1.67 | 0 | No |
| Musculoskeletal stiffness | 4 | 12883 | 0.68 (0.26-1.83) | 0.69 | 0.58 | -0.45 | -1.75 | 0 | No |
| Dysphagia | 4 | 13974 | 0.63 (0.24-1.68) | 0.63 | 0.86 | -0.55 | -1.85 | 0 | No |
| Oropharyngeal pain | 4 | 15236 | 0.58 (0.22-1.54) | 0.58 | 1.23 | -0.66 | -1.95 | 0 | No |
| Dehydration | 4 | 16843 | 0.52 (0.20-1.39) | 0.52 | 1.74 | -0.79 | -2.08 | 0 | No |
| Vision blurred | 4 | 20308 | 0.43 (0.16-1.16) | 0.43 | 2.96 | -1.03 | -2.32 | 0 | No |
| Confusional state | 4 | 20808 | 0.42 (0.16-1.13) | 0.42 | 3.15 | -1.06 | -2.35 | 0 | No |
| Visual impairment | 4 | 21010 | 0.42 (0.16-1.12) | 0.42 | 3.22 | -1.07 | -2.37 | 0 | No |
| Influenza | 4 | 24947 | 0.35 (0.13-0.94) | 0.35 | 4.76 | -1.30 | -2.59 | 0 | No |
| Anaemia | 4 | 25352 | 0.35 (0.13-0.92) | 0.35 | 4.92 | -1.32 | -2.61 | 0 | No |
| Hypotension | 4 | 26978 | 0.33 (0.12-0.87) | 0.33 | 5.58 | -1.40 | -2.70 | 0 | No |
| Peripheral swelling | 4 | 27424 | 0.32 (0.12-0.85) | 0.32 | 5.76 | -1.43 | -2.72 | 0 | No |
| Product use issue | 4 | 34827 | 0.25 (0.09-0.67) | 0.25 | 8.89 | -1.75 | -3.04 | 0 | No |
| Hepatitis alcoholic | 3 | 34 | 195.13 (59.86-636.04) | 194.72 | 531.30 | 1.98 | 0.46 | 3 | Yes |
| Thyroxine decreased | 3 | 97 | 68.39 (21.65-216.03) | 68.25 | 192.84 | 1.94 | 0.46 | 3 | Yes |
| Blood cholesterol decreased | 3 | 281 | 23.61 (7.56-73.73) | 23.56 | 64.13 | 1.82 | 0.37 | 3 | Yes |
| Blood testosterone increased | 3 | 291 | 22.80 (7.30-71.18) | 22.75 | 61.75 | 1.82 | 0.36 | 3 | Yes |
| High density lipoprotein decreased | 3 | 339 | 19.57 (6.27-61.05) | 19.53 | 52.28 | 1.79 | 0.34 | 3 | Yes |
| Hepatic cyst | 3 | 475 | 13.96 (4.48-43.51) | 13.94 | 35.81 | 1.72 | 0.27 | 3 | Yes |
| Lipase increased | 3 | 990 | 6.70 (2.15-20.83) | 6.69 | 14.47 | 1.46 | 0.02 | 3 | Yes |
| Flank pain | 3 | 1353 | 4.90 (1.58-15.23) | 4.89 | 9.28 | 1.31 | -0.14 | 2 | No |
| Hyperbilirubinaemia | 3 | 1694 | 3.91 (1.26-12.16) | 3.91 | 6.48 | 1.18 | -0.27 | 2 | No |
| Blood triglycerides increased | 3 | 1999 | 3.32 (1.07-10.30) | 3.31 | 4.84 | 1.07 | -0.38 | 2 | No |
| Liver function test abnormal | 3 | 2124 | 3.12 (1.00-9.70) | 3.12 | 4.31 | 1.03 | -0.42 | 2 | No |
| Ulcer | 3 | 2253 | 2.94 (0.95-9.14) | 2.94 | 3.83 | 0.98 | -0.46 | 0 | No |
| Rash papular | 3 | 2352 | 2.82 (0.91-8.76) | 2.81 | 3.51 | 0.95 | -0.49 | 0 | No |
| Head discomfort | 3 | 3220 | 2.06 (0.66-6.39) | 2.06 | 1.63 | 0.70 | -0.75 | 0 | No |
| Pancreatitis acute | 3 | 3460 | 1.92 (0.62-5.95) | 1.91 | 1.31 | 0.64 | -0.81 | 0 | No |
| Proteinuria | 3 | 3604 | 1.84 (0.59-5.71) | 1.84 | 1.14 | 0.60 | -0.84 | 0 | No |
| Musculoskeletal pain | 3 | 3687 | 1.80 (0.58-5.58) | 1.80 | 1.06 | 0.58 | -0.86 | 0 | No |
| Feeling cold | 3 | 3816 | 1.74 (0.56-5.39) | 1.73 | 0.93 | 0.55 | -0.90 | 0 | No |
| Poor quality sleep | 3 | 3828 | 1.73 (0.56-5.38) | 1.73 | 0.92 | 0.55 | -0.90 | 0 | No |
| Blood potassium decreased | 3 | 4076 | 1.63 (0.52-5.05) | 1.62 | 0.72 | 0.49 | -0.96 | 0 | No |
| Glycosylated haemoglobin increased | 3 | 4147 | 1.60 (0.51-4.96) | 1.60 | 0.67 | 0.47 | -0.97 | 0 | No |
| Lip swelling | 3 | 4336 | 1.53 (0.49-4.75) | 1.53 | 0.55 | 0.43 | -1.01 | 0 | No |
| Hypersomnia | 3 | 4340 | 1.53 (0.49-4.74) | 1.53 | 0.54 | 0.43 | -1.02 | 0 | No |
| Hyperglycaemia | 3 | 4461 | 1.49 (0.48-4.61) | 1.48 | 0.47 | 0.40 | -1.04 | 0 | No |
| White blood cell count increased | 3 | 4622 | 1.43 (0.46-4.45) | 1.43 | 0.39 | 0.37 | -1.08 | 0 | No |
| Eye swelling | 3 | 5614 | 1.18 (0.38-3.66) | 1.18 | 0.08 | 0.17 | -1.27 | 0 | No |
| Multiple organ dysfunction syndrome | 3 | 6785 | 0.98 (0.31-3.03) | 0.98 | 0.00 | -0.03 | -1.47 | 0 | No |
| Septic shock | 3 | 7452 | 0.89 (0.29-2.76) | 0.89 | 0.04 | -0.13 | -1.58 | 0 | No |
| Swelling face | 3 | 8887 | 0.74 (0.24-2.31) | 0.74 | 0.26 | -0.33 | -1.78 | 0 | No |
| Influenza like illness | 3 | 9250 | 0.72 (0.23-2.22) | 0.72 | 0.34 | -0.38 | -1.82 | 0 | No |
| Blister | 3 | 9482 | 0.70 (0.22-2.17) | 0.70 | 0.39 | -0.41 | -1.85 | 0 | No |
| Product prescribing issue | 3 | 9531 | 0.69 (0.22-2.15) | 0.69 | 0.40 | -0.41 | -1.86 | 0 | No |
| Epistaxis | 3 | 9980 | 0.66 (0.21-2.06) | 0.66 | 0.51 | -0.47 | -1.91 | 0 | No |
| Flushing | 3 | 10062 | 0.66 (0.21-2.04) | 0.66 | 0.53 | -0.48 | -1.92 | 0 | No |
| Stomatitis | 3 | 10124 | 0.65 (0.21-2.03) | 0.65 | 0.55 | -0.48 | -1.93 | 0 | No |
| Inflammation | 3 | 10212 | 0.65 (0.21-2.01) | 0.65 | 0.57 | -0.49 | -1.94 | 0 | No |
| Balance disorder | 3 | 11006 | 0.60 (0.19-1.87) | 0.60 | 0.79 | -0.58 | -2.03 | 0 | No |
| Therapy non-responder | 3 | 11126 | 0.59 (0.19-1.84) | 0.60 | 0.83 | -0.60 | -2.04 | 0 | No |
| Intentional dose omission | 3 | 12643 | 0.52 (0.17-1.62) | 0.52 | 1.30 | -0.75 | -2.20 | 0 | No |
| Intentional product misuse | 3 | 12772 | 0.52 (0.17-1.61) | 0.52 | 1.35 | -0.76 | -2.21 | 0 | No |
| Haemoglobin decreased | 3 | 13353 | 0.49 (0.16-1.54) | 0.50 | 1.54 | -0.82 | -2.26 | 0 | No |
| Product dose omission in error | 3 | 14258 | 0.46 (0.15-1.44) | 0.46 | 1.86 | -0.90 | -2.34 | 0 | No |
| Sinusitis | 3 | 17090 | 0.39 (0.12-1.20) | 0.39 | 2.92 | -1.13 | -2.57 | 0 | No |
| Therapeutic product effect incomplete | 3 | 17119 | 0.39 (0.12-1.20) | 0.39 | 2.93 | -1.13 | -2.58 | 0 | No |
| Eczema | 3 | 20798 | 0.32 (0.10-0.98) | 0.32 | 4.41 | -1.38 | -2.83 | 0 | No |
| Dry skin | 3 | 26434 | 0.25 (0.08-0.77) | 0.25 | 6.79 | -1.70 | -3.14 | 0 | No |
| Perihepatic discomfort | 2 | 13 | 339.98 (76.65-1507.98) | 339.51 | 585.02 | 1.57 | -0.24 | 0 | No |
| Liver tenderness | 2 | 17 | 259.99 (60.01-1126.35) | 259.62 | 461.02 | 1.57 | -0.22 | 0 | No |
| Liver scan abnormal | 2 | 18 | 245.54 (56.92-1059.23) | 245.20 | 437.77 | 1.57 | -0.21 | 0 | No |
| Alcoholic liver disease | 2 | 29 | 152.41 (36.33-639.32) | 152.19 | 281.02 | 1.56 | -0.18 | 0 | No |
| Alpha-1 antitrypsin deficiency | 2 | 42 | 105.23 (25.45-435.14) | 105.09 | 196.82 | 1.56 | -0.17 | 0 | No |
| Alcohol use disorder | 2 | 51 | 86.66 (21.08-356.31) | 86.54 | 162.73 | 1.55 | -0.16 | 0 | No |
| Pancreatic duct dilatation | 2 | 54 | 81.85 (19.94-336.02) | 81.73 | 153.80 | 1.55 | -0.16 | 0 | No |
| Tri-iodothyronine decreased | 2 | 68 | 65.00 (15.91-265.45) | 64.91 | 122.25 | 1.54 | -0.16 | 0 | No |
| Gastric varices | 2 | 77 | 57.40 (14.09-233.88) | 57.32 | 107.88 | 1.53 | -0.16 | 0 | No |
| Thyroxine free decreased | 2 | 81 | 54.56 (13.40-222.13) | 54.49 | 102.49 | 1.53 | -0.17 | 0 | No |
| Liver function test decreased | 2 | 88 | 50.22 (12.35-204.19) | 50.15 | 94.21 | 1.53 | -0.17 | 0 | No |
| Blister rupture | 2 | 137 | 32.26 (7.98-130.43) | 32.22 | 59.63 | 1.50 | -0.19 | 0 | No |
| Primary biliary cholangitis | 2 | 138 | 32.03 (7.92-129.47) | 31.98 | 59.17 | 1.50 | -0.19 | 0 | No |
| Tendon discomfort | 2 | 138 | 32.03 (7.92-129.47) | 31.98 | 59.17 | 1.50 | -0.19 | 0 | No |
| Metabolic dysfunction-associated liver disease | 2 | 150 | 29.46 (7.29-119.02) | 29.42 | 54.20 | 1.49 | -0.20 | 0 | No |
| Coccidioidomycosis | 2 | 163 | 27.11 (6.72-109.44) | 27.08 | 49.62 | 1.48 | -0.20 | 0 | No |
| Anal pruritus | 2 | 188 | 23.51 (5.83-94.78) | 23.48 | 42.59 | 1.47 | -0.22 | 0 | No |
| Blood thyroid stimulating hormone abnormal | 2 | 231 | 19.13 (4.75-77.03) | 19.11 | 34.03 | 1.44 | -0.24 | 0 | No |
| Steatorrhoea | 2 | 272 | 16.25 (4.04-65.36) | 16.23 | 28.37 | 1.42 | -0.26 | 0 | No |
| Early satiety | 2 | 274 | 16.13 (4.01-64.88) | 16.11 | 28.14 | 1.41 | -0.26 | 0 | No |
| Obstructive pancreatitis | 2 | 279 | 15.84 (3.94-63.71) | 15.82 | 27.57 | 1.41 | -0.27 | 0 | No |
| Oesophageal varices haemorrhage | 2 | 280 | 15.78 (3.92-63.49) | 15.76 | 27.46 | 1.41 | -0.27 | 0 | No |
| Thyroid function test abnormal | 2 | 334 | 13.23 (3.29-53.18) | 13.21 | 22.45 | 1.38 | -0.30 | 0 | No |
| Ophthalmic migraine | 2 | 344 | 12.85 (3.20-51.63) | 12.83 | 21.69 | 1.37 | -0.30 | 0 | No |
| Portal vein thrombosis | 2 | 462 | 9.57 (2.38-38.40) | 9.55 | 15.25 | 1.31 | -0.36 | 0 | No |
| Anorectal discomfort | 2 | 481 | 9.19 (2.29-36.88) | 9.18 | 14.51 | 1.30 | -0.37 | 0 | No |
| Ammonia increased | 2 | 486 | 9.09 (2.27-36.50) | 9.08 | 14.33 | 1.30 | -0.38 | 0 | No |
| Epigastric discomfort | 2 | 498 | 8.87 (2.21-35.62) | 8.86 | 13.90 | 1.29 | -0.38 | 0 | No |
| Vomiting projectile | 2 | 586 | 7.54 (1.88-30.25) | 7.53 | 11.29 | 1.24 | -0.43 | 0 | No |
| Tongue disorder | 2 | 631 | 7.00 (1.75-28.09) | 6.99 | 10.24 | 1.22 | -0.45 | 0 | No |
| Hepatitis B | 2 | 675 | 6.55 (1.63-26.26) | 6.54 | 9.36 | 1.20 | -0.47 | 0 | No |
| Hepatic lesion | 2 | 684 | 6.46 (1.61-25.91) | 6.45 | 9.19 | 1.19 | -0.48 | 0 | No |
| Cholecystitis acute | 2 | 701 | 6.30 (1.57-25.28) | 6.30 | 8.89 | 1.19 | -0.49 | 0 | No |
| Urine odour abnormal | 2 | 764 | 5.78 (1.44-23.19) | 5.78 | 7.88 | 1.15 | -0.52 | 0 | No |
| Tendon disorder | 2 | 812 | 5.44 (1.36-21.82) | 5.44 | 7.22 | 1.13 | -0.54 | 0 | No |
| Haemorrhagic stroke | 2 | 830 | 5.32 (1.33-21.34) | 5.32 | 7.00 | 1.12 | -0.55 | 0 | No |
| Gastrointestinal sounds abnormal | 2 | 957 | 4.62 (1.15-18.51) | 4.61 | 5.65 | 1.06 | -0.61 | 0 | No |
| Hepatic cancer | 2 | 969 | 4.56 (1.14-18.28) | 4.55 | 5.54 | 1.06 | -0.61 | 0 | No |
| Skin abrasion | 2 | 975 | 4.53 (1.13-18.16) | 4.53 | 5.49 | 1.06 | -0.61 | 0 | No |
| Heart rate abnormal | 2 | 988 | 4.47 (1.12-17.92) | 4.47 | 5.37 | 1.05 | -0.62 | 0 | No |
| Blindness transient | 2 | 1032 | 4.28 (1.07-17.16) | 4.28 | 5.01 | 1.03 | -0.64 | 0 | No |
| Blood albumin decreased | 2 | 1035 | 4.27 (1.07-17.11) | 4.26 | 4.99 | 1.03 | -0.64 | 0 | No |
| Platelet count abnormal | 2 | 1133 | 3.90 (0.97-15.63) | 3.90 | 4.30 | 0.99 | -0.68 | 0 | No |
| Hepatic encephalopathy | 2 | 1156 | 3.82 (0.95-15.32) | 3.82 | 4.15 | 0.98 | -0.69 | 0 | No |
| Blood glucose fluctuation | 2 | 1165 | 3.79 (0.95-15.20) | 3.79 | 4.10 | 0.97 | -0.70 | 0 | No |
| Ventricular fibrillation | 2 | 1263 | 3.50 (0.87-14.02) | 3.49 | 3.56 | 0.93 | -0.74 | 0 | No |
| Cholangitis | 2 | 1268 | 3.48 (0.87-13.96) | 3.48 | 3.53 | 0.93 | -0.74 | 0 | No |
| Papule | 2 | 1350 | 3.27 (0.82-13.11) | 3.27 | 3.15 | 0.89 | -0.77 | 0 | No |
| Abnormal uterine bleeding | 2 | 1478 | 2.99 (0.75-11.97) | 2.99 | 2.64 | 0.84 | -0.83 | 0 | No |
| Vitamin D deficiency | 2 | 1499 | 2.95 (0.74-11.81) | 2.94 | 2.57 | 0.84 | -0.83 | 0 | No |
| Blood magnesium decreased | 2 | 1515 | 2.92 (0.73-11.68) | 2.91 | 2.51 | 0.83 | -0.84 | 0 | No |
| Renal pain | 2 | 1529 | 2.89 (0.72-11.57) | 2.89 | 2.46 | 0.82 | -0.85 | 0 | No |
| Cholecystectomy | 2 | 1739 | 2.54 (0.63-10.17) | 2.54 | 1.86 | 0.75 | -0.92 | 0 | No |
| Tendonitis | 2 | 1794 | 2.46 (0.61-9.86) | 2.46 | 1.73 | 0.73 | -0.94 | 0 | No |
| Generalised oedema | 2 | 1975 | 2.24 (0.56-8.96) | 2.23 | 1.36 | 0.66 | -1.01 | 0 | No |
| Dizziness postural | 2 | 1992 | 2.22 (0.55-8.88) | 2.22 | 1.33 | 0.66 | -1.01 | 0 | No |
| Carpal tunnel syndrome | 2 | 2048 | 2.16 (0.54-8.64) | 2.16 | 1.24 | 0.64 | -1.03 | 0 | No |
| Blood potassium increased | 2 | 2055 | 2.15 (0.54-8.61) | 2.15 | 1.23 | 0.63 | -1.03 | 0 | No |
| Neurodermatitis | 2 | 2168 | 2.04 (0.51-8.16) | 2.04 | 1.05 | 0.60 | -1.07 | 0 | No |
| Sleep disorder due to general medical condition, insomnia type | 2 | 2291 | 1.93 (0.48-7.72) | 1.93 | 0.89 | 0.56 | -1.11 | 0 | No |
| Haemorrhoids | 2 | 2694 | 1.64 (0.41-6.56) | 1.64 | 0.50 | 0.43 | -1.24 | 0 | No |
| Autoimmune disorder | 2 | 2798 | 1.58 (0.39-6.32) | 1.58 | 0.42 | 0.40 | -1.27 | 0 | No |
| Haematemesis | 2 | 2855 | 1.55 (0.39-6.19) | 1.55 | 0.39 | 0.39 | -1.28 | 0 | No |
| Hepatic steatosis | 2 | 2882 | 1.53 (0.38-6.14) | 1.53 | 0.37 | 0.38 | -1.29 | 0 | No |
| Bursitis | 2 | 2983 | 1.48 (0.37-5.93) | 1.48 | 0.31 | 0.35 | -1.32 | 0 | No |
| Ear pain | 2 | 3097 | 1.43 (0.36-5.71) | 1.43 | 0.25 | 0.32 | -1.35 | 0 | No |
| Blood creatine phosphokinase increased | 2 | 3109 | 1.42 (0.35-5.69) | 1.42 | 0.25 | 0.32 | -1.35 | 0 | No |
| Pallor | 2 | 3225 | 1.37 (0.34-5.48) | 1.37 | 0.20 | 0.28 | -1.38 | 0 | No |
| Hunger | 2 | 3450 | 1.28 (0.32-5.12) | 1.28 | 0.12 | 0.23 | -1.44 | 0 | No |
| Prescribed underdose | 2 | 3462 | 1.28 (0.32-5.11) | 1.27 | 0.12 | 0.22 | -1.45 | 0 | No |
| Ageusia | 2 | 3483 | 1.27 (0.32-5.08) | 1.27 | 0.11 | 0.22 | -1.45 | 0 | No |
| Haemoptysis | 2 | 3531 | 1.25 (0.31-5.01) | 1.25 | 0.10 | 0.21 | -1.46 | 0 | No |
| Swollen tongue | 2 | 3549 | 1.24 (0.31-4.98) | 1.24 | 0.10 | 0.20 | -1.47 | 0 | No |
| Presyncope | 2 | 3593 | 1.23 (0.31-4.92) | 1.23 | 0.08 | 0.19 | -1.48 | 0 | No |
| Rash maculo-papular | 2 | 3667 | 1.20 (0.30-4.82) | 1.20 | 0.07 | 0.17 | -1.50 | 0 | No |
| Skin haemorrhage | 2 | 3866 | 1.14 (0.29-4.57) | 1.14 | 0.04 | 0.12 | -1.54 | 0 | No |
| Heavy menstrual bleeding | 2 | 3872 | 1.14 (0.28-4.56) | 1.14 | 0.03 | 0.12 | -1.55 | 0 | No |
| Urinary incontinence | 2 | 3954 | 1.12 (0.28-4.47) | 1.12 | 0.02 | 0.10 | -1.57 | 0 | No |
| Hypophagia | 2 | 4043 | 1.09 (0.27-4.37) | 1.09 | 0.02 | 0.08 | -1.59 | 0 | No |
| Disorientation | 2 | 4091 | 1.08 (0.27-4.32) | 1.08 | 0.01 | 0.07 | -1.60 | 0 | No |
| Product distribution issue | 2 | 4613 | 0.96 (0.24-3.83) | 0.96 | 0.00 | -0.04 | -1.71 | 0 | No |
| Nervousness | 2 | 4799 | 0.92 (0.23-3.68) | 0.92 | 0.01 | -0.08 | -1.75 | 0 | No |
| Panic attack | 2 | 4932 | 0.89 (0.22-3.58) | 0.89 | 0.02 | -0.11 | -1.78 | 0 | No |
| Lymphadenopathy | 2 | 5160 | 0.86 (0.21-3.42) | 0.86 | 0.05 | -0.15 | -1.82 | 0 | No |
| Lacrimation increased | 2 | 5890 | 0.75 (0.19-3.00) | 0.75 | 0.17 | -0.29 | -1.96 | 0 | No |
| Viral infection | 2 | 6533 | 0.68 (0.17-2.70) | 0.68 | 0.31 | -0.40 | -2.07 | 0 | No |
| Accidental overdose | 2 | 6830 | 0.65 (0.16-2.58) | 0.65 | 0.39 | -0.45 | -2.12 | 0 | No |
| Osteoarthritis | 2 | 6991 | 0.63 (0.16-2.53) | 0.63 | 0.43 | -0.48 | -2.14 | 0 | No |
| Insurance issue | 2 | 7275 | 0.61 (0.15-2.43) | 0.61 | 0.51 | -0.52 | -2.19 | 0 | No |
| Vertigo | 2 | 7871 | 0.56 (0.14-2.24) | 0.56 | 0.69 | -0.61 | -2.27 | 0 | No |
| Blood creatinine increased | 2 | 8129 | 0.54 (0.14-2.17) | 0.54 | 0.77 | -0.64 | -2.31 | 0 | No |
| Lung disorder | 2 | 8406 | 0.52 (0.13-2.10) | 0.53 | 0.86 | -0.68 | -2.35 | 0 | No |
| Burning sensation | 2 | 8770 | 0.50 (0.13-2.01) | 0.50 | 0.98 | -0.73 | -2.40 | 0 | No |
| Product availability issue | 2 | 8906 | 0.49 (0.12-1.98) | 0.50 | 1.03 | -0.75 | -2.42 | 0 | No |
| Ocular hyperaemia | 2 | 9477 | 0.46 (0.12-1.86) | 0.47 | 1.23 | -0.82 | -2.49 | 0 | No |
| Thrombosis | 2 | 10043 | 0.44 (0.11-1.76) | 0.44 | 1.43 | -0.89 | -2.56 | 0 | No |
| Therapeutic product effect decreased | 2 | 10345 | 0.43 (0.11-1.70) | 0.43 | 1.55 | -0.92 | -2.59 | 0 | No |
| Haematochezia | 2 | 10499 | 0.42 (0.10-1.68) | 0.42 | 1.60 | -0.94 | -2.61 | 0 | No |
| Myocardial infarction | 2 | 11383 | 0.39 (0.10-1.55) | 0.39 | 1.94 | -1.04 | -2.71 | 0 | No |
| Nasal congestion | 2 | 11500 | 0.38 (0.10-1.53) | 0.38 | 1.99 | -1.05 | -2.72 | 0 | No |
| Tachycardia | 2 | 12536 | 0.35 (0.09-1.41) | 0.35 | 2.39 | -1.16 | -2.82 | 0 | No |
| Stress | 2 | 12680 | 0.35 (0.09-1.39) | 0.35 | 2.45 | -1.17 | -2.84 | 0 | No |
| Wheezing | 2 | 12948 | 0.34 (0.08-1.36) | 0.34 | 2.56 | -1.20 | -2.86 | 0 | No |
| Atrial fibrillation | 2 | 13171 | 0.33 (0.08-1.34) | 0.34 | 2.65 | -1.22 | -2.88 | 0 | No |
| Swelling | 2 | 14617 | 0.30 (0.08-1.20) | 0.30 | 3.24 | -1.35 | -3.01 | 0 | No |
| Treatment failure | 2 | 15634 | 0.28 (0.07-1.13) | 0.28 | 3.67 | -1.43 | -3.10 | 0 | No |
| Hot flush | 2 | 16216 | 0.27 (0.07-1.09) | 0.27 | 3.91 | -1.48 | -3.14 | 0 | No |
| Psoriasis | 2 | 16648 | 0.26 (0.07-1.06) | 0.27 | 4.10 | -1.51 | -3.18 | 0 | No |
| Drug intolerance | 2 | 16850 | 0.26 (0.07-1.04) | 0.26 | 4.18 | -1.53 | -3.19 | 0 | No |
| Neuropathy peripheral | 2 | 17507 | 0.25 (0.06-1.00) | 0.25 | 4.46 | -1.57 | -3.24 | 0 | No |
| Paraesthesia | 2 | 18328 | 0.24 (0.06-0.96) | 0.24 | 4.81 | -1.63 | -3.30 | 0 | No |
| Memory impairment | 2 | 19133 | 0.23 (0.06-0.92) | 0.23 | 5.16 | -1.69 | -3.36 | 0 | No |
| Hypoaesthesia | 2 | 20299 | 0.22 (0.05-0.87) | 0.22 | 5.67 | -1.77 | -3.43 | 0 | No |
| Seizure | 2 | 23479 | 0.19 (0.05-0.75) | 0.19 | 7.07 | -1.96 | -3.62 | 0 | No |
| General physical health deterioration | 2 | 24257 | 0.18 (0.05-0.72) | 0.18 | 7.41 | -2.00 | -3.67 | 0 | No |
| Acute kidney injury | 2 | 26562 | 0.16 (0.04-0.66) | 0.17 | 8.44 | -2.12 | -3.79 | 0 | No |
| Dermatitis atopic | 2 | 29180 | 0.15 (0.04-0.60) | 0.15 | 9.61 | -2.25 | -3.91 | 0 | No |
| Hypertension | 2 | 32133 | 0.14 (0.03-0.54) | 0.14 | 10.95 | -2.38 | -4.04 | 0 | No |
| Injection site pain | 2 | 62115 | 0.07 (0.02-0.28) | 0.07 | 24.78 | -3.28 | -4.95 | 0 | No |
| Hepatic fibrosis marker abnormal | 1 | 1 | 2208.36 (138.06-35325.06) | 2206.81 | 1102.41 | 1.00 | -1.64 | 0 | No |
| Bladder calculus removal | 1 | 2 | 1104.18 (100.06-12184.46) | 1103.41 | 734.27 | 1.00 | -1.50 | 0 | No |
| Gastric pacemaker insertion | 1 | 3 | 736.12 (76.52-7081.20) | 735.60 | 550.20 | 1.00 | -1.42 | 0 | No |
| Bone densitometry | 1 | 5 | 441.67 (51.57-3782.97) | 441.36 | 366.14 | 1.00 | -1.32 | 0 | No |
| Acrochordon excision | 1 | 6 | 368.06 (44.28-3059.24) | 367.80 | 313.55 | 0.99 | -1.29 | 0 | No |
| Urine bilirubin increased | 1 | 7 | 315.48 (38.79-2565.89) | 315.26 | 274.10 | 0.99 | -1.26 | 0 | No |
| Creatinine urine abnormal | 1 | 11 | 200.76 (25.90-1556.07) | 200.62 | 182.07 | 0.99 | -1.20 | 0 | No |
| Gallbladder mass | 1 | 11 | 200.76 (25.90-1556.07) | 200.62 | 182.07 | 0.99 | -1.20 | 0 | No |
| Malignant neoplasm of ampulla of Vater | 1 | 11 | 200.76 (25.90-1556.07) | 200.62 | 182.07 | 0.99 | -1.20 | 0 | No |
| Smooth muscle antibody positive | 1 | 11 | 200.76 (25.90-1556.07) | 200.62 | 182.07 | 0.99 | -1.20 | 0 | No |
| Retinal migraine | 1 | 13 | 169.87 (22.21-1299.46) | 169.75 | 155.78 | 0.99 | -1.18 | 0 | No |
| Malignant urinary tract neoplasm | 1 | 16 | 138.02 (18.29-1041.48) | 137.93 | 127.94 | 0.99 | -1.16 | 0 | No |
| Eye naevus | 1 | 18 | 122.69 (16.37-919.66) | 122.60 | 114.26 | 0.99 | -1.15 | 0 | No |
| Vessel puncture site pain | 1 | 20 | 110.42 (14.81-823.31) | 110.34 | 103.19 | 0.99 | -1.15 | 0 | No |
| Salt craving | 1 | 21 | 105.16 (14.14-782.32) | 105.09 | 98.41 | 0.99 | -1.14 | 0 | No |
| Tri-iodothyronine abnormal | 1 | 21 | 105.16 (14.14-782.32) | 105.09 | 98.41 | 0.99 | -1.14 | 0 | No |
| Splenitis | 1 | 25 | 88.33 (11.96-652.37) | 88.27 | 82.97 | 0.98 | -1.13 | 0 | No |
| Hepatic atrophy | 1 | 25 | 88.33 (11.96-652.37) | 88.27 | 82.97 | 0.98 | -1.13 | 0 | No |
| Nail discomfort | 1 | 25 | 88.33 (11.96-652.37) | 88.27 | 82.97 | 0.98 | -1.13 | 0 | No |
| Testicular torsion | 1 | 26 | 84.94 (11.52-626.35) | 84.88 | 79.82 | 0.98 | -1.13 | 0 | No |
| Biopsy liver | 1 | 26 | 84.94 (11.52-626.35) | 84.88 | 79.82 | 0.98 | -1.13 | 0 | No |
| Haemosiderosis | 1 | 28 | 78.87 (10.72-580.08) | 78.81 | 74.18 | 0.98 | -1.13 | 0 | No |
| Bilirubin conjugated abnormal | 1 | 32 | 69.01 (9.42-505.39) | 68.96 | 64.95 | 0.98 | -1.12 | 0 | No |
| Abscess soft tissue | 1 | 33 | 66.92 (9.15-489.62) | 66.87 | 62.98 | 0.98 | -1.12 | 0 | No |
| Thyroxine abnormal | 1 | 33 | 66.92 (9.15-489.62) | 66.87 | 62.98 | 0.98 | -1.12 | 0 | No |
| Complications of transplanted liver | 1 | 34 | 64.95 (8.88-474.81) | 64.91 | 61.12 | 0.98 | -1.12 | 0 | No |
| Hepatitis C virus test positive | 1 | 38 | 58.11 (7.97-423.56) | 58.07 | 54.65 | 0.97 | -1.12 | 0 | No |
| Tri-iodothyronine free decreased | 1 | 39 | 56.62 (7.77-412.43) | 56.58 | 53.24 | 0.97 | -1.12 | 0 | No |
| Urine albumin/creatinine ratio abnormal | 1 | 40 | 55.21 (7.58-401.87) | 55.17 | 51.89 | 0.97 | -1.12 | 0 | No |
| Blood triglycerides decreased | 1 | 47 | 46.99 (6.48-340.79) | 46.95 | 44.04 | 0.97 | -1.11 | 0 | No |
| Scleral discolouration | 1 | 53 | 41.67 (5.76-301.50) | 41.64 | 38.93 | 0.96 | -1.11 | 0 | No |
| Anticoagulation drug level increased | 1 | 54 | 40.89 (5.65-295.82) | 40.87 | 38.18 | 0.96 | -1.11 | 0 | No |
| Albumin urine present | 1 | 56 | 39.43 (5.45-285.07) | 39.41 | 36.78 | 0.96 | -1.11 | 0 | No |
| Cirrhosis alcoholic | 1 | 57 | 38.74 (5.36-279.98) | 38.72 | 36.11 | 0.96 | -1.11 | 0 | No |
| Fear of eating | 1 | 57 | 38.74 (5.36-279.98) | 38.72 | 36.11 | 0.96 | -1.11 | 0 | No |
| Liver operation | 1 | 59 | 37.43 (5.18-270.34) | 37.40 | 34.84 | 0.96 | -1.11 | 0 | No |
| Ammonia abnormal | 1 | 68 | 32.48 (4.51-234.05) | 32.45 | 30.04 | 0.95 | -1.12 | 0 | No |
| Tinea versicolour | 1 | 69 | 32.00 (4.44-230.61) | 31.98 | 29.59 | 0.95 | -1.12 | 0 | No |
| Acute on chronic liver failure | 1 | 74 | 29.84 (4.15-214.82) | 29.82 | 27.48 | 0.95 | -1.12 | 0 | No |
| Cold flash | 1 | 74 | 29.84 (4.15-214.82) | 29.82 | 27.48 | 0.95 | -1.12 | 0 | No |
| Catheter site induration | 1 | 76 | 29.06 (4.04-209.09) | 29.04 | 26.72 | 0.95 | -1.12 | 0 | No |
| Coating in mouth | 1 | 80 | 27.60 (3.84-198.51) | 27.59 | 25.31 | 0.95 | -1.12 | 0 | No |
| Influenza virus test positive | 1 | 80 | 27.60 (3.84-198.51) | 27.59 | 25.31 | 0.95 | -1.12 | 0 | No |
| Gastric neoplasm | 1 | 84 | 26.29 (3.66-188.95) | 26.27 | 24.02 | 0.94 | -1.12 | 0 | No |
| Urinary tract infection fungal | 1 | 85 | 25.98 (3.62-186.70) | 25.96 | 23.72 | 0.94 | -1.12 | 0 | No |
| Legionella infection | 1 | 86 | 25.68 (3.57-184.50) | 25.66 | 23.43 | 0.94 | -1.12 | 0 | No |
| Gallbladder cancer | 1 | 86 | 25.68 (3.57-184.50) | 25.66 | 23.43 | 0.94 | -1.12 | 0 | No |
| Eyelid infection | 1 | 88 | 25.09 (3.49-180.26) | 25.08 | 22.86 | 0.94 | -1.12 | 0 | No |
| Intraductal papillary mucinous neoplasm | 1 | 88 | 25.09 (3.49-180.26) | 25.08 | 22.86 | 0.94 | -1.12 | 0 | No |
| Bacterial vulvovaginitis | 1 | 89 | 24.81 (3.45-178.22) | 24.80 | 22.58 | 0.94 | -1.12 | 0 | No |
| Antinuclear antibody increased | 1 | 90 | 24.54 (3.42-176.22) | 24.52 | 22.31 | 0.94 | -1.12 | 0 | No |
| Electrolyte depletion | 1 | 91 | 24.27 (3.38-174.26) | 24.25 | 22.05 | 0.94 | -1.12 | 0 | No |
| Hepatic adenoma | 1 | 91 | 24.27 (3.38-174.26) | 24.25 | 22.05 | 0.94 | -1.12 | 0 | No |
| Blood bilirubin unconjugated increased | 1 | 92 | 24.00 (3.34-172.34) | 23.99 | 21.79 | 0.94 | -1.12 | 0 | No |
| Benign neoplasm of thyroid gland | 1 | 93 | 23.75 (3.31-170.47) | 23.73 | 21.54 | 0.94 | -1.12 | 0 | No |
| Genital infection fungal | 1 | 97 | 22.77 (3.17-163.37) | 22.75 | 20.58 | 0.94 | -1.13 | 0 | No |
| Vertebral foraminal stenosis | 1 | 99 | 22.31 (3.11-160.04) | 22.29 | 20.13 | 0.94 | -1.13 | 0 | No |
| Chronic hepatitis | 1 | 100 | 22.08 (3.08-158.42) | 22.07 | 19.91 | 0.93 | -1.13 | 0 | No |
| Vitamin B complex deficiency | 1 | 110 | 20.08 (2.80-143.89) | 20.06 | 17.95 | 0.93 | -1.13 | 0 | No |
| Skin oedema | 1 | 113 | 19.54 (2.73-140.04) | 19.53 | 17.43 | 0.93 | -1.13 | 0 | No |
| Blood urea nitrogen/creatinine ratio increased | 1 | 114 | 19.37 (2.70-138.80) | 19.36 | 17.26 | 0.93 | -1.13 | 0 | No |
| Oesophageal discomfort | 1 | 114 | 19.37 (2.70-138.80) | 19.36 | 17.26 | 0.93 | -1.13 | 0 | No |
| Granulomatous liver disease | 1 | 114 | 19.37 (2.70-138.80) | 19.36 | 17.26 | 0.93 | -1.13 | 0 | No |
| Nutritional condition abnormal | 1 | 130 | 16.99 (2.37-121.59) | 16.98 | 14.92 | 0.92 | -1.14 | 0 | No |
| Creatinine renal clearance increased | 1 | 130 | 16.99 (2.37-121.59) | 16.98 | 14.92 | 0.92 | -1.14 | 0 | No |
| Blood albumin increased | 1 | 135 | 16.36 (2.29-117.05) | 16.35 | 14.30 | 0.91 | -1.14 | 0 | No |
| Enzyme level increased | 1 | 136 | 16.24 (2.27-116.18) | 16.23 | 14.18 | 0.91 | -1.14 | 0 | No |
| Pancreatitis relapsing | 1 | 138 | 16.00 (2.24-114.49) | 15.99 | 13.95 | 0.91 | -1.14 | 0 | No |
| Appendicolith | 1 | 139 | 15.89 (2.22-113.66) | 15.88 | 13.84 | 0.91 | -1.15 | 0 | No |
| Iron overload | 1 | 140 | 15.77 (2.20-112.84) | 15.76 | 13.73 | 0.91 | -1.15 | 0 | No |
| Mean platelet volume increased | 1 | 143 | 15.44 (2.16-110.46) | 15.43 | 13.40 | 0.91 | -1.15 | 0 | No |
| Product appearance confusion | 1 | 144 | 15.34 (2.14-109.69) | 15.33 | 13.30 | 0.91 | -1.15 | 0 | No |
| Traumatic intracranial haemorrhage | 1 | 147 | 15.02 (2.10-107.43) | 15.01 | 12.99 | 0.91 | -1.15 | 0 | No |
| Night blindness | 1 | 149 | 14.82 (2.07-105.98) | 14.81 | 12.79 | 0.90 | -1.15 | 0 | No |
| Pruritus allergic | 1 | 156 | 14.16 (1.98-101.20) | 14.15 | 12.14 | 0.90 | -1.15 | 0 | No |
| Idiopathic interstitial pneumonia | 1 | 157 | 14.07 (1.97-100.55) | 14.06 | 12.05 | 0.90 | -1.15 | 0 | No |
| Aortitis | 1 | 161 | 13.72 (1.92-98.03) | 13.71 | 11.71 | 0.90 | -1.16 | 0 | No |
| Central hypothyroidism | 1 | 162 | 13.63 (1.91-97.42) | 13.62 | 11.62 | 0.90 | -1.16 | 0 | No |
| Cerebrovascular disorder | 1 | 174 | 12.69 (1.78-90.67) | 12.68 | 10.70 | 0.89 | -1.16 | 0 | No |
| Animal scratch | 1 | 181 | 12.20 (1.71-87.14) | 12.19 | 10.22 | 0.89 | -1.17 | 0 | No |
| Syphilis | 1 | 186 | 11.87 (1.66-84.79) | 11.86 | 9.90 | 0.88 | -1.17 | 0 | No |
| Alcohol use | 1 | 188 | 11.75 (1.64-83.88) | 11.74 | 9.77 | 0.88 | -1.17 | 0 | No |
| Appendix disorder | 1 | 188 | 11.75 (1.64-83.88) | 11.74 | 9.77 | 0.88 | -1.17 | 0 | No |
| Solar lentigo | 1 | 197 | 11.21 (1.57-80.03) | 11.20 | 9.25 | 0.88 | -1.18 | 0 | No |
| Colon cancer metastatic | 1 | 199 | 11.10 (1.55-79.22) | 11.09 | 9.13 | 0.87 | -1.18 | 0 | No |
| Infusion site urticaria | 1 | 202 | 10.93 (1.53-78.04) | 10.92 | 8.97 | 0.87 | -1.18 | 0 | No |
| Alcohol abuse | 1 | 203 | 10.88 (1.52-77.65) | 10.87 | 8.92 | 0.87 | -1.18 | 0 | No |
| Non-cardiac chest pain | 1 | 204 | 10.82 (1.52-77.27) | 10.82 | 8.87 | 0.87 | -1.18 | 0 | No |
| Metabolic syndrome | 1 | 220 | 10.04 (1.41-71.62) | 10.03 | 8.09 | 0.86 | -1.19 | 0 | No |
| Muscle discomfort | 1 | 229 | 9.64 (1.35-68.80) | 9.64 | 7.71 | 0.86 | -1.19 | 0 | No |
| Change of bowel habit | 1 | 232 | 9.52 (1.33-67.90) | 9.51 | 7.59 | 0.85 | -1.20 | 0 | No |
| Acrochordon | 1 | 235 | 9.40 (1.32-67.03) | 9.39 | 7.47 | 0.85 | -1.20 | 0 | No |
| Eczema eyelids | 1 | 240 | 9.20 (1.29-65.63) | 9.20 | 7.27 | 0.85 | -1.20 | 0 | No |
| Hyposmia | 1 | 241 | 9.16 (1.28-65.36) | 9.16 | 7.24 | 0.85 | -1.20 | 0 | No |
| Pancreatic enzymes increased | 1 | 247 | 8.94 (1.25-63.76) | 8.93 | 7.02 | 0.85 | -1.20 | 0 | No |
| Intra-abdominal haemorrhage | 1 | 252 | 8.76 (1.23-62.49) | 8.76 | 6.84 | 0.84 | -1.21 | 0 | No |
| Nasal pruritus | 1 | 272 | 8.12 (1.14-57.88) | 8.11 | 6.21 | 0.83 | -1.22 | 0 | No |
| Blood iron increased | 1 | 273 | 8.09 (1.13-57.67) | 8.08 | 6.19 | 0.83 | -1.22 | 0 | No |
| Rotator cuff repair | 1 | 273 | 8.09 (1.13-57.67) | 8.08 | 6.19 | 0.83 | -1.22 | 0 | No |
| Renal mass | 1 | 278 | 7.94 (1.11-56.63) | 7.94 | 6.04 | 0.83 | -1.22 | 0 | No |
| Vertigo positional | 1 | 281 | 7.86 (1.10-56.02) | 7.85 | 5.96 | 0.83 | -1.22 | 0 | No |
| Gingival recession | 1 | 287 | 7.69 (1.08-54.85) | 7.69 | 5.80 | 0.82 | -1.23 | 0 | No |
| Bilirubin conjugated increased | 1 | 290 | 7.61 (1.07-54.28) | 7.61 | 5.72 | 0.82 | -1.23 | 0 | No |
| Metabolic surgery | 1 | 290 | 7.61 (1.07-54.28) | 7.61 | 5.72 | 0.82 | -1.23 | 0 | No |
| Cholangiocarcinoma | 1 | 293 | 7.54 (1.06-53.72) | 7.53 | 5.65 | 0.82 | -1.23 | 0 | No |
| Tongue discolouration | 1 | 294 | 7.51 (1.05-53.54) | 7.51 | 5.62 | 0.82 | -1.23 | 0 | No |
| Glycosylated haemoglobin decreased | 1 | 295 | 7.49 (1.05-53.35) | 7.48 | 5.60 | 0.82 | -1.23 | 0 | No |
| Hypoglycaemic coma | 1 | 314 | 7.03 (0.99-50.11) | 7.03 | 5.15 | 0.81 | -1.24 | 0 | No |
| Creatinine renal clearance decreased | 1 | 332 | 6.65 (0.93-47.39) | 6.65 | 4.78 | 0.80 | -1.25 | 0 | No |
| Miliaria | 1 | 333 | 6.63 (0.93-47.25) | 6.63 | 4.76 | 0.80 | -1.25 | 0 | No |
| Infusion site discomfort | 1 | 348 | 6.35 (0.89-45.20) | 6.34 | 4.49 | 0.79 | -1.26 | 0 | No |
| Leg amputation | 1 | 348 | 6.35 (0.89-45.20) | 6.34 | 4.49 | 0.79 | -1.26 | 0 | No |
| Hepatic mass | 1 | 354 | 6.24 (0.88-44.44) | 6.23 | 4.38 | 0.78 | -1.26 | 0 | No |
| Haemothorax | 1 | 355 | 6.22 (0.87-44.31) | 6.22 | 4.37 | 0.78 | -1.26 | 0 | No |
| Infusion site irritation | 1 | 367 | 6.02 (0.84-42.86) | 6.01 | 4.17 | 0.78 | -1.27 | 0 | No |
| Intra-abdominal fluid collection | 1 | 368 | 6.00 (0.84-42.74) | 6.00 | 4.15 | 0.78 | -1.27 | 0 | No |
| Pancreatic cyst | 1 | 380 | 5.81 (0.82-41.39) | 5.81 | 3.97 | 0.77 | -1.28 | 0 | No |
| Aspartate aminotransferase abnormal | 1 | 382 | 5.78 (0.81-41.17) | 5.78 | 3.94 | 0.77 | -1.28 | 0 | No |
| Lipids increased | 1 | 393 | 5.62 (0.79-40.01) | 5.62 | 3.78 | 0.76 | -1.28 | 0 | No |
| Haemorrhagic diathesis | 1 | 393 | 5.62 (0.79-40.01) | 5.62 | 3.78 | 0.76 | -1.28 | 0 | No |
| Lichen planus | 1 | 402 | 5.49 (0.77-39.12) | 5.49 | 3.66 | 0.76 | -1.29 | 0 | No |
| Infrequent bowel movements | 1 | 403 | 5.48 (0.77-39.02) | 5.48 | 3.65 | 0.76 | -1.29 | 0 | No |
| Conjunctival haemorrhage | 1 | 405 | 5.45 (0.77-38.83) | 5.45 | 3.62 | 0.76 | -1.29 | 0 | No |
| Superficial vein thrombosis | 1 | 408 | 5.41 (0.76-38.54) | 5.41 | 3.59 | 0.75 | -1.29 | 0 | No |
| Hepatic neoplasm | 1 | 411 | 5.37 (0.75-38.26) | 5.37 | 3.55 | 0.75 | -1.29 | 0 | No |
| Duodenitis | 1 | 415 | 5.32 (0.75-37.89) | 5.32 | 3.50 | 0.75 | -1.30 | 0 | No |
| Organ failure | 1 | 415 | 5.32 (0.75-37.89) | 5.32 | 3.50 | 0.75 | -1.30 | 0 | No |
| Neck mass | 1 | 417 | 5.30 (0.74-37.71) | 5.29 | 3.47 | 0.75 | -1.30 | 0 | No |
| Varices oesophageal | 1 | 421 | 5.24 (0.74-37.35) | 5.24 | 3.42 | 0.75 | -1.30 | 0 | No |
| Tumour haemorrhage | 1 | 421 | 5.24 (0.74-37.35) | 5.24 | 3.42 | 0.75 | -1.30 | 0 | No |
| Goitre | 1 | 429 | 5.15 (0.72-36.65) | 5.14 | 3.33 | 0.74 | -1.30 | 0 | No |
| Diabetic neuropathy | 1 | 434 | 5.09 (0.71-36.23) | 5.08 | 3.27 | 0.74 | -1.31 | 0 | No |
| Rectal cancer | 1 | 437 | 5.05 (0.71-35.98) | 5.05 | 3.24 | 0.74 | -1.31 | 0 | No |
| Alanine aminotransferase abnormal | 1 | 444 | 4.97 (0.70-35.41) | 4.97 | 3.16 | 0.73 | -1.31 | 0 | No |
| Oesophageal disorder | 1 | 445 | 4.96 (0.70-35.33) | 4.96 | 3.15 | 0.73 | -1.31 | 0 | No |
| Ear congestion | 1 | 445 | 4.96 (0.70-35.33) | 4.96 | 3.15 | 0.73 | -1.31 | 0 | No |
| Portal hypertension | 1 | 458 | 4.82 (0.68-34.32) | 4.82 | 3.02 | 0.73 | -1.32 | 0 | No |
| Animal bite | 1 | 470 | 4.70 (0.66-33.44) | 4.70 | 2.90 | 0.72 | -1.33 | 0 | No |
| Dermatomyositis | 1 | 478 | 4.62 (0.65-32.88) | 4.62 | 2.83 | 0.72 | -1.33 | 0 | No |
| Allergic reaction to excipient | 1 | 485 | 4.55 (0.64-32.41) | 4.55 | 2.76 | 0.71 | -1.33 | 0 | No |
| Weight abnormal | 1 | 485 | 4.55 (0.64-32.41) | 4.55 | 2.76 | 0.71 | -1.33 | 0 | No |
| Joint noise | 1 | 489 | 4.52 (0.63-32.14) | 4.51 | 2.73 | 0.71 | -1.34 | 0 | No |
| Catheter site pain | 1 | 501 | 4.41 (0.62-31.37) | 4.40 | 2.63 | 0.70 | -1.34 | 0 | No |
| Skin tightness | 1 | 514 | 4.30 (0.60-30.58) | 4.29 | 2.52 | 0.70 | -1.35 | 0 | No |
| Postmenopausal haemorrhage | 1 | 518 | 4.26 (0.60-30.34) | 4.26 | 2.49 | 0.69 | -1.35 | 0 | No |
| Stenosis | 1 | 519 | 4.25 (0.60-30.28) | 4.25 | 2.48 | 0.69 | -1.35 | 0 | No |
| Nail discolouration | 1 | 532 | 4.15 (0.58-29.54) | 4.15 | 2.39 | 0.69 | -1.36 | 0 | No |
| Phlebitis | 1 | 532 | 4.15 (0.58-29.54) | 4.15 | 2.39 | 0.69 | -1.36 | 0 | No |
| Haemorrhoidal haemorrhage | 1 | 533 | 4.14 (0.58-29.48) | 4.14 | 2.38 | 0.69 | -1.36 | 0 | No |
| Blood testosterone decreased | 1 | 558 | 3.96 (0.56-28.16) | 3.95 | 2.20 | 0.67 | -1.37 | 0 | No |
| Dysphemia | 1 | 560 | 3.94 (0.55-28.06) | 3.94 | 2.19 | 0.67 | -1.37 | 0 | No |
| Tic | 1 | 562 | 3.93 (0.55-27.96) | 3.93 | 2.18 | 0.67 | -1.37 | 0 | No |
| Biliary colic | 1 | 565 | 3.91 (0.55-27.81) | 3.91 | 2.16 | 0.67 | -1.38 | 0 | No |
| Trigeminal neuralgia | 1 | 566 | 3.90 (0.55-27.76) | 3.90 | 2.15 | 0.67 | -1.38 | 0 | No |
| Mean cell volume increased | 1 | 574 | 3.85 (0.54-27.37) | 3.84 | 2.10 | 0.67 | -1.38 | 0 | No |
| Lung infiltration | 1 | 593 | 3.72 (0.52-26.50) | 3.72 | 1.99 | 0.66 | -1.39 | 0 | No |
| Vitamin B12 decreased | 1 | 608 | 3.63 (0.51-25.84) | 3.63 | 1.90 | 0.65 | -1.40 | 0 | No |
| Food intolerance | 1 | 624 | 3.54 (0.50-25.18) | 3.54 | 1.82 | 0.64 | -1.41 | 0 | No |
| Hyperuricaemia | 1 | 634 | 3.48 (0.49-24.78) | 3.48 | 1.77 | 0.63 | -1.41 | 0 | No |
| Amylase increased | 1 | 635 | 3.48 (0.49-24.74) | 3.48 | 1.76 | 0.63 | -1.41 | 0 | No |
| Respiratory tract infection viral | 1 | 636 | 3.47 (0.49-24.70) | 3.47 | 1.76 | 0.63 | -1.41 | 0 | No |
| Subcutaneous abscess | 1 | 638 | 3.46 (0.49-24.62) | 3.46 | 1.75 | 0.63 | -1.41 | 0 | No |
| Abdominal tenderness | 1 | 639 | 3.46 (0.49-24.58) | 3.45 | 1.74 | 0.63 | -1.41 | 0 | No |
| Fungal skin infection | 1 | 656 | 3.37 (0.47-23.95) | 3.36 | 1.66 | 0.62 | -1.42 | 0 | No |
| Oxygen saturation abnormal | 1 | 657 | 3.36 (0.47-23.91) | 3.36 | 1.65 | 0.62 | -1.42 | 0 | No |
| Gallbladder operation | 1 | 673 | 3.28 (0.46-23.34) | 3.28 | 1.58 | 0.61 | -1.43 | 0 | No |
| Pulseless electrical activity | 1 | 675 | 3.27 (0.46-23.27) | 3.27 | 1.57 | 0.61 | -1.43 | 0 | No |
| Anti-neutrophil cytoplasmic antibody positive vasculitis | 1 | 685 | 3.22 (0.45-22.93) | 3.22 | 1.53 | 0.61 | -1.44 | 0 | No |
| Autoimmune thyroiditis | 1 | 693 | 3.19 (0.45-22.67) | 3.18 | 1.50 | 0.60 | -1.44 | 0 | No |
| Blood cholesterol abnormal | 1 | 720 | 3.07 (0.43-21.81) | 3.07 | 1.39 | 0.59 | -1.45 | 0 | No |
| Urinary tract disorder | 1 | 726 | 3.04 (0.43-21.63) | 3.04 | 1.37 | 0.59 | -1.46 | 0 | No |
| Trigger finger | 1 | 735 | 3.00 (0.42-21.37) | 3.00 | 1.33 | 0.58 | -1.46 | 0 | No |
| Sarcoidosis | 1 | 752 | 2.94 (0.41-20.88) | 2.93 | 1.27 | 0.58 | -1.47 | 0 | No |
| Muscle fatigue | 1 | 754 | 2.93 (0.41-20.83) | 2.93 | 1.27 | 0.57 | -1.47 | 0 | No |
| Bell's palsy | 1 | 777 | 2.84 (0.40-20.21) | 2.84 | 1.19 | 0.56 | -1.48 | 0 | No |
| Feeling of despair | 1 | 782 | 2.82 (0.40-20.08) | 2.82 | 1.18 | 0.56 | -1.48 | 0 | No |
| Photopsia | 1 | 793 | 2.78 (0.39-19.80) | 2.78 | 1.14 | 0.56 | -1.49 | 0 | No |
| Tongue discomfort | 1 | 797 | 2.77 (0.39-19.70) | 2.77 | 1.13 | 0.55 | -1.49 | 0 | No |
| Diverticulum intestinal | 1 | 798 | 2.77 (0.39-19.68) | 2.77 | 1.13 | 0.55 | -1.49 | 0 | No |
| Immune-mediated hepatitis | 1 | 802 | 2.75 (0.39-19.58) | 2.75 | 1.11 | 0.55 | -1.49 | 0 | No |
| Macular oedema | 1 | 805 | 2.74 (0.39-19.51) | 2.74 | 1.11 | 0.55 | -1.49 | 0 | No |
| Inguinal hernia | 1 | 830 | 2.66 (0.37-18.92) | 2.66 | 1.03 | 0.54 | -1.51 | 0 | No |
| Dyslipidaemia | 1 | 837 | 2.64 (0.37-18.76) | 2.64 | 1.01 | 0.54 | -1.51 | 0 | No |
| Oral mucosal blistering | 1 | 841 | 2.63 (0.37-18.67) | 2.62 | 1.00 | 0.53 | -1.51 | 0 | No |
| Gastric cancer | 1 | 858 | 2.57 (0.36-18.30) | 2.57 | 0.96 | 0.53 | -1.52 | 0 | No |
| Bone erosion | 1 | 862 | 2.56 (0.36-18.22) | 2.56 | 0.95 | 0.52 | -1.52 | 0 | No |
| Rales | 1 | 864 | 2.56 (0.36-18.17) | 2.55 | 0.94 | 0.52 | -1.52 | 0 | No |
| Renal cancer | 1 | 895 | 2.47 (0.35-17.54) | 2.47 | 0.87 | 0.51 | -1.54 | 0 | No |
| Norovirus infection | 1 | 896 | 2.46 (0.35-17.52) | 2.46 | 0.87 | 0.51 | -1.54 | 0 | No |
| Hepatitis acute | 1 | 898 | 2.46 (0.35-17.48) | 2.46 | 0.86 | 0.51 | -1.54 | 0 | No |
| Sjogren's syndrome | 1 | 913 | 2.42 (0.34-17.20) | 2.42 | 0.83 | 0.50 | -1.54 | 0 | No |
| Eosinophil count decreased | 1 | 913 | 2.42 (0.34-17.20) | 2.42 | 0.83 | 0.50 | -1.54 | 0 | No |
| Ulcer haemorrhage | 1 | 935 | 2.36 (0.33-16.79) | 2.36 | 0.78 | 0.49 | -1.55 | 0 | No |
| Gastric haemorrhage | 1 | 940 | 2.35 (0.33-16.70) | 2.35 | 0.77 | 0.49 | -1.56 | 0 | No |
| Renal cyst | 1 | 947 | 2.33 (0.33-16.58) | 2.33 | 0.76 | 0.48 | -1.56 | 0 | No |
| Ear pruritus | 1 | 959 | 2.30 (0.32-16.37) | 2.30 | 0.74 | 0.48 | -1.57 | 0 | No |
| Nasal discomfort | 1 | 965 | 2.29 (0.32-16.27) | 2.29 | 0.72 | 0.48 | -1.57 | 0 | No |
| Psychiatric symptom | 1 | 971 | 2.27 (0.32-16.17) | 2.27 | 0.71 | 0.47 | -1.57 | 0 | No |
| Faecaloma | 1 | 971 | 2.27 (0.32-16.17) | 2.27 | 0.71 | 0.47 | -1.57 | 0 | No |
| Uterine leiomyoma | 1 | 977 | 2.26 (0.32-16.07) | 2.26 | 0.70 | 0.47 | -1.57 | 0 | No |
| Proctalgia | 1 | 988 | 2.23 (0.31-15.89) | 2.23 | 0.68 | 0.47 | -1.58 | 0 | No |
| Near death experience | 1 | 995 | 2.22 (0.31-15.78) | 2.22 | 0.67 | 0.46 | -1.58 | 0 | No |
| Odynophagia | 1 | 1015 | 2.18 (0.31-15.47) | 2.17 | 0.63 | 0.45 | -1.59 | 0 | No |
| Infusion site haemorrhage | 1 | 1054 | 2.09 (0.29-14.89) | 2.09 | 0.57 | 0.44 | -1.61 | 0 | No |
| Hepatocellular carcinoma | 1 | 1057 | 2.09 (0.29-14.85) | 2.09 | 0.57 | 0.43 | -1.61 | 0 | No |
| Purpura | 1 | 1059 | 2.08 (0.29-14.82) | 2.08 | 0.56 | 0.43 | -1.61 | 0 | No |
| Parosmia | 1 | 1088 | 2.03 (0.29-14.43) | 2.03 | 0.52 | 0.42 | -1.62 | 0 | No |
| Haemolytic anaemia | 1 | 1117 | 1.98 (0.28-14.05) | 1.98 | 0.48 | 0.41 | -1.64 | 0 | No |
| Atelectasis | 1 | 1121 | 1.97 (0.28-14.00) | 1.97 | 0.48 | 0.41 | -1.64 | 0 | No |
| Gingival pain | 1 | 1123 | 1.97 (0.28-13.98) | 1.97 | 0.47 | 0.41 | -1.64 | 0 | No |
| Epstein-Barr virus infection | 1 | 1158 | 1.91 (0.27-13.55) | 1.91 | 0.43 | 0.39 | -1.65 | 0 | No |
| Hordeolum | 1 | 1162 | 1.90 (0.27-13.51) | 1.90 | 0.43 | 0.39 | -1.65 | 0 | No |
| Vitamin D decreased | 1 | 1164 | 1.90 (0.27-13.48) | 1.90 | 0.42 | 0.39 | -1.66 | 0 | No |
| Postoperative wound infection | 1 | 1179 | 1.87 (0.26-13.31) | 1.87 | 0.41 | 0.38 | -1.66 | 0 | No |
| Food poisoning | 1 | 1186 | 1.86 (0.26-13.23) | 1.86 | 0.40 | 0.38 | -1.66 | 0 | No |
| Appetite disorder | 1 | 1198 | 1.84 (0.26-13.10) | 1.84 | 0.38 | 0.37 | -1.67 | 0 | No |
| Spinal stenosis | 1 | 1213 | 1.82 (0.26-12.94) | 1.82 | 0.37 | 0.37 | -1.68 | 0 | No |
| Muscle strain | 1 | 1218 | 1.81 (0.25-12.89) | 1.81 | 0.36 | 0.36 | -1.68 | 0 | No |
| Electric shock sensation | 1 | 1227 | 1.80 (0.25-12.79) | 1.80 | 0.35 | 0.36 | -1.68 | 0 | No |
| Tooth abscess | 1 | 1235 | 1.79 (0.25-12.71) | 1.79 | 0.35 | 0.36 | -1.69 | 0 | No |
| Blood thyroid stimulating hormone increased | 1 | 1242 | 1.78 (0.25-12.64) | 1.78 | 0.34 | 0.35 | -1.69 | 0 | No |
| Bladder disorder | 1 | 1247 | 1.77 (0.25-12.59) | 1.77 | 0.33 | 0.35 | -1.69 | 0 | No |
| Hiccups | 1 | 1254 | 1.76 (0.25-12.52) | 1.76 | 0.33 | 0.35 | -1.69 | 0 | No |
| Tendon rupture | 1 | 1378 | 1.60 (0.23-11.39) | 1.60 | 0.23 | 0.30 | -1.74 | 0 | No |
| Iron deficiency anaemia | 1 | 1387 | 1.59 (0.22-11.31) | 1.59 | 0.22 | 0.30 | -1.75 | 0 | No |
| Impaired driving ability | 1 | 1390 | 1.59 (0.22-11.29) | 1.59 | 0.22 | 0.29 | -1.75 | 0 | No |
| Facial pain | 1 | 1401 | 1.58 (0.22-11.20) | 1.58 | 0.21 | 0.29 | -1.75 | 0 | No |
| Dry throat | 1 | 1487 | 1.48 (0.21-10.55) | 1.48 | 0.16 | 0.26 | -1.79 | 0 | No |
| Motor dysfunction | 1 | 1509 | 1.46 (0.21-10.40) | 1.46 | 0.15 | 0.25 | -1.80 | 0 | No |
| Bowel movement irregularity | 1 | 1522 | 1.45 (0.20-10.31) | 1.45 | 0.14 | 0.24 | -1.80 | 0 | No |
| Hiatus hernia | 1 | 1527 | 1.45 (0.20-10.28) | 1.45 | 0.14 | 0.24 | -1.80 | 0 | No |
| Salivary hypersecretion | 1 | 1542 | 1.43 (0.20-10.18) | 1.43 | 0.13 | 0.23 | -1.81 | 0 | No |
| Rotator cuff syndrome | 1 | 1636 | 1.35 (0.19-9.59) | 1.35 | 0.09 | 0.20 | -1.84 | 0 | No |
| Eyelid ptosis | 1 | 1685 | 1.31 (0.18-9.31) | 1.31 | 0.07 | 0.18 | -1.86 | 0 | No |
| Blepharospasm | 1 | 1696 | 1.30 (0.18-9.25) | 1.30 | 0.07 | 0.18 | -1.87 | 0 | No |
| Gastrointestinal infection | 1 | 1742 | 1.27 (0.18-9.01) | 1.27 | 0.06 | 0.16 | -1.88 | 0 | No |
| Thinking abnormal | 1 | 1767 | 1.25 (0.18-8.88) | 1.25 | 0.05 | 0.15 | -1.89 | 0 | No |
| Red blood cell sedimentation rate increased | 1 | 1768 | 1.25 (0.18-8.87) | 1.25 | 0.05 | 0.15 | -1.89 | 0 | No |
| Myositis | 1 | 1805 | 1.22 (0.17-8.69) | 1.22 | 0.04 | 0.14 | -1.91 | 0 | No |
| Temperature intolerance | 1 | 1808 | 1.22 (0.17-8.68) | 1.22 | 0.04 | 0.14 | -1.91 | 0 | No |
| Osteopenia | 1 | 1813 | 1.22 (0.17-8.65) | 1.22 | 0.04 | 0.13 | -1.91 | 0 | No |
| Dysmenorrhoea | 1 | 1826 | 1.21 (0.17-8.59) | 1.21 | 0.04 | 0.13 | -1.91 | 0 | No |
| Nail disorder | 1 | 1832 | 1.20 (0.17-8.56) | 1.20 | 0.03 | 0.13 | -1.92 | 0 | No |
| Gallbladder disorder | 1 | 1844 | 1.20 (0.17-8.51) | 1.20 | 0.03 | 0.12 | -1.92 | 0 | No |
| Ligament sprain | 1 | 1850 | 1.19 (0.17-8.48) | 1.19 | 0.03 | 0.12 | -1.92 | 0 | No |
| Pancreatic carcinoma | 1 | 1900 | 1.16 (0.16-8.26) | 1.16 | 0.02 | 0.10 | -1.94 | 0 | No |
| Peripheral coldness | 1 | 1900 | 1.16 (0.16-8.26) | 1.16 | 0.02 | 0.10 | -1.94 | 0 | No |
| Infusion site swelling | 1 | 1932 | 1.14 (0.16-8.12) | 1.14 | 0.02 | 0.09 | -1.95 | 0 | No |
| Feeling jittery | 1 | 1985 | 1.11 (0.16-7.90) | 1.11 | 0.01 | 0.07 | -1.97 | 0 | No |
| Muscle tightness | 1 | 2003 | 1.10 (0.16-7.83) | 1.10 | 0.01 | 0.07 | -1.98 | 0 | No |
| Prostatic specific antigen increased | 1 | 2005 | 1.10 (0.15-7.82) | 1.10 | 0.01 | 0.07 | -1.98 | 0 | No |
| Hypervolaemia | 1 | 2010 | 1.10 (0.15-7.80) | 1.10 | 0.01 | 0.06 | -1.98 | 0 | No |
| Blindness unilateral | 1 | 2017 | 1.09 (0.15-7.78) | 1.09 | 0.01 | 0.06 | -1.98 | 0 | No |
| Face oedema | 1 | 2018 | 1.09 (0.15-7.77) | 1.09 | 0.01 | 0.06 | -1.98 | 0 | No |
| Dermatitis contact | 1 | 2023 | 1.09 (0.15-7.75) | 1.09 | 0.01 | 0.06 | -1.98 | 0 | No |
| Initial insomnia | 1 | 2027 | 1.09 (0.15-7.74) | 1.09 | 0.01 | 0.06 | -1.98 | 0 | No |
| Defaecation urgency | 1 | 2042 | 1.08 (0.15-7.68) | 1.08 | 0.01 | 0.05 | -1.99 | 0 | No |
| Sensitive skin | 1 | 2047 | 1.08 (0.15-7.66) | 1.08 | 0.01 | 0.05 | -1.99 | 0 | No |
| Skin infection | 1 | 2072 | 1.07 (0.15-7.57) | 1.07 | 0.00 | 0.04 | -2.00 | 0 | No |
| Periorbital swelling | 1 | 2087 | 1.06 (0.15-7.52) | 1.06 | 0.00 | 0.04 | -2.00 | 0 | No |
| Ventricular tachycardia | 1 | 2098 | 1.05 (0.15-7.48) | 1.05 | 0.00 | 0.04 | -2.01 | 0 | No |
| Impaired work ability | 1 | 2143 | 1.03 (0.14-7.32) | 1.03 | 0.00 | 0.02 | -2.02 | 0 | No |
| Eye discharge | 1 | 2145 | 1.03 (0.14-7.31) | 1.03 | 0.00 | 0.02 | -2.02 | 0 | No |
| Decreased activity | 1 | 2170 | 1.02 (0.14-7.23) | 1.02 | 0.00 | 0.01 | -2.03 | 0 | No |
| Gastroenteritis | 1 | 2223 | 0.99 (0.14-7.06) | 0.99 | 0.00 | -0.01 | -2.05 | 0 | No |
| Knee operation | 1 | 2264 | 0.97 (0.14-6.93) | 0.97 | 0.00 | -0.02 | -2.06 | 0 | No |
| Back disorder | 1 | 2281 | 0.97 (0.14-6.88) | 0.97 | 0.00 | -0.02 | -2.07 | 0 | No |
| Heart rate irregular | 1 | 2282 | 0.97 (0.14-6.87) | 0.97 | 0.00 | -0.03 | -2.07 | 0 | No |
| Paraesthesia oral | 1 | 2303 | 0.96 (0.13-6.81) | 0.96 | 0.00 | -0.03 | -2.07 | 0 | No |
| Blood urine present | 1 | 2318 | 0.95 (0.13-6.77) | 0.95 | 0.00 | -0.04 | -2.08 | 0 | No |
| Restless legs syndrome | 1 | 2320 | 0.95 (0.13-6.76) | 0.95 | 0.00 | -0.04 | -2.08 | 0 | No |
| Acute myeloid leukaemia | 1 | 2367 | 0.93 (0.13-6.63) | 0.93 | 0.00 | -0.05 | -2.09 | 0 | No |
| Nocturia | 1 | 2386 | 0.92 (0.13-6.57) | 0.92 | 0.01 | -0.06 | -2.10 | 0 | No |
| Abnormal dreams | 1 | 2394 | 0.92 (0.13-6.55) | 0.92 | 0.01 | -0.06 | -2.10 | 0 | No |
| Dermatitis allergic | 1 | 2404 | 0.92 (0.13-6.52) | 0.92 | 0.01 | -0.06 | -2.11 | 0 | No |
| Pulmonary hypertension | 1 | 2418 | 0.91 (0.13-6.49) | 0.91 | 0.01 | -0.07 | -2.11 | 0 | No |
| Infusion site erythema | 1 | 2448 | 0.90 (0.13-6.41) | 0.90 | 0.01 | -0.08 | -2.12 | 0 | No |
| Thyroid disorder | 1 | 2453 | 0.90 (0.13-6.39) | 0.90 | 0.01 | -0.08 | -2.12 | 0 | No |
| Acute hepatic failure | 1 | 2460 | 0.90 (0.13-6.38) | 0.90 | 0.01 | -0.08 | -2.12 | 0 | No |
| Blood glucose abnormal | 1 | 2479 | 0.89 (0.13-6.33) | 0.89 | 0.01 | -0.09 | -2.13 | 0 | No |
| Abscess | 1 | 2541 | 0.87 (0.12-6.17) | 0.87 | 0.02 | -0.11 | -2.15 | 0 | No |
| Therapy change | 1 | 2597 | 0.85 (0.12-6.04) | 0.85 | 0.03 | -0.12 | -2.17 | 0 | No |
| Blood sodium decreased | 1 | 2618 | 0.84 (0.12-5.99) | 0.84 | 0.03 | -0.13 | -2.17 | 0 | No |
| Scratch | 1 | 2640 | 0.84 (0.12-5.94) | 0.84 | 0.03 | -0.14 | -2.18 | 0 | No |
| Intervertebral disc protrusion | 1 | 2644 | 0.83 (0.12-5.93) | 0.83 | 0.03 | -0.14 | -2.18 | 0 | No |
| Blood iron decreased | 1 | 2649 | 0.83 (0.12-5.92) | 0.83 | 0.03 | -0.14 | -2.18 | 0 | No |
| Toothache | 1 | 2677 | 0.82 (0.12-5.86) | 0.82 | 0.04 | -0.15 | -2.19 | 0 | No |
| Pulmonary mass | 1 | 2691 | 0.82 (0.12-5.83) | 0.82 | 0.04 | -0.15 | -2.19 | 0 | No |
| Gingival bleeding | 1 | 2693 | 0.82 (0.12-5.82) | 0.82 | 0.04 | -0.15 | -2.19 | 0 | No |
| Blood test abnormal | 1 | 2708 | 0.81 (0.11-5.79) | 0.81 | 0.04 | -0.16 | -2.20 | 0 | No |
| Musculoskeletal discomfort | 1 | 2709 | 0.81 (0.11-5.79) | 0.81 | 0.04 | -0.16 | -2.20 | 0 | No |
| Muscle twitching | 1 | 2742 | 0.80 (0.11-5.72) | 0.80 | 0.05 | -0.17 | -2.21 | 0 | No |
| Glomerular filtration rate decreased | 1 | 2794 | 0.79 (0.11-5.61) | 0.79 | 0.06 | -0.18 | -2.22 | 0 | No |
| Eating disorder | 1 | 2879 | 0.77 (0.11-5.45) | 0.77 | 0.07 | -0.21 | -2.25 | 0 | No |
| Ocular discomfort | 1 | 2891 | 0.76 (0.11-5.42) | 0.76 | 0.07 | -0.21 | -2.25 | 0 | No |
| Hypocalcaemia | 1 | 3029 | 0.73 (0.10-5.18) | 0.73 | 0.10 | -0.25 | -2.29 | 0 | No |
| Pain of skin | 1 | 3099 | 0.71 (0.10-5.06) | 0.71 | 0.12 | -0.27 | -2.31 | 0 | No |
| Uveitis | 1 | 3194 | 0.69 (0.10-4.91) | 0.69 | 0.14 | -0.29 | -2.33 | 0 | No |
| Mood swings | 1 | 3231 | 0.68 (0.10-4.85) | 0.68 | 0.15 | -0.30 | -2.34 | 0 | No |
| Spinal operation | 1 | 3297 | 0.67 (0.09-4.75) | 0.67 | 0.16 | -0.32 | -2.36 | 0 | No |
| Candida infection | 1 | 3307 | 0.67 (0.09-4.74) | 0.67 | 0.17 | -0.32 | -2.36 | 0 | No |
| Acute respiratory failure | 1 | 3368 | 0.65 (0.09-4.65) | 0.66 | 0.18 | -0.34 | -2.38 | 0 | No |
| Helicobacter infection | 1 | 3382 | 0.65 (0.09-4.64) | 0.65 | 0.19 | -0.34 | -2.38 | 0 | No |
| Infusion site pain | 1 | 3384 | 0.65 (0.09-4.63) | 0.65 | 0.19 | -0.34 | -2.38 | 0 | No |
| Joint injury | 1 | 3408 | 0.65 (0.09-4.60) | 0.65 | 0.19 | -0.35 | -2.39 | 0 | No |
| Skin cancer | 1 | 3420 | 0.65 (0.09-4.58) | 0.65 | 0.20 | -0.35 | -2.39 | 0 | No |
| Ankylosing spondylitis | 1 | 3421 | 0.64 (0.09-4.58) | 0.65 | 0.20 | -0.35 | -2.39 | 0 | No |
| Sciatica | 1 | 3454 | 0.64 (0.09-4.54) | 0.64 | 0.20 | -0.36 | -2.40 | 0 | No |
| Skin ulcer | 1 | 3466 | 0.64 (0.09-4.52) | 0.64 | 0.21 | -0.36 | -2.41 | 0 | No |
| Crying | 1 | 3496 | 0.63 (0.09-4.48) | 0.63 | 0.22 | -0.37 | -2.41 | 0 | No |
| Shock | 1 | 3526 | 0.63 (0.09-4.45) | 0.63 | 0.22 | -0.38 | -2.42 | 0 | No |
| Throat tightness | 1 | 3551 | 0.62 (0.09-4.41) | 0.62 | 0.23 | -0.38 | -2.43 | 0 | No |
| Seasonal allergy | 1 | 3574 | 0.62 (0.09-4.39) | 0.62 | 0.24 | -0.39 | -2.43 | 0 | No |
| Mouth ulceration | 1 | 3613 | 0.61 (0.09-4.34) | 0.61 | 0.25 | -0.40 | -2.44 | 0 | No |
| Gastroenteritis viral | 1 | 3631 | 0.61 (0.09-4.32) | 0.61 | 0.25 | -0.40 | -2.45 | 0 | No |
| Aphasia | 1 | 3700 | 0.60 (0.08-4.24) | 0.60 | 0.27 | -0.42 | -2.46 | 0 | No |
| Pulmonary fibrosis | 1 | 3724 | 0.59 (0.08-4.21) | 0.59 | 0.28 | -0.43 | -2.47 | 0 | No |
| Haematuria | 1 | 3747 | 0.59 (0.08-4.18) | 0.59 | 0.29 | -0.43 | -2.47 | 0 | No |
| Joint stiffness | 1 | 3756 | 0.59 (0.08-4.17) | 0.59 | 0.29 | -0.43 | -2.48 | 0 | No |
| Increased appetite | 1 | 3917 | 0.56 (0.08-4.00) | 0.56 | 0.34 | -0.47 | -2.52 | 0 | No |
| Diplopia | 1 | 3933 | 0.56 (0.08-3.98) | 0.56 | 0.34 | -0.48 | -2.52 | 0 | No |
| Erectile dysfunction | 1 | 3988 | 0.55 (0.08-3.93) | 0.55 | 0.36 | -0.49 | -2.53 | 0 | No |
| Conjunctivitis | 1 | 4042 | 0.55 (0.08-3.88) | 0.55 | 0.38 | -0.50 | -2.54 | 0 | No |
| Clostridium difficile infection | 1 | 4103 | 0.54 (0.08-3.82) | 0.54 | 0.40 | -0.52 | -2.56 | 0 | No |
| Skin lesion | 1 | 4353 | 0.51 (0.07-3.60) | 0.51 | 0.48 | -0.57 | -2.61 | 0 | No |
| Anaphylactic shock | 1 | 4405 | 0.50 (0.07-3.56) | 0.50 | 0.50 | -0.58 | -2.63 | 0 | No |
| Respiratory disorder | 1 | 4504 | 0.49 (0.07-3.48) | 0.49 | 0.53 | -0.61 | -2.65 | 0 | No |
| Emotional disorder | 1 | 4587 | 0.48 (0.07-3.42) | 0.48 | 0.56 | -0.62 | -2.67 | 0 | No |
| Limb discomfort | 1 | 4662 | 0.47 (0.07-3.36) | 0.47 | 0.59 | -0.64 | -2.68 | 0 | No |
| Dysuria | 1 | 4665 | 0.47 (0.07-3.36) | 0.47 | 0.59 | -0.64 | -2.68 | 0 | No |
| Feeding disorder | 1 | 4923 | 0.45 (0.06-3.18) | 0.45 | 0.68 | -0.69 | -2.73 | 0 | No |
| Urinary retention | 1 | 4925 | 0.45 (0.06-3.18) | 0.45 | 0.68 | -0.69 | -2.74 | 0 | No |
| Type 2 diabetes mellitus | 1 | 5028 | 0.44 (0.06-3.12) | 0.44 | 0.72 | -0.71 | -2.76 | 0 | No |
| Heart rate decreased | 1 | 5055 | 0.44 (0.06-3.10) | 0.44 | 0.73 | -0.72 | -2.76 | 0 | No |
| Fungal infection | 1 | 5120 | 0.43 (0.06-3.06) | 0.43 | 0.75 | -0.73 | -2.77 | 0 | No |
| Product use complaint | 1 | 5121 | 0.43 (0.06-3.06) | 0.43 | 0.75 | -0.73 | -2.77 | 0 | No |
| Eye disorder | 1 | 5161 | 0.43 (0.06-3.04) | 0.43 | 0.77 | -0.74 | -2.78 | 0 | No |
| Epilepsy | 1 | 5169 | 0.43 (0.06-3.03) | 0.43 | 0.77 | -0.74 | -2.78 | 0 | No |
| Respiratory tract infection | 1 | 5531 | 0.40 (0.06-2.83) | 0.40 | 0.91 | -0.81 | -2.85 | 0 | No |
| Rectal haemorrhage | 1 | 5581 | 0.39 (0.06-2.81) | 0.40 | 0.93 | -0.82 | -2.86 | 0 | No |
| Hypoxia | 1 | 5591 | 0.39 (0.06-2.80) | 0.39 | 0.93 | -0.82 | -2.86 | 0 | No |
| Impaired healing | 1 | 5633 | 0.39 (0.06-2.78) | 0.39 | 0.95 | -0.83 | -2.87 | 0 | No |
| Hypothyroidism | 1 | 5787 | 0.38 (0.05-2.71) | 0.38 | 1.01 | -0.86 | -2.90 | 0 | No |
| Pulmonary oedema | 1 | 5804 | 0.38 (0.05-2.70) | 0.38 | 1.01 | -0.86 | -2.90 | 0 | No |
| Skin discolouration | 1 | 6205 | 0.36 (0.05-2.52) | 0.36 | 1.17 | -0.93 | -2.97 | 0 | No |
| Injury | 1 | 6235 | 0.35 (0.05-2.51) | 0.35 | 1.18 | -0.94 | -2.98 | 0 | No |
| Irritability | 1 | 6406 | 0.34 (0.05-2.44) | 0.34 | 1.25 | -0.97 | -3.01 | 0 | No |
| Cellulitis | 1 | 6418 | 0.34 (0.05-2.44) | 0.34 | 1.25 | -0.97 | -3.01 | 0 | No |
| Tinnitus | 1 | 6472 | 0.34 (0.05-2.42) | 0.34 | 1.28 | -0.98 | -3.02 | 0 | No |
| Speech disorder | 1 | 6572 | 0.34 (0.05-2.38) | 0.34 | 1.32 | -0.99 | -3.03 | 0 | No |
| Cognitive disorder | 1 | 6909 | 0.32 (0.04-2.27) | 0.32 | 1.45 | -1.05 | -3.09 | 0 | No |
| Chronic obstructive pulmonary disease | 1 | 7046 | 0.31 (0.04-2.22) | 0.31 | 1.51 | -1.07 | -3.11 | 0 | No |
| Emotional distress | 1 | 7092 | 0.31 (0.04-2.21) | 0.31 | 1.53 | -1.08 | -3.12 | 0 | No |
| Intestinal obstruction | 1 | 7150 | 0.31 (0.04-2.19) | 0.31 | 1.55 | -1.08 | -3.13 | 0 | No |
| Hypokalaemia | 1 | 7217 | 0.31 (0.04-2.17) | 0.31 | 1.58 | -1.09 | -3.14 | 0 | No |
| Throat irritation | 1 | 7447 | 0.30 (0.04-2.10) | 0.30 | 1.67 | -1.13 | -3.17 | 0 | No |
| Acne | 1 | 7584 | 0.29 (0.04-2.06) | 0.29 | 1.73 | -1.15 | -3.19 | 0 | No |
| Hypoacusis | 1 | 7637 | 0.29 (0.04-2.05) | 0.29 | 1.75 | -1.16 | -3.20 | 0 | No |
| Agitation | 1 | 7743 | 0.28 (0.04-2.02) | 0.29 | 1.80 | -1.17 | -3.22 | 0 | No |
| Pleural effusion | 1 | 7856 | 0.28 (0.04-1.99) | 0.28 | 1.85 | -1.19 | -3.23 | 0 | No |
| Upper respiratory tract infection | 1 | 7941 | 0.28 (0.04-1.97) | 0.28 | 1.88 | -1.20 | -3.24 | 0 | No |
| Eye pruritus | 1 | 7964 | 0.28 (0.04-1.97) | 0.28 | 1.89 | -1.20 | -3.25 | 0 | No |
| Oedema | 1 | 8590 | 0.26 (0.04-1.82) | 0.26 | 2.16 | -1.29 | -3.33 | 0 | No |
| Depressed mood | 1 | 8609 | 0.26 (0.04-1.82) | 0.26 | 2.16 | -1.29 | -3.34 | 0 | No |
| Skin burning sensation | 1 | 8640 | 0.25 (0.04-1.81) | 0.26 | 2.18 | -1.30 | -3.34 | 0 | No |
| Respiratory failure | 1 | 9539 | 0.23 (0.03-1.64) | 0.23 | 2.56 | -1.41 | -3.45 | 0 | No |
| Psoriatic arthropathy | 1 | 9901 | 0.22 (0.03-1.58) | 0.22 | 2.72 | -1.46 | -3.50 | 0 | No |
| Productive cough | 1 | 10042 | 0.22 (0.03-1.56) | 0.22 | 2.78 | -1.47 | -3.52 | 0 | No |
| Neoplasm malignant | 1 | 10253 | 0.21 (0.03-1.53) | 0.22 | 2.87 | -1.50 | -3.54 | 0 | No |
| Lower respiratory tract infection | 1 | 10386 | 0.21 (0.03-1.51) | 0.21 | 2.93 | -1.51 | -3.55 | 0 | No |
| Cardiac disorder | 1 | 10667 | 0.21 (0.03-1.47) | 0.21 | 3.05 | -1.54 | -3.59 | 0 | No |
| Cardiac failure | 1 | 10879 | 0.20 (0.03-1.44) | 0.20 | 3.14 | -1.57 | -3.61 | 0 | No |
| Oxygen saturation decreased | 1 | 10933 | 0.20 (0.03-1.43) | 0.20 | 3.17 | -1.57 | -3.62 | 0 | No |
| Underdose | 1 | 10971 | 0.20 (0.03-1.43) | 0.20 | 3.18 | -1.58 | -3.62 | 0 | No |
| Treatment noncompliance | 1 | 11166 | 0.20 (0.03-1.40) | 0.20 | 3.27 | -1.60 | -3.64 | 0 | No |
| Suicidal ideation | 1 | 11814 | 0.19 (0.03-1.32) | 0.19 | 3.55 | -1.67 | -3.71 | 0 | No |
| Eye irritation | 1 | 12010 | 0.18 (0.03-1.30) | 0.18 | 3.64 | -1.69 | -3.73 | 0 | No |
| Dry eye | 1 | 12625 | 0.17 (0.02-1.24) | 0.17 | 3.91 | -1.75 | -3.79 | 0 | No |
| Drug ineffective for unapproved indication | 1 | 13346 | 0.16 (0.02-1.17) | 0.17 | 4.23 | -1.82 | -3.86 | 0 | No |
| Renal impairment | 1 | 13507 | 0.16 (0.02-1.16) | 0.16 | 4.30 | -1.83 | -3.87 | 0 | No |
| Extra dose administered | 1 | 13526 | 0.16 (0.02-1.15) | 0.16 | 4.31 | -1.83 | -3.88 | 0 | No |
| Exposure via skin contact | 1 | 14870 | 0.15 (0.02-1.05) | 0.15 | 4.91 | -1.95 | -3.99 | 0 | No |
| Sepsis | 1 | 15681 | 0.14 (0.02-1.00) | 0.14 | 5.27 | -2.02 | -4.06 | 0 | No |
| Cerebrovascular accident | 1 | 17198 | 0.13 (0.02-0.91) | 0.13 | 5.95 | -2.14 | -4.18 | 0 | No |
| Thrombocytopenia | 1 | 17501 | 0.13 (0.02-0.89) | 0.13 | 6.09 | -2.16 | -4.20 | 0 | No |
| Overdose | 1 | 19210 | 0.11 (0.02-0.81) | 0.11 | 6.86 | -2.28 | -4.32 | 0 | No |
| Joint swelling | 1 | 20408 | 0.11 (0.02-0.76) | 0.11 | 7.40 | -2.36 | -4.40 | 0 | No |
| Infection | 1 | 26457 | 0.08 (0.01-0.59) | 0.08 | 10.16 | -2.70 | -4.74 | 0 | No |
| Asthma | 1 | 26570 | 0.08 (0.01-0.59) | 0.08 | 10.21 | -2.71 | -4.75 | 0 | No |
| Accidental exposure to product | 1 | 29762 | 0.07 (0.01-0.52) | 0.07 | 11.67 | -2.86 | -4.90 | 0 | No |
| Incorrect dose administered | 1 | 59036 | 0.04 (0.01-0.26) | 0.04 | 25.26 | -3.79 | -5.84 | 0 | No |

Note: PTs with fewer than three target reports were retained in the raw computational CSV but were not evaluated in the prespecified hypothesis-generating disproportionality assessment.

**Supplementary Table S10. Complete clinical-cluster disproportionality results.**

| **Event** | **a** | **c** | **ROR (95% CI)** | **PRR** | **Chi-square** | **IC** | **IC025** | **Criteria met** | **All 3 criteria** |
| --- | --- | --- | --- | --- | --- | --- | --- | --- | --- |
| hepatocellular_injury | 261 | 57031 | 12.13 (10.61-13.88) | 10.10 | 2169.43 | 3.28 | 3.09 | 3 | Yes |
| cholestasis_biliary | 89 | 18176 | 11.46 (9.24-14.21) | 10.81 | 792.68 | 3.28 | 2.96 | 3 | Yes |
| gastrointestinal_intolerance | 486 | 321348 | 4.54 (4.07-5.07) | 3.34 | 884.71 | 1.73 | 1.58 | 3 | Yes |
| thyroid_metabolic | 103 | 54034 | 4.46 (3.65-5.45) | 4.21 | 255.68 | 2.03 | 1.73 | 3 | Yes |
| muscle_toxicity | 34 | 72419 | 1.04 (0.74-1.46) | 1.04 | 0.04 | 0.05 | -0.45 | 0 | No |
| neuropsychiatric | 171 | 226740 | 1.75 (1.50-2.06) | 1.66 | 48.82 | 0.73 | 0.49 | 2 | No |
| cardiovascular | 19 | 112730 | 0.36 (0.23-0.57) | 0.37 | 20.89 | -1.38 | -2.03 | 0 | No |
| renal | 10 | 46540 | 0.47 (0.25-0.88) | 0.47 | 5.92 | -1.01 | -1.88 | 0 | No |
| rash_hypersensitivity | 272 | 187340 | 3.72 (3.26-4.25) | 3.20 | 437.84 | 1.67 | 1.48 | 3 | Yes |

Note: Prespecified clusters group clinically related MedDRA PTs and are intended for interpretation alongside PT-level outputs. Each PT appears in one dictionary cluster only, while a report may contribute to multiple clusters through different PTs. The full attribution, rationale, development timing, and overlap rule are reported in Supplementary Table S1.

**Supplementary Table S11. Sensitivity analyses for predefined clinical clusters, including the exploratory active-comparator analysis.**

| **Scenario** | **Target n** | **Reference n** | **Event** | **a** | **c** | **ROR (95% CI)** | **PRR** | **Chi-square** | **IC** | **IC025** | **Criteria met** | **All 3 criteria** |
| --- | --- | --- | --- | --- | --- | --- | --- | --- | --- | --- | --- | --- |
| main_ps_ss_vs_faers_non_resmetirom | 1429 | 3153534 | hepatocellular_injury | 261 | 57031 | 12.13 (10.61-13.88) | 10.10 | 2169.43 | 3.28 | 3.09 | 3 | Yes |
| main_ps_ss_vs_faers_non_resmetirom | 1429 | 3153534 | cholestasis_biliary | 89 | 18176 | 11.46 (9.24-14.21) | 10.81 | 792.68 | 3.28 | 2.96 | 3 | Yes |
| main_ps_ss_vs_faers_non_resmetirom | 1429 | 3153534 | gastrointestinal_intolerance | 486 | 321348 | 4.54 (4.07-5.07) | 3.34 | 884.71 | 1.73 | 1.58 | 3 | Yes |
| main_ps_ss_vs_faers_non_resmetirom | 1429 | 3153534 | thyroid_metabolic | 103 | 54034 | 4.46 (3.65-5.45) | 4.21 | 255.68 | 2.03 | 1.73 | 3 | Yes |
| main_ps_ss_vs_faers_non_resmetirom | 1429 | 3153534 | muscle_toxicity | 34 | 72419 | 1.04 (0.74-1.46) | 1.04 | 0.04 | 0.05 | -0.45 | 0 | No |
| main_ps_ss_vs_faers_non_resmetirom | 1429 | 3153534 | neuropsychiatric | 171 | 226740 | 1.75 (1.50-2.06) | 1.66 | 48.82 | 0.73 | 0.49 | 2 | No |
| main_ps_ss_vs_faers_non_resmetirom | 1429 | 3153534 | cardiovascular | 19 | 112730 | 0.36 (0.23-0.57) | 0.37 | 20.89 | -1.38 | -2.03 | 0 | No |
| main_ps_ss_vs_faers_non_resmetirom | 1429 | 3153534 | renal | 10 | 46540 | 0.47 (0.25-0.88) | 0.47 | 5.92 | -1.01 | -1.88 | 0 | No |
| main_ps_ss_vs_faers_non_resmetirom | 1429 | 3153534 | rash_hypersensitivity | 272 | 187340 | 3.72 (3.26-4.25) | 3.20 | 437.84 | 1.67 | 1.48 | 3 | Yes |
| primary_suspect_only | 1407 | 3153534 | hepatocellular_injury | 257 | 57031 | 12.13 (10.60-13.89) | 10.10 | 2136.51 | 3.28 | 3.08 | 3 | Yes |
| primary_suspect_only | 1407 | 3153534 | cholestasis_biliary | 89 | 18176 | 11.65 (9.39-14.44) | 10.97 | 807.61 | 3.30 | 2.98 | 3 | Yes |
| primary_suspect_only | 1407 | 3153534 | gastrointestinal_intolerance | 470 | 321348 | 4.42 (3.96-4.94) | 3.28 | 827.41 | 1.70 | 1.55 | 3 | Yes |
| primary_suspect_only | 1407 | 3153534 | thyroid_metabolic | 99 | 54034 | 4.34 (3.54-5.33) | 4.11 | 236.28 | 1.99 | 1.69 | 3 | Yes |
| primary_suspect_only | 1407 | 3153534 | muscle_toxicity | 32 | 72419 | 0.99 (0.70-1.41) | 0.99 | 0.00 | -0.01 | -0.52 | 0 | No |
| primary_suspect_only | 1407 | 3153534 | neuropsychiatric | 164 | 226740 | 1.70 (1.45-2.00) | 1.62 | 42.02 | 0.69 | 0.45 | 2 | No |
| primary_suspect_only | 1407 | 3153534 | cardiovascular | 18 | 112730 | 0.35 (0.22-0.56) | 0.36 | 21.50 | -1.43 | -2.10 | 0 | No |
| primary_suspect_only | 1407 | 3153534 | renal | 9 | 46540 | 0.43 (0.22-0.83) | 0.43 | 6.76 | -1.12 | -2.04 | 0 | No |
| primary_suspect_only | 1407 | 3153534 | rash_hypersensitivity | 266 | 187340 | 3.69 (3.23-4.22) | 3.18 | 422.67 | 1.66 | 1.46 | 3 | Yes |
| ps_ss_interacting | 1429 | 3153534 | hepatocellular_injury | 261 | 57031 | 12.13 (10.61-13.88) | 10.10 | 2169.43 | 3.28 | 3.09 | 3 | Yes |
| ps_ss_interacting | 1429 | 3153534 | cholestasis_biliary | 89 | 18176 | 11.46 (9.24-14.21) | 10.81 | 792.68 | 3.28 | 2.96 | 3 | Yes |
| ps_ss_interacting | 1429 | 3153534 | gastrointestinal_intolerance | 486 | 321348 | 4.54 (4.07-5.07) | 3.34 | 884.71 | 1.73 | 1.58 | 3 | Yes |
| ps_ss_interacting | 1429 | 3153534 | thyroid_metabolic | 103 | 54034 | 4.46 (3.65-5.45) | 4.21 | 255.68 | 2.03 | 1.73 | 3 | Yes |
| ps_ss_interacting | 1429 | 3153534 | muscle_toxicity | 34 | 72419 | 1.04 (0.74-1.46) | 1.04 | 0.04 | 0.05 | -0.45 | 0 | No |
| ps_ss_interacting | 1429 | 3153534 | neuropsychiatric | 171 | 226740 | 1.75 (1.50-2.06) | 1.66 | 48.82 | 0.73 | 0.49 | 2 | No |
| ps_ss_interacting | 1429 | 3153534 | cardiovascular | 19 | 112730 | 0.36 (0.23-0.57) | 0.37 | 20.89 | -1.38 | -2.03 | 0 | No |
| ps_ss_interacting | 1429 | 3153534 | renal | 10 | 46540 | 0.47 (0.25-0.88) | 0.47 | 5.92 | -1.01 | -1.88 | 0 | No |
| ps_ss_interacting | 1429 | 3153534 | rash_hypersensitivity | 272 | 187340 | 3.72 (3.26-4.25) | 3.20 | 437.84 | 1.67 | 1.48 | 3 | Yes |
| all_roles_exploratory | 1519 | 3153534 | hepatocellular_injury | 261 | 57031 | 11.26 (9.86-12.87) | 9.50 | 2012.74 | 3.20 | 3.00 | 3 | Yes |
| all_roles_exploratory | 1519 | 3153534 | cholestasis_biliary | 89 | 18176 | 10.74 (8.66-13.31) | 10.17 | 736.17 | 3.20 | 2.89 | 3 | Yes |
| all_roles_exploratory | 1519 | 3153534 | gastrointestinal_intolerance | 508 | 321348 | 4.43 (3.98-4.93) | 3.28 | 896.15 | 1.71 | 1.56 | 3 | Yes |
| all_roles_exploratory | 1519 | 3153534 | thyroid_metabolic | 110 | 54034 | 4.48 (3.69-5.44) | 4.23 | 275.10 | 2.03 | 1.75 | 3 | Yes |
| all_roles_exploratory | 1519 | 3153534 | muscle_toxicity | 35 | 72419 | 1.00 (0.72-1.40) | 1.00 | 0.00 | 0.00 | -0.48 | 0 | No |
| all_roles_exploratory | 1519 | 3153534 | neuropsychiatric | 179 | 226740 | 1.72 (1.48-2.02) | 1.64 | 48.01 | 0.71 | 0.48 | 2 | No |
| all_roles_exploratory | 1519 | 3153534 | cardiovascular | 22 | 112730 | 0.40 (0.26-0.60) | 0.41 | 19.92 | -1.27 | -1.87 | 0 | No |
| all_roles_exploratory | 1519 | 3153534 | renal | 10 | 46540 | 0.44 (0.24-0.82) | 0.45 | 6.98 | -1.09 | -1.96 | 0 | No |
| all_roles_exploratory | 1519 | 3153534 | rash_hypersensitivity | 286 | 187340 | 3.67 (3.23-4.18) | 3.17 | 450.85 | 1.65 | 1.47 | 3 | Yes |
| exclude_glp1_gip | 1266 | 2961107 | hepatocellular_injury | 216 | 55658 | 10.74 (9.27-12.44) | 9.08 | 1576.17 | 3.12 | 2.91 | 3 | Yes |
| exclude_glp1_gip | 1266 | 2961107 | cholestasis_biliary | 76 | 15849 | 11.87 (9.41-14.97) | 11.22 | 707.61 | 3.30 | 2.96 | 3 | Yes |
| exclude_glp1_gip | 1266 | 2961107 | gastrointestinal_intolerance | 417 | 278117 | 4.74 (4.21-5.33) | 3.51 | 823.62 | 1.80 | 1.64 | 3 | Yes |
| exclude_glp1_gip | 1266 | 2961107 | thyroid_metabolic | 84 | 48248 | 4.29 (3.44-5.35) | 4.07 | 197.57 | 1.97 | 1.65 | 3 | Yes |
| exclude_glp1_gip | 1266 | 2961107 | muscle_toxicity | 26 | 69400 | 0.87 (0.59-1.29) | 0.88 | 0.47 | -0.18 | -0.75 | 0 | No |
| exclude_glp1_gip | 1266 | 2961107 | neuropsychiatric | 145 | 212946 | 1.67 (1.40-1.98) | 1.59 | 34.43 | 0.66 | 0.41 | 2 | No |
| exclude_glp1_gip | 1266 | 2961107 | cardiovascular | 18 | 108238 | 0.38 (0.24-0.61) | 0.39 | 17.93 | -1.32 | -1.98 | 0 | No |
| exclude_glp1_gip | 1266 | 2961107 | renal | 9 | 44666 | 0.47 (0.24-0.90) | 0.47 | 5.42 | -1.01 | -1.92 | 0 | No |
| exclude_glp1_gip | 1266 | 2961107 | rash_hypersensitivity | 249 | 181796 | 3.74 (3.26-4.30) | 3.20 | 401.58 | 1.66 | 1.46 | 3 | Yes |
| exclude_statin | 1162 | 2993835 | hepatocellular_injury | 181 | 52065 | 10.42 (8.89-12.22) | 8.96 | 1297.60 | 3.10 | 2.87 | 3 | Yes |
| exclude_statin | 1162 | 2993835 | cholestasis_biliary | 64 | 16253 | 10.68 (8.30-13.75) | 10.15 | 528.42 | 3.15 | 2.78 | 3 | Yes |
| exclude_statin | 1162 | 2993835 | gastrointestinal_intolerance | 389 | 293657 | 4.63 (4.10-5.23) | 3.41 | 734.89 | 1.76 | 1.59 | 3 | Yes |
| exclude_statin | 1162 | 2993835 | thyroid_metabolic | 78 | 48271 | 4.39 (3.49-5.53) | 4.16 | 190.24 | 2.00 | 1.66 | 3 | Yes |
| exclude_statin | 1162 | 2993835 | muscle_toxicity | 14 | 60723 | 0.59 (0.35-1.00) | 0.59 | 3.96 | -0.71 | -1.46 | 0 | No |
| exclude_statin | 1162 | 2993835 | neuropsychiatric | 125 | 207025 | 1.62 (1.35-1.95) | 1.56 | 26.63 | 0.63 | 0.36 | 2 | No |
| exclude_statin | 1162 | 2993835 | cardiovascular | 14 | 100726 | 0.35 (0.21-0.59) | 0.36 | 16.67 | -1.42 | -2.17 | 0 | No |
| exclude_statin | 1162 | 2993835 | renal | 7 | 39806 | 0.45 (0.21-0.95) | 0.45 | 4.68 | -1.04 | -2.06 | 0 | No |
| exclude_statin | 1162 | 2993835 | rash_hypersensitivity | 224 | 176643 | 3.81 (3.29-4.41) | 3.27 | 374.05 | 1.69 | 1.48 | 3 | Yes |
| exclude_hepatotoxic_drugs | 1350 | 2824038 | hepatocellular_injury | 241 | 39747 | 15.22 (13.24-17.50) | 12.68 | 2615.18 | 3.59 | 3.39 | 3 | Yes |
| exclude_hepatotoxic_drugs | 1350 | 2824038 | cholestasis_biliary | 83 | 14466 | 12.72 (10.18-15.90) | 12.00 | 836.64 | 3.40 | 3.07 | 3 | Yes |
| exclude_hepatotoxic_drugs | 1350 | 2824038 | gastrointestinal_intolerance | 453 | 276813 | 4.65 (4.15-5.20) | 3.42 | 860.24 | 1.76 | 1.61 | 3 | Yes |
| exclude_hepatotoxic_drugs | 1350 | 2824038 | thyroid_metabolic | 95 | 44407 | 4.74 (3.85-5.84) | 4.48 | 259.92 | 2.11 | 1.80 | 3 | Yes |
| exclude_hepatotoxic_drugs | 1350 | 2824038 | muscle_toxicity | 32 | 61028 | 1.10 (0.77-1.56) | 1.10 | 0.28 | 0.13 | -0.38 | 0 | No |
| exclude_hepatotoxic_drugs | 1350 | 2824038 | neuropsychiatric | 156 | 200419 | 1.71 (1.45-2.02) | 1.63 | 40.67 | 0.70 | 0.45 | 2 | No |
| exclude_hepatotoxic_drugs | 1350 | 2824038 | cardiovascular | 17 | 92305 | 0.38 (0.23-0.61) | 0.39 | 17.23 | -1.33 | -2.01 | 0 | No |
| exclude_hepatotoxic_drugs | 1350 | 2824038 | renal | 9 | 36111 | 0.52 (0.27-1.00) | 0.52 | 4.00 | -0.87 | -1.79 | 0 | No |
| exclude_hepatotoxic_drugs | 1350 | 2824038 | rash_hypersensitivity | 258 | 164633 | 3.82 (3.33-4.37) | 3.28 | 433.12 | 1.70 | 1.50 | 3 | Yes |
| metabolic_background_reference | 1429 | 340363 | hepatocellular_injury | 261 | 4015 | 18.72 (16.31-21.48) | 15.48 | 3362.24 | 3.79 | 3.59 | 3 | Yes |
| metabolic_background_reference | 1429 | 340363 | cholestasis_biliary | 89 | 3294 | 6.80 (5.47-8.45) | 6.44 | 401.81 | 2.57 | 2.25 | 3 | Yes |
| metabolic_background_reference | 1429 | 340363 | gastrointestinal_intolerance | 486 | 59808 | 2.42 (2.17-2.70) | 1.94 | 264.66 | 0.94 | 0.79 | 2 | No |
| metabolic_background_reference | 1429 | 340363 | thyroid_metabolic | 103 | 12443 | 2.05 (1.67-2.50) | 1.97 | 50.78 | 0.96 | 0.67 | 2 | No |
| metabolic_background_reference | 1429 | 340363 | muscle_toxicity | 34 | 7355 | 1.10 (0.78-1.55) | 1.10 | 0.32 | 0.13 | -0.36 | 0 | No |
| metabolic_background_reference | 1429 | 340363 | neuropsychiatric | 171 | 25186 | 1.70 (1.45-2.00) | 1.62 | 43.21 | 0.68 | 0.45 | 2 | No |
| metabolic_background_reference | 1429 | 340363 | cardiovascular | 19 | 14118 | 0.31 (0.20-0.49) | 0.32 | 28.51 | -1.59 | -2.24 | 0 | No |
| metabolic_background_reference | 1429 | 340363 | renal | 10 | 8931 | 0.26 (0.14-0.49) | 0.27 | 20.68 | -1.80 | -2.68 | 0 | No |
| metabolic_background_reference | 1429 | 340363 | rash_hypersensitivity | 272 | 11993 | 6.44 (5.63-7.35) | 5.40 | 989.56 | 2.38 | 2.19 | 3 | Yes |
| exclude_target_cirrhosis_indication | 1394 | 3153534 | hepatocellular_injury | 254 | 57031 | 12.10 (10.56-13.86) | 10.08 | 2105.35 | 3.28 | 3.08 | 3 | Yes |
| exclude_target_cirrhosis_indication | 1394 | 3153534 | cholestasis_biliary | 86 | 18176 | 11.34 (9.11-14.11) | 10.70 | 757.38 | 3.26 | 2.94 | 3 | Yes |
| exclude_target_cirrhosis_indication | 1394 | 3153534 | gastrointestinal_intolerance | 477 | 321348 | 4.58 (4.10-5.12) | 3.36 | 878.22 | 1.74 | 1.58 | 3 | Yes |
| exclude_target_cirrhosis_indication | 1394 | 3153534 | thyroid_metabolic | 101 | 54034 | 4.48 (3.66-5.49) | 4.23 | 252.84 | 2.03 | 1.74 | 3 | Yes |
| exclude_target_cirrhosis_indication | 1394 | 3153534 | muscle_toxicity | 34 | 72419 | 1.06 (0.76-1.49) | 1.06 | 0.13 | 0.08 | -0.41 | 0 | No |
| exclude_target_cirrhosis_indication | 1394 | 3153534 | neuropsychiatric | 165 | 226740 | 1.73 (1.47-2.04) | 1.65 | 45.07 | 0.71 | 0.48 | 2 | No |
| exclude_target_cirrhosis_indication | 1394 | 3153534 | cardiovascular | 19 | 112730 | 0.37 (0.24-0.59) | 0.38 | 19.78 | -1.35 | -2.00 | 0 | No |
| exclude_target_cirrhosis_indication | 1394 | 3153534 | renal | 10 | 46540 | 0.48 (0.26-0.90) | 0.49 | 5.51 | -0.97 | -1.85 | 0 | No |
| exclude_target_cirrhosis_indication | 1394 | 3153534 | rash_hypersensitivity | 270 | 187340 | 3.80 (3.33-4.34) | 3.26 | 449.22 | 1.69 | 1.50 | 3 | Yes |
| us_mash_indication_restricted | 1017 | 218 | hepatocellular_injury | 217 | 6 | 9.58 (4.20-21.87) | 7.75 | 41.91 | 0.24 | -0.02 | 2 | No |
| us_mash_indication_restricted | 1017 | 218 | cholestasis_biliary | 80 | 2 | 9.22 (2.25-37.80) | 8.57 | 13.98 | 0.23 | -0.21 | 2 | No |
| us_mash_indication_restricted | 1017 | 218 | gastrointestinal_intolerance | 365 | 49 | 1.93 (1.37-2.72) | 1.60 | 14.49 | 0.10 | -0.08 | 1 | No |
| us_mash_indication_restricted | 1017 | 218 | thyroid_metabolic | 81 | 7 | 2.61 (1.19-5.73) | 2.48 | 6.13 | 0.15 | -0.28 | 2 | No |
| us_mash_indication_restricted | 1017 | 218 | muscle_toxicity | 26 | 9 | 0.61 (0.28-1.32) | 0.62 | 1.61 | -0.18 | -0.91 | 0 | No |
| us_mash_indication_restricted | 1017 | 218 | neuropsychiatric | 139 | 19 | 1.66 (1.00-2.74) | 1.57 | 3.95 | 0.09 | -0.23 | 1 | No |
| us_mash_indication_restricted | 1017 | 218 | cardiovascular | 14 | 1 | 3.03 (0.40-23.16) | 3.00 | 1.26 | 0.08 | -0.95 | 0 | No |
| us_mash_indication_restricted | 1017 | 218 | renal | 8 | 8 | 0.21 (0.08-0.56) | 0.21 | 11.67 | -0.73 | -1.92 | 0 | No |
| us_mash_indication_restricted | 1017 | 218 | rash_hypersensitivity | 192 | 7 | 7.02 (3.25-15.14) | 5.88 | 32.60 | 0.22 | -0.05 | 2 | No |
| us_active_comparator_mash | 1017 | 144 | hepatocellular_injury | 217 | 1 | 38.79 (5.40-278.85) | 30.73 | 35.24 | 0.18 | -0.07 | 2 | No |
| us_active_comparator_mash | 1017 | 144 | cholestasis_biliary | 80 | 1 | 12.21 (1.69-88.43) | 11.33 | 10.00 | 0.16 | -0.28 | 2 | No |
| us_active_comparator_mash | 1017 | 144 | gastrointestinal_intolerance | 365 | 42 | 1.36 (0.93-1.99) | 1.23 | 2.50 | 0.03 | -0.14 | 0 | No |
| us_active_comparator_mash | 1017 | 144 | thyroid_metabolic | 81 | 4 | 3.03 (1.09-8.40) | 2.87 | 5.00 | 0.11 | -0.32 | 2 | No |
| us_active_comparator_mash | 1017 | 144 | muscle_toxicity | 26 | 1 | 3.75 (0.51-27.86) | 3.68 | 1.93 | 0.08 | -0.69 | 0 | No |
| us_active_comparator_mash | 1017 | 144 | neuropsychiatric | 139 | 6 | 3.64 (1.58-8.41) | 3.28 | 10.42 | 0.12 | -0.20 | 2 | No |
| us_active_comparator_mash | 1017 | 144 | cardiovascular | 14 | 1 | 2.00 (0.26-15.29) | 1.98 | 0.46 | 0.00 | -1.03 | 0 | No |
| us_active_comparator_mash | 1017 | 144 | renal | 8 | 1 | 1.13 (0.14-9.13) | 1.13 | 0.01 | -0.11 | -1.43 | 0 | No |
| us_active_comparator_mash | 1017 | 144 | rash_hypersensitivity | 192 | 2 | 16.52 (4.06-67.31) | 13.59 | 27.73 | 0.17 | -0.10 | 2 | No |
| complete_age_sex_only | 63 | 1819373 | hepatocellular_injury | 10 | 38989 | 8.62 (4.38-16.94) | 7.41 | 56.62 | 2.21 | 1.27 | 3 | Yes |
| complete_age_sex_only | 63 | 1819373 | cholestasis_biliary | 5 | 13144 | 11.85 (4.75-29.54) | 10.99 | 45.70 | 2.04 | 0.80 | 3 | Yes |
| complete_age_sex_only | 63 | 1819373 | gastrointestinal_intolerance | 29 | 205987 | 6.68 (4.07-10.96) | 4.07 | 75.59 | 1.86 | 1.22 | 3 | Yes |
| complete_age_sex_only | 63 | 1819373 | thyroid_metabolic | 3 | 34840 | 2.56 (0.80-8.17) | 2.49 | 2.72 | 0.85 | -0.64 | 0 | No |
| complete_age_sex_only | 63 | 1819373 | muscle_toxicity | 2 | 47902 | 1.21 (0.30-4.96) | 1.21 | 0.07 | 0.16 | -1.54 | 0 | No |
| complete_age_sex_only | 63 | 1819373 | neuropsychiatric | 19 | 139685 | 5.19 (3.03-8.89) | 3.93 | 44.91 | 1.76 | 1.02 | 3 | Yes |
| complete_age_sex_only | 63 | 1819373 | cardiovascular | 1 | 74797 | 0.38 (0.05-2.71) | 0.39 | 1.02 | -0.86 | -2.93 | 0 | No |
| complete_age_sex_only | 63 | 1819373 | renal | 1 | 34161 | 0.84 (0.12-6.08) | 0.85 | 0.03 | -0.14 | -2.21 | 0 | No |
| complete_age_sex_only | 63 | 1819373 | rash_hypersensitivity | 18 | 125272 | 5.41 (3.13-9.34) | 4.15 | 46.21 | 1.81 | 1.06 | 3 | Yes |

Note: Sensitivity analyses included exclusion of cirrhosis-indication target reports and US MASH-indication/active-comparator references. The active comparator comprised 144 reports, including 142 GLP-1/GIP and 2 pioglitazone reports; vitamin E contributed no eligible report. It was selected before execution of this comparator analysis as an indication-adjacent treatment context, but was not specified in the original protocol or preregistered; it is exploratory, underpowered, and not a definitive safety benchmark. Gastrointestinal attenuation may reflect a reporting background shared with GLP-1/GIP therapies and does not show that resmetirom lacks gastrointestinal risk. Because all target reports were from the United States, geographic restriction did not create a distinct target population; a separate US-only scenario was therefore not reported.

**Supplementary Table S12. Cirrhosis-indication subgroup and cirrhosis-PT context.**

Note: Among 35 reports with a cirrhosis-related indication, 15 (42.9%) contained any serious-outcome code, 1 (2.9%) contained a death code, and 8 (22.9%) also contained a cirrhosis adverse-event PT. Clinical-cluster counts were gastrointestinal intolerance 9/35, hepatocellular injury 7/35, neuropsychiatric events 6/35, cholestasis/biliary events 3/35, thyroid/metabolic events 2/35, and rash/hypersensitivity 2/35; no muscle, cardiovascular, or renal cluster event was recorded. Cirrhosis-indication coding may reflect off-label use, pre-existing disease, legacy terminology, delayed staging, or miscoding and should not automatically be interpreted as an incident adverse event.

| **Measure** | **n** | **Denominator** | **%** |
| --- | --- | --- | --- |
| Target reports with a cirrhosis-related indication | 35 | 1429 | 2.4 |
| Target reports with a cirrhosis-related adverse-event PT | 71 | 1429 | 5.0 |
| Reports with both a cirrhosis-related indication and adverse-event PT | 8 | 35 | 22.9 |
| Cirrhosis-indication reports with any serious outcome code | 15 | 35 | 42.9 |
| Cirrhosis-indication reports with death outcome code | 1 | 35 | 2.9 |
| Cirrhosis-indication reports in cluster: hepatocellular_injury | 7 | 35 | 20.0 |
| Cirrhosis-indication reports in cluster: cholestasis_biliary | 3 | 35 | 8.6 |
| Cirrhosis-indication reports in cluster: gastrointestinal_intolerance | 9 | 35 | 25.7 |
| Cirrhosis-indication reports in cluster: thyroid_metabolic | 2 | 35 | 5.7 |
| Cirrhosis-indication reports in cluster: muscle_toxicity | 0 | 35 | 0.0 |
| Cirrhosis-indication reports in cluster: neuropsychiatric | 6 | 35 | 17.1 |
| Cirrhosis-indication reports in cluster: cardiovascular | 0 | 35 | 0.0 |
| Cirrhosis-indication reports in cluster: renal | 0 | 35 | 0.0 |
| Cirrhosis-indication reports in cluster: rash_hypersensitivity | 2 | 35 | 5.7 |

**Supplementary Table S13. Sensitivity analyses for SOC-level reporting disproportionality.**

| **Scenario** | **Target n** | **Reference n** | **Event** | **a** | **c** | **ROR (95% CI)** | **PRR** | **Chi-square** | **IC** | **IC025** | **Criteria met** | **All 3 criteria** |
| --- | --- | --- | --- | --- | --- | --- | --- | --- | --- | --- | --- | --- |
| main_ps_ss_vs_faers_non_resmetirom | 1429 | 3153534 | Investigations | 438 | 351080 | 3.53 (3.15-3.95) | 2.75 | 549.60 | 1.45 | 1.30 | 3 | Yes |
| main_ps_ss_vs_faers_non_resmetirom | 1429 | 3153534 | Nervous system disorders | 217 | 479908 | 1.00 (0.86-1.15) | 1.00 | 0.00 | -0.00 | -0.21 | 0 | No |
| main_ps_ss_vs_faers_non_resmetirom | 1429 | 3153534 | Injury, poisoning and procedural complications | 134 | 997506 | 0.22 (0.19-0.27) | 0.30 | 327.16 | -1.75 | -2.01 | 0 | No |
| main_ps_ss_vs_faers_non_resmetirom | 1429 | 3153534 | Gastrointestinal disorders | 548 | 479527 | 3.47 (3.12-3.86) | 2.52 | 592.96 | 1.33 | 1.18 | 3 | Yes |
| main_ps_ss_vs_faers_non_resmetirom | 1429 | 3153534 | Skin and subcutaneous tissue disorders | 342 | 370802 | 2.36 (2.09-2.67) | 2.04 | 203.96 | 1.02 | 0.85 | 3 | Yes |
| main_ps_ss_vs_faers_non_resmetirom | 1429 | 3153534 | Hepatobiliary disorders | 258 | 77914 | 8.70 (7.60-9.95) | 7.31 | 1435.58 | 2.83 | 2.63 | 3 | Yes |
| main_ps_ss_vs_faers_non_resmetirom | 1429 | 3153534 | General disorders and administration site conditions | 457 | 1161805 | 0.81 (0.72-0.90) | 0.87 | 14.51 | -0.20 | -0.36 | 0 | No |
| main_ps_ss_vs_faers_non_resmetirom | 1429 | 3153534 | Respiratory, thoracic and mediastinal disorders | 43 | 299269 | 0.30 (0.22-0.40) | 0.32 | 69.87 | -1.64 | -2.08 | 0 | No |
| main_ps_ss_vs_faers_non_resmetirom | 1429 | 3153534 | Musculoskeletal and connective tissue disorders | 86 | 289676 | 0.63 (0.51-0.79) | 0.66 | 17.18 | -0.61 | -0.92 | 0 | No |
| main_ps_ss_vs_faers_non_resmetirom | 1429 | 3153534 | Infections and infestations | 68 | 390576 | 0.35 (0.28-0.45) | 0.38 | 76.58 | -1.37 | -1.72 | 0 | No |
| main_ps_ss_vs_faers_non_resmetirom | 1429 | 3153534 | Vascular disorders | 18 | 159911 | 0.24 (0.15-0.38) | 0.25 | 43.11 | -1.95 | -2.62 | 0 | No |
| main_ps_ss_vs_faers_non_resmetirom | 1429 | 3153534 | Metabolism and nutrition disorders | 64 | 160622 | 0.87 (0.68-1.12) | 0.88 | 1.12 | -0.18 | -0.55 | 0 | No |
| main_ps_ss_vs_faers_non_resmetirom | 1429 | 3153534 | Social circumstances | 11 | 46547 | 0.52 (0.29-0.94) | 0.52 | 4.90 | -0.88 | -1.72 | 0 | No |
| main_ps_ss_vs_faers_non_resmetirom | 1429 | 3153534 | Renal and urinary disorders | 52 | 121194 | 0.94 (0.72-1.25) | 0.95 | 0.16 | -0.08 | -0.48 | 0 | No |
| main_ps_ss_vs_faers_non_resmetirom | 1429 | 3153534 | Psychiatric disorders | 77 | 270660 | 0.61 (0.48-0.76) | 0.63 | 18.58 | -0.67 | -1.00 | 0 | No |
| main_ps_ss_vs_faers_non_resmetirom | 1429 | 3153534 | Blood and lymphatic system disorders | 16 | 138429 | 0.25 (0.15-0.40) | 0.26 | 36.40 | -1.91 | -2.61 | 0 | No |
| main_ps_ss_vs_faers_non_resmetirom | 1429 | 3153534 | Immune system disorders | 33 | 107490 | 0.67 (0.47-0.95) | 0.68 | 5.24 | -0.55 | -1.05 | 0 | No |
| main_ps_ss_vs_faers_non_resmetirom | 1429 | 3153534 | Cardiac disorders | 19 | 144400 | 0.28 (0.18-0.44) | 0.29 | 34.53 | -1.73 | -2.38 | 0 | No |
| main_ps_ss_vs_faers_non_resmetirom | 1429 | 3153534 | Neoplasms benign, malignant and unspecified (incl cysts and polyps) | 20 | 144181 | 0.30 (0.19-0.46) | 0.31 | 32.96 | -1.66 | -2.29 | 0 | No |
| main_ps_ss_vs_faers_non_resmetirom | 1429 | 3153534 | Reproductive system and breast disorders | 8 | 47421 | 0.37 (0.18-0.74) | 0.37 | 8.59 | -1.32 | -2.29 | 0 | No |
| main_ps_ss_vs_faers_non_resmetirom | 1429 | 3153534 | Ear and labyrinth disorders | 8 | 34761 | 0.51 (0.25-1.01) | 0.51 | 3.86 | -0.90 | -1.86 | 0 | No |
| main_ps_ss_vs_faers_non_resmetirom | 1429 | 3153534 | Surgical and medical procedures | 77 | 155123 | 1.10 (0.87-1.39) | 1.10 | 0.67 | 0.13 | -0.21 | 0 | No |
| main_ps_ss_vs_faers_non_resmetirom | 1429 | 3153534 | Product issues | 4 | 184346 | 0.05 (0.02-0.12) | 0.05 | 80.42 | -4.08 | -5.37 | 0 | No |
| main_ps_ss_vs_faers_non_resmetirom | 1429 | 3153534 | Eye disorders | 32 | 147964 | 0.47 (0.33-0.66) | 0.48 | 19.22 | -1.04 | -1.55 | 0 | No |
| main_ps_ss_vs_faers_non_resmetirom | 1429 | 3153534 | Endocrine disorders | 5 | 26342 | 0.42 (0.17-1.00) | 0.42 | 4.06 | -1.11 | -2.29 | 0 | No |
| main_ps_ss_vs_faers_non_resmetirom | 1429 | 3153534 | Congenital, familial and genetic disorders | 2 | 18671 | 0.24 (0.06-0.94) | 0.24 | 4.96 | -1.66 | -3.33 | 0 | No |
| primary_suspect_only | 1407 | 3153534 | Investigations | 428 | 351080 | 3.49 (3.11-3.91) | 2.73 | 528.40 | 1.44 | 1.28 | 3 | Yes |
| primary_suspect_only | 1407 | 3153534 | Nervous system disorders | 209 | 479908 | 0.97 (0.84-1.13) | 0.98 | 0.14 | -0.04 | -0.25 | 0 | No |
| primary_suspect_only | 1407 | 3153534 | Injury, poisoning and procedural complications | 128 | 997506 | 0.22 (0.18-0.26) | 0.29 | 330.28 | -1.79 | -2.06 | 0 | No |
| primary_suspect_only | 1407 | 3153534 | Gastrointestinal disorders | 530 | 479527 | 3.37 (3.03-3.75) | 2.48 | 550.06 | 1.30 | 1.16 | 3 | Yes |
| primary_suspect_only | 1407 | 3153534 | Skin and subcutaneous tissue disorders | 333 | 370802 | 2.33 (2.06-2.63) | 2.01 | 192.16 | 1.00 | 0.83 | 3 | Yes |
| primary_suspect_only | 1407 | 3153534 | Hepatobiliary disorders | 255 | 77914 | 8.74 (7.63-10.01) | 7.34 | 1426.09 | 2.83 | 2.64 | 3 | Yes |
| primary_suspect_only | 1407 | 3153534 | General disorders and administration site conditions | 444 | 1161805 | 0.79 (0.71-0.88) | 0.86 | 16.88 | -0.22 | -0.38 | 0 | No |
| primary_suspect_only | 1407 | 3153534 | Respiratory, thoracic and mediastinal disorders | 40 | 299269 | 0.28 (0.20-0.38) | 0.30 | 72.36 | -1.71 | -2.17 | 0 | No |
| primary_suspect_only | 1407 | 3153534 | Musculoskeletal and connective tissue disorders | 80 | 289676 | 0.60 (0.48-0.75) | 0.62 | 20.65 | -0.69 | -1.02 | 0 | No |
| primary_suspect_only | 1407 | 3153534 | Infections and infestations | 65 | 390576 | 0.34 (0.27-0.44) | 0.37 | 78.18 | -1.41 | -1.77 | 0 | No |
| primary_suspect_only | 1407 | 3153534 | Vascular disorders | 15 | 159911 | 0.20 (0.12-0.34) | 0.21 | 46.87 | -2.18 | -2.90 | 0 | No |
| primary_suspect_only | 1407 | 3153534 | Metabolism and nutrition disorders | 62 | 160622 | 0.86 (0.67-1.11) | 0.87 | 1.37 | -0.21 | -0.58 | 0 | No |
| primary_suspect_only | 1407 | 3153534 | Social circumstances | 10 | 46547 | 0.48 (0.26-0.89) | 0.48 | 5.67 | -0.99 | -1.86 | 0 | No |
| primary_suspect_only | 1407 | 3153534 | Renal and urinary disorders | 50 | 121194 | 0.92 (0.70-1.22) | 0.92 | 0.32 | -0.11 | -0.52 | 0 | No |
| primary_suspect_only | 1407 | 3153534 | Psychiatric disorders | 74 | 270660 | 0.59 (0.47-0.75) | 0.61 | 19.80 | -0.70 | -1.04 | 0 | No |
| primary_suspect_only | 1407 | 3153534 | Blood and lymphatic system disorders | 15 | 138429 | 0.23 (0.14-0.39) | 0.24 | 37.03 | -1.97 | -2.70 | 0 | No |
| primary_suspect_only | 1407 | 3153534 | Immune system disorders | 30 | 107490 | 0.62 (0.43-0.89) | 0.63 | 6.96 | -0.66 | -1.18 | 0 | No |
| primary_suspect_only | 1407 | 3153534 | Cardiac disorders | 19 | 144400 | 0.29 (0.18-0.45) | 0.29 | 33.56 | -1.71 | -2.36 | 0 | No |
| primary_suspect_only | 1407 | 3153534 | Neoplasms benign, malignant and unspecified (incl cysts and polyps) | 19 | 144181 | 0.29 (0.18-0.45) | 0.30 | 33.47 | -1.71 | -2.36 | 0 | No |
| primary_suspect_only | 1407 | 3153534 | Reproductive system and breast disorders | 8 | 47421 | 0.37 (0.19-0.75) | 0.38 | 8.31 | -1.30 | -2.27 | 0 | No |
| primary_suspect_only | 1407 | 3153534 | Ear and labyrinth disorders | 6 | 34761 | 0.38 (0.17-0.86) | 0.39 | 5.89 | -1.24 | -2.33 | 0 | No |
| primary_suspect_only | 1407 | 3153534 | Surgical and medical procedures | 76 | 155123 | 1.10 (0.88-1.39) | 1.10 | 0.70 | 0.13 | -0.21 | 0 | No |
| primary_suspect_only | 1407 | 3153534 | Product issues | 4 | 184346 | 0.05 (0.02-0.12) | 0.05 | 79.06 | -4.06 | -5.35 | 0 | No |
| primary_suspect_only | 1407 | 3153534 | Eye disorders | 30 | 147964 | 0.44 (0.31-0.64) | 0.45 | 20.61 | -1.11 | -1.64 | 0 | No |
| primary_suspect_only | 1407 | 3153534 | Endocrine disorders | 5 | 26342 | 0.42 (0.18-1.02) | 0.43 | 3.91 | -1.09 | -2.27 | 0 | No |
| ps_ss_interacting | 1429 | 3153534 | Investigations | 438 | 351080 | 3.53 (3.15-3.95) | 2.75 | 549.60 | 1.45 | 1.30 | 3 | Yes |
| ps_ss_interacting | 1429 | 3153534 | Nervous system disorders | 217 | 479908 | 1.00 (0.86-1.15) | 1.00 | 0.00 | -0.00 | -0.21 | 0 | No |
| ps_ss_interacting | 1429 | 3153534 | Injury, poisoning and procedural complications | 134 | 997506 | 0.22 (0.19-0.27) | 0.30 | 327.16 | -1.75 | -2.01 | 0 | No |
| ps_ss_interacting | 1429 | 3153534 | Gastrointestinal disorders | 548 | 479527 | 3.47 (3.12-3.86) | 2.52 | 592.96 | 1.33 | 1.18 | 3 | Yes |
| ps_ss_interacting | 1429 | 3153534 | Skin and subcutaneous tissue disorders | 342 | 370802 | 2.36 (2.09-2.67) | 2.04 | 203.96 | 1.02 | 0.85 | 3 | Yes |
| ps_ss_interacting | 1429 | 3153534 | Hepatobiliary disorders | 258 | 77914 | 8.70 (7.60-9.95) | 7.31 | 1435.58 | 2.83 | 2.63 | 3 | Yes |
| ps_ss_interacting | 1429 | 3153534 | General disorders and administration site conditions | 457 | 1161805 | 0.81 (0.72-0.90) | 0.87 | 14.51 | -0.20 | -0.36 | 0 | No |
| ps_ss_interacting | 1429 | 3153534 | Respiratory, thoracic and mediastinal disorders | 43 | 299269 | 0.30 (0.22-0.40) | 0.32 | 69.87 | -1.64 | -2.08 | 0 | No |
| ps_ss_interacting | 1429 | 3153534 | Musculoskeletal and connective tissue disorders | 86 | 289676 | 0.63 (0.51-0.79) | 0.66 | 17.18 | -0.61 | -0.92 | 0 | No |
| ps_ss_interacting | 1429 | 3153534 | Infections and infestations | 68 | 390576 | 0.35 (0.28-0.45) | 0.38 | 76.58 | -1.37 | -1.72 | 0 | No |
| ps_ss_interacting | 1429 | 3153534 | Vascular disorders | 18 | 159911 | 0.24 (0.15-0.38) | 0.25 | 43.11 | -1.95 | -2.62 | 0 | No |
| ps_ss_interacting | 1429 | 3153534 | Metabolism and nutrition disorders | 64 | 160622 | 0.87 (0.68-1.12) | 0.88 | 1.12 | -0.18 | -0.55 | 0 | No |
| ps_ss_interacting | 1429 | 3153534 | Social circumstances | 11 | 46547 | 0.52 (0.29-0.94) | 0.52 | 4.90 | -0.88 | -1.72 | 0 | No |
| ps_ss_interacting | 1429 | 3153534 | Renal and urinary disorders | 52 | 121194 | 0.94 (0.72-1.25) | 0.95 | 0.16 | -0.08 | -0.48 | 0 | No |
| ps_ss_interacting | 1429 | 3153534 | Psychiatric disorders | 77 | 270660 | 0.61 (0.48-0.76) | 0.63 | 18.58 | -0.67 | -1.00 | 0 | No |
| ps_ss_interacting | 1429 | 3153534 | Blood and lymphatic system disorders | 16 | 138429 | 0.25 (0.15-0.40) | 0.26 | 36.40 | -1.91 | -2.61 | 0 | No |
| ps_ss_interacting | 1429 | 3153534 | Immune system disorders | 33 | 107490 | 0.67 (0.47-0.95) | 0.68 | 5.24 | -0.55 | -1.05 | 0 | No |
| ps_ss_interacting | 1429 | 3153534 | Cardiac disorders | 19 | 144400 | 0.28 (0.18-0.44) | 0.29 | 34.53 | -1.73 | -2.38 | 0 | No |
| ps_ss_interacting | 1429 | 3153534 | Neoplasms benign, malignant and unspecified (incl cysts and polyps) | 20 | 144181 | 0.30 (0.19-0.46) | 0.31 | 32.96 | -1.66 | -2.29 | 0 | No |
| ps_ss_interacting | 1429 | 3153534 | Reproductive system and breast disorders | 8 | 47421 | 0.37 (0.18-0.74) | 0.37 | 8.59 | -1.32 | -2.29 | 0 | No |
| ps_ss_interacting | 1429 | 3153534 | Ear and labyrinth disorders | 8 | 34761 | 0.51 (0.25-1.01) | 0.51 | 3.86 | -0.90 | -1.86 | 0 | No |
| ps_ss_interacting | 1429 | 3153534 | Surgical and medical procedures | 77 | 155123 | 1.10 (0.87-1.39) | 1.10 | 0.67 | 0.13 | -0.21 | 0 | No |
| ps_ss_interacting | 1429 | 3153534 | Product issues | 4 | 184346 | 0.05 (0.02-0.12) | 0.05 | 80.42 | -4.08 | -5.37 | 0 | No |
| ps_ss_interacting | 1429 | 3153534 | Eye disorders | 32 | 147964 | 0.47 (0.33-0.66) | 0.48 | 19.22 | -1.04 | -1.55 | 0 | No |
| ps_ss_interacting | 1429 | 3153534 | Endocrine disorders | 5 | 26342 | 0.42 (0.17-1.00) | 0.42 | 4.06 | -1.11 | -2.29 | 0 | No |
| ps_ss_interacting | 1429 | 3153534 | Congenital, familial and genetic disorders | 2 | 18671 | 0.24 (0.06-0.94) | 0.24 | 4.96 | -1.66 | -3.33 | 0 | No |
| all_roles_exploratory | 1519 | 3153534 | Investigations | 457 | 351080 | 3.43 (3.08-3.83) | 2.70 | 550.84 | 1.43 | 1.27 | 3 | Yes |
| all_roles_exploratory | 1519 | 3153534 | Nervous system disorders | 232 | 479908 | 1.00 (0.87-1.15) | 1.00 | 0.00 | 0.00 | -0.20 | 0 | No |
| all_roles_exploratory | 1519 | 3153534 | Injury, poisoning and procedural complications | 152 | 997506 | 0.24 (0.20-0.28) | 0.32 | 328.36 | -1.65 | -1.90 | 0 | No |
| all_roles_exploratory | 1519 | 3153534 | Gastrointestinal disorders | 571 | 479527 | 3.36 (3.03-3.73) | 2.47 | 589.67 | 1.30 | 1.16 | 3 | Yes |
| all_roles_exploratory | 1519 | 3153534 | Skin and subcutaneous tissue disorders | 362 | 370802 | 2.35 (2.09-2.64) | 2.03 | 213.20 | 1.01 | 0.84 | 3 | Yes |
| all_roles_exploratory | 1519 | 3153534 | Hepatobiliary disorders | 262 | 77914 | 8.23 (7.20-9.40) | 6.98 | 1372.08 | 2.77 | 2.57 | 3 | Yes |
| all_roles_exploratory | 1519 | 3153534 | General disorders and administration site conditions | 494 | 1161805 | 0.83 (0.74-0.92) | 0.88 | 12.18 | -0.18 | -0.33 | 0 | No |
| all_roles_exploratory | 1519 | 3153534 | Respiratory, thoracic and mediastinal disorders | 49 | 299269 | 0.32 (0.24-0.42) | 0.34 | 69.38 | -1.54 | -1.95 | 0 | No |
| all_roles_exploratory | 1519 | 3153534 | Musculoskeletal and connective tissue disorders | 98 | 289676 | 0.68 (0.56-0.84) | 0.70 | 13.61 | -0.51 | -0.81 | 0 | No |
| all_roles_exploratory | 1519 | 3153534 | Infections and infestations | 79 | 390576 | 0.39 (0.31-0.49) | 0.42 | 72.24 | -1.24 | -1.57 | 0 | No |
| all_roles_exploratory | 1519 | 3153534 | Vascular disorders | 22 | 159911 | 0.28 (0.18-0.42) | 0.29 | 41.40 | -1.76 | -2.37 | 0 | No |
| all_roles_exploratory | 1519 | 3153534 | Metabolism and nutrition disorders | 68 | 160622 | 0.87 (0.68-1.11) | 0.88 | 1.19 | -0.18 | -0.54 | 0 | No |
| all_roles_exploratory | 1519 | 3153534 | Social circumstances | 14 | 46547 | 0.62 (0.37-1.05) | 0.62 | 3.21 | -0.64 | -1.39 | 0 | No |
| all_roles_exploratory | 1519 | 3153534 | Renal and urinary disorders | 53 | 121194 | 0.90 (0.69-1.19) | 0.91 | 0.51 | -0.14 | -0.54 | 0 | No |
| all_roles_exploratory | 1519 | 3153534 | Psychiatric disorders | 83 | 270660 | 0.62 (0.49-0.77) | 0.64 | 18.82 | -0.65 | -0.97 | 0 | No |
| all_roles_exploratory | 1519 | 3153534 | Blood and lymphatic system disorders | 16 | 138429 | 0.23 (0.14-0.38) | 0.24 | 40.28 | -1.99 | -2.70 | 0 | No |
| all_roles_exploratory | 1519 | 3153534 | Immune system disorders | 36 | 107490 | 0.69 (0.49-0.96) | 0.70 | 4.97 | -0.51 | -0.99 | 0 | No |
| all_roles_exploratory | 1519 | 3153534 | Cardiac disorders | 23 | 144400 | 0.32 (0.21-0.48) | 0.33 | 32.65 | -1.56 | -2.15 | 0 | No |
| all_roles_exploratory | 1519 | 3153534 | Neoplasms benign, malignant and unspecified (incl cysts and polyps) | 21 | 144181 | 0.29 (0.19-0.45) | 0.30 | 35.41 | -1.68 | -2.30 | 0 | No |
| all_roles_exploratory | 1519 | 3153534 | Reproductive system and breast disorders | 8 | 47421 | 0.35 (0.17-0.69) | 0.35 | 9.79 | -1.41 | -2.37 | 0 | No |
| all_roles_exploratory | 1519 | 3153534 | Ear and labyrinth disorders | 8 | 34761 | 0.48 (0.24-0.95) | 0.48 | 4.62 | -0.98 | -1.94 | 0 | No |
| all_roles_exploratory | 1519 | 3153534 | Surgical and medical procedures | 86 | 155123 | 1.16 (0.93-1.44) | 1.15 | 1.79 | 0.20 | -0.12 | 0 | No |
| all_roles_exploratory | 1519 | 3153534 | Product issues | 5 | 184346 | 0.05 (0.02-0.13) | 0.06 | 83.98 | -3.90 | -5.08 | 0 | No |
| all_roles_exploratory | 1519 | 3153534 | Eye disorders | 38 | 147964 | 0.52 (0.38-0.72) | 0.53 | 16.29 | -0.89 | -1.36 | 0 | No |
| all_roles_exploratory | 1519 | 3153534 | Endocrine disorders | 5 | 26342 | 0.39 (0.16-0.94) | 0.39 | 4.70 | -1.19 | -2.37 | 0 | No |
| all_roles_exploratory | 1519 | 3153534 | Congenital, familial and genetic disorders | 2 | 18671 | 0.22 (0.06-0.89) | 0.22 | 5.47 | -1.74 | -3.40 | 0 | No |
| exclude_glp1_gip | 1266 | 2961107 | Investigations | 363 | 326929 | 3.24 (2.87-3.66) | 2.60 | 400.32 | 1.37 | 1.20 | 3 | Yes |
| exclude_glp1_gip | 1266 | 2961107 | Nervous system disorders | 187 | 458822 | 0.95 (0.81-1.10) | 0.95 | 0.51 | -0.07 | -0.30 | 0 | No |
| exclude_glp1_gip | 1266 | 2961107 | Injury, poisoning and procedural complications | 108 | 921950 | 0.21 (0.17-0.25) | 0.27 | 301.62 | -1.86 | -2.15 | 0 | No |
| exclude_glp1_gip | 1266 | 2961107 | Gastrointestinal disorders | 473 | 427250 | 3.54 (3.16-3.96) | 2.59 | 538.73 | 1.37 | 1.21 | 3 | Yes |
| exclude_glp1_gip | 1266 | 2961107 | Skin and subcutaneous tissue disorders | 307 | 359307 | 2.32 (2.04-2.64) | 2.00 | 174.15 | 0.99 | 0.81 | 2 | No |
| exclude_glp1_gip | 1266 | 2961107 | Hepatobiliary disorders | 219 | 74114 | 8.15 (7.04-9.43) | 6.91 | 1132.43 | 2.75 | 2.54 | 3 | Yes |
| exclude_glp1_gip | 1266 | 2961107 | General disorders and administration site conditions | 396 | 1093431 | 0.78 (0.69-0.88) | 0.85 | 17.33 | -0.24 | -0.41 | 0 | No |
| exclude_glp1_gip | 1266 | 2961107 | Respiratory, thoracic and mediastinal disorders | 35 | 290941 | 0.26 (0.19-0.37) | 0.28 | 71.23 | -1.80 | -2.29 | 0 | No |
| exclude_glp1_gip | 1266 | 2961107 | Musculoskeletal and connective tissue disorders | 71 | 279175 | 0.57 (0.45-0.73) | 0.59 | 21.63 | -0.74 | -1.09 | 0 | No |
| exclude_glp1_gip | 1266 | 2961107 | Infections and infestations | 55 | 379436 | 0.31 (0.24-0.40) | 0.34 | 81.27 | -1.54 | -1.94 | 0 | No |
| exclude_glp1_gip | 1266 | 2961107 | Vascular disorders | 15 | 154983 | 0.22 (0.13-0.36) | 0.23 | 41.84 | -2.07 | -2.80 | 0 | No |
| exclude_glp1_gip | 1266 | 2961107 | Metabolism and nutrition disorders | 51 | 142680 | 0.83 (0.63-1.10) | 0.84 | 1.72 | -0.25 | -0.66 | 0 | No |
| exclude_glp1_gip | 1266 | 2961107 | Social circumstances | 11 | 44777 | 0.57 (0.32-1.03) | 0.57 | 3.52 | -0.75 | -1.59 | 0 | No |
| exclude_glp1_gip | 1266 | 2961107 | Renal and urinary disorders | 43 | 116301 | 0.86 (0.63-1.17) | 0.86 | 0.95 | -0.21 | -0.65 | 0 | No |
| exclude_glp1_gip | 1266 | 2961107 | Psychiatric disorders | 63 | 258744 | 0.55 (0.42-0.70) | 0.57 | 22.46 | -0.80 | -1.17 | 0 | No |
| exclude_glp1_gip | 1266 | 2961107 | Blood and lymphatic system disorders | 11 | 136932 | 0.18 (0.10-0.33) | 0.19 | 40.48 | -2.31 | -3.15 | 0 | No |
| exclude_glp1_gip | 1266 | 2961107 | Immune system disorders | 31 | 104291 | 0.69 (0.48-0.98) | 0.70 | 4.29 | -0.51 | -1.03 | 0 | No |
| exclude_glp1_gip | 1266 | 2961107 | Cardiac disorders | 18 | 139253 | 0.29 (0.18-0.47) | 0.30 | 30.40 | -1.67 | -2.34 | 0 | No |
| exclude_glp1_gip | 1266 | 2961107 | Neoplasms benign, malignant and unspecified (incl cysts and polyps) | 19 | 140859 | 0.31 (0.19-0.48) | 0.32 | 29.62 | -1.61 | -2.27 | 0 | No |
| exclude_glp1_gip | 1266 | 2961107 | Reproductive system and breast disorders | 6 | 45507 | 0.31 (0.14-0.68) | 0.31 | 9.45 | -1.55 | -2.64 | 0 | No |
| exclude_glp1_gip | 1266 | 2961107 | Ear and labyrinth disorders | 5 | 33005 | 0.35 (0.15-0.85) | 0.35 | 5.95 | -1.33 | -2.51 | 0 | No |
| exclude_glp1_gip | 1266 | 2961107 | Surgical and medical procedures | 59 | 149953 | 0.92 (0.71-1.19) | 0.92 | 0.43 | -0.12 | -0.50 | 0 | No |
| exclude_glp1_gip | 1266 | 2961107 | Product issues | 4 | 180276 | 0.05 (0.02-0.13) | 0.05 | 73.77 | -3.97 | -5.26 | 0 | No |
| exclude_glp1_gip | 1266 | 2961107 | Eye disorders | 29 | 139338 | 0.47 (0.33-0.69) | 0.49 | 16.46 | -1.01 | -1.55 | 0 | No |
| exclude_glp1_gip | 1266 | 2961107 | Endocrine disorders | 3 | 25200 | 0.28 (0.09-0.86) | 0.28 | 5.66 | -1.56 | -3.00 | 0 | No |
| exclude_statin | 1162 | 2993835 | Investigations | 321 | 321351 | 3.17 (2.79-3.61) | 2.57 | 345.68 | 1.35 | 1.17 | 3 | Yes |
| exclude_statin | 1162 | 2993835 | Nervous system disorders | 170 | 443288 | 0.99 (0.84-1.16) | 0.99 | 0.03 | -0.02 | -0.25 | 0 | No |
| exclude_statin | 1162 | 2993835 | Injury, poisoning and procedural complications | 99 | 954713 | 0.20 (0.16-0.24) | 0.27 | 292.11 | -1.89 | -2.20 | 0 | No |
| exclude_statin | 1162 | 2993835 | Gastrointestinal disorders | 434 | 441964 | 3.44 (3.06-3.88) | 2.53 | 470.71 | 1.33 | 1.17 | 3 | Yes |
| exclude_statin | 1162 | 2993835 | Skin and subcutaneous tissue disorders | 281 | 349564 | 2.41 (2.11-2.76) | 2.07 | 176.10 | 1.04 | 0.85 | 3 | Yes |
| exclude_statin | 1162 | 2993835 | Hepatobiliary disorders | 202 | 71190 | 8.64 (7.42-10.06) | 7.31 | 1124.06 | 2.82 | 2.60 | 3 | Yes |
| exclude_statin | 1162 | 2993835 | General disorders and administration site conditions | 357 | 1099661 | 0.76 (0.67-0.87) | 0.84 | 18.04 | -0.26 | -0.43 | 0 | No |
| exclude_statin | 1162 | 2993835 | Infections and infestations | 35 | 362718 | 0.23 (0.16-0.32) | 0.25 | 90.43 | -1.98 | -2.47 | 0 | No |
| exclude_statin | 1162 | 2993835 | Vascular disorders | 11 | 144207 | 0.19 (0.10-0.34) | 0.20 | 37.96 | -2.25 | -3.09 | 0 | No |
| exclude_statin | 1162 | 2993835 | Metabolism and nutrition disorders | 49 | 143545 | 0.87 (0.66-1.16) | 0.88 | 0.85 | -0.18 | -0.60 | 0 | No |
| exclude_statin | 1162 | 2993835 | Social circumstances | 6 | 43149 | 0.35 (0.16-0.79) | 0.36 | 7.00 | -1.34 | -2.44 | 0 | No |
| exclude_statin | 1162 | 2993835 | Renal and urinary disorders | 34 | 107571 | 0.81 (0.57-1.14) | 0.81 | 1.49 | -0.29 | -0.78 | 0 | No |
| exclude_statin | 1162 | 2993835 | Psychiatric disorders | 50 | 252829 | 0.49 (0.37-0.65) | 0.51 | 25.78 | -0.96 | -1.37 | 0 | No |
| exclude_statin | 1162 | 2993835 | Musculoskeletal and connective tissue disorders | 51 | 260662 | 0.48 (0.36-0.64) | 0.50 | 27.25 | -0.98 | -1.38 | 0 | No |
| exclude_statin | 1162 | 2993835 | Immune system disorders | 28 | 100767 | 0.71 (0.49-1.03) | 0.72 | 3.27 | -0.47 | -1.01 | 0 | No |
| exclude_statin | 1162 | 2993835 | Reproductive system and breast disorders | 8 | 44504 | 0.46 (0.23-0.92) | 0.46 | 5.05 | -1.02 | -1.99 | 0 | No |
| exclude_statin | 1162 | 2993835 | Respiratory, thoracic and mediastinal disorders | 23 | 273272 | 0.20 (0.13-0.30) | 0.22 | 71.58 | -2.16 | -2.75 | 0 | No |
| exclude_statin | 1162 | 2993835 | Ear and labyrinth disorders | 6 | 31469 | 0.49 (0.22-1.09) | 0.49 | 3.19 | -0.92 | -2.01 | 0 | No |
| exclude_statin | 1162 | 2993835 | Surgical and medical procedures | 52 | 146594 | 0.91 (0.69-1.20) | 0.91 | 0.44 | -0.13 | -0.53 | 0 | No |
| exclude_statin | 1162 | 2993835 | Product issues | 2 | 179275 | 0.03 (0.01-0.11) | 0.03 | 69.82 | -4.56 | -6.23 | 0 | No |
| exclude_statin | 1162 | 2993835 | Blood and lymphatic system disorders | 9 | 129685 | 0.17 (0.09-0.33) | 0.18 | 35.48 | -2.36 | -3.28 | 0 | No |
| exclude_statin | 1162 | 2993835 | Eye disorders | 21 | 137312 | 0.38 (0.25-0.59) | 0.39 | 20.51 | -1.30 | -1.93 | 0 | No |
| exclude_statin | 1162 | 2993835 | Cardiac disorders | 13 | 129902 | 0.25 (0.14-0.43) | 0.26 | 29.03 | -1.88 | -2.65 | 0 | No |
| exclude_statin | 1162 | 2993835 | Endocrine disorders | 2 | 24175 | 0.21 (0.05-0.85) | 0.21 | 5.86 | -1.79 | -3.46 | 0 | No |
| exclude_statin | 1162 | 2993835 | Congenital, familial and genetic disorders | 2 | 17838 | 0.29 (0.07-1.15) | 0.29 | 3.52 | -1.40 | -3.07 | 0 | No |
| exclude_statin | 1162 | 2993835 | Neoplasms benign, malignant and unspecified (incl cysts and polyps) | 12 | 136911 | 0.22 (0.12-0.38) | 0.23 | 33.37 | -2.06 | -2.86 | 0 | No |
| exclude_hepatotoxic_drugs | 1350 | 2824038 | Investigations | 408 | 294037 | 3.73 (3.32-4.19) | 2.90 | 567.25 | 1.53 | 1.37 | 3 | Yes |
| exclude_hepatotoxic_drugs | 1350 | 2824038 | Nervous system disorders | 196 | 418150 | 0.98 (0.84-1.14) | 0.98 | 0.09 | -0.03 | -0.25 | 0 | No |
| exclude_hepatotoxic_drugs | 1350 | 2824038 | Injury, poisoning and procedural complications | 127 | 896939 | 0.22 (0.19-0.27) | 0.30 | 311.15 | -1.75 | -2.01 | 0 | No |
| exclude_hepatotoxic_drugs | 1350 | 2824038 | Gastrointestinal disorders | 510 | 406182 | 3.61 (3.24-4.03) | 2.63 | 599.34 | 1.39 | 1.24 | 3 | Yes |
| exclude_hepatotoxic_drugs | 1350 | 2824038 | Skin and subcutaneous tissue disorders | 320 | 320764 | 2.42 (2.14-2.75) | 2.09 | 204.17 | 1.05 | 0.88 | 3 | Yes |
| exclude_hepatotoxic_drugs | 1350 | 2824038 | Hepatobiliary disorders | 236 | 54370 | 10.79 (9.38-12.42) | 9.08 | 1722.85 | 3.13 | 2.92 | 3 | Yes |
| exclude_hepatotoxic_drugs | 1350 | 2824038 | General disorders and administration site conditions | 422 | 1024080 | 0.80 (0.71-0.90) | 0.86 | 14.62 | -0.21 | -0.38 | 0 | No |
| exclude_hepatotoxic_drugs | 1350 | 2824038 | Respiratory, thoracic and mediastinal disorders | 37 | 251330 | 0.29 (0.21-0.40) | 0.31 | 63.15 | -1.67 | -2.15 | 0 | No |
| exclude_hepatotoxic_drugs | 1350 | 2824038 | Musculoskeletal and connective tissue disorders | 80 | 243207 | 0.67 (0.53-0.84) | 0.69 | 12.37 | -0.53 | -0.86 | 0 | No |
| exclude_hepatotoxic_drugs | 1350 | 2824038 | Infections and infestations | 59 | 318984 | 0.36 (0.28-0.47) | 0.39 | 64.60 | -1.36 | -1.74 | 0 | No |
| exclude_hepatotoxic_drugs | 1350 | 2824038 | Vascular disorders | 15 | 132131 | 0.23 (0.14-0.38) | 0.24 | 38.52 | -2.00 | -2.73 | 0 | No |
| exclude_hepatotoxic_drugs | 1350 | 2824038 | Metabolism and nutrition disorders | 57 | 131370 | 0.90 (0.69-1.18) | 0.91 | 0.56 | -0.14 | -0.53 | 0 | No |
| exclude_hepatotoxic_drugs | 1350 | 2824038 | Social circumstances | 7 | 41624 | 0.35 (0.17-0.73) | 0.35 | 8.48 | -1.39 | -2.41 | 0 | No |
| exclude_hepatotoxic_drugs | 1350 | 2824038 | Renal and urinary disorders | 41 | 97332 | 0.88 (0.64-1.20) | 0.88 | 0.68 | -0.18 | -0.63 | 0 | No |
| exclude_hepatotoxic_drugs | 1350 | 2824038 | Psychiatric disorders | 68 | 243547 | 0.56 (0.44-0.72) | 0.58 | 22.04 | -0.77 | -1.12 | 0 | No |
| exclude_hepatotoxic_drugs | 1350 | 2824038 | Blood and lymphatic system disorders | 15 | 96069 | 0.32 (0.19-0.53) | 0.33 | 21.55 | -1.55 | -2.28 | 0 | No |
| exclude_hepatotoxic_drugs | 1350 | 2824038 | Immune system disorders | 28 | 84241 | 0.69 (0.47-1.00) | 0.70 | 3.85 | -0.51 | -1.05 | 0 | No |
| exclude_hepatotoxic_drugs | 1350 | 2824038 | Cardiac disorders | 17 | 118635 | 0.29 (0.18-0.47) | 0.30 | 29.02 | -1.68 | -2.37 | 0 | No |
| exclude_hepatotoxic_drugs | 1350 | 2824038 | Neoplasms benign, malignant and unspecified (incl cysts and polyps) | 19 | 111790 | 0.35 (0.22-0.54) | 0.36 | 23.11 | -1.45 | -2.10 | 0 | No |
| exclude_hepatotoxic_drugs | 1350 | 2824038 | Reproductive system and breast disorders | 8 | 42995 | 0.39 (0.19-0.77) | 0.39 | 7.78 | -1.26 | -2.23 | 0 | No |
| exclude_hepatotoxic_drugs | 1350 | 2824038 | Ear and labyrinth disorders | 8 | 30269 | 0.55 (0.27-1.10) | 0.55 | 2.92 | -0.78 | -1.75 | 0 | No |
| exclude_hepatotoxic_drugs | 1350 | 2824038 | Surgical and medical procedures | 75 | 140918 | 1.12 (0.89-1.41) | 1.11 | 0.91 | 0.15 | -0.19 | 0 | No |
| exclude_hepatotoxic_drugs | 1350 | 2824038 | Product issues | 4 | 178664 | 0.04 (0.02-0.12) | 0.05 | 82.83 | -4.11 | -5.40 | 0 | No |
| exclude_hepatotoxic_drugs | 1350 | 2824038 | Eye disorders | 27 | 132540 | 0.41 (0.28-0.61) | 0.43 | 21.89 | -1.20 | -1.75 | 0 | No |
| exclude_hepatotoxic_drugs | 1350 | 2824038 | Endocrine disorders | 4 | 15717 | 0.53 (0.20-1.42) | 0.53 | 1.65 | -0.77 | -2.06 | 0 | No |
| metabolic_background_reference | 1429 | 340363 | Investigations | 438 | 53429 | 2.37 (2.12-2.66) | 1.95 | 239.66 | 0.96 | 0.80 | 2 | No |
| metabolic_background_reference | 1429 | 340363 | Nervous system disorders | 217 | 46714 | 1.13 (0.97-1.30) | 1.11 | 2.56 | 0.14 | -0.07 | 0 | No |
| metabolic_background_reference | 1429 | 340363 | Injury, poisoning and procedural complications | 134 | 124391 | 0.18 (0.15-0.21) | 0.26 | 453.57 | -1.95 | -2.21 | 0 | No |
| metabolic_background_reference | 1429 | 340363 | Gastrointestinal disorders | 548 | 74772 | 2.21 (1.99-2.46) | 1.75 | 222.23 | 0.80 | 0.65 | 2 | No |
| metabolic_background_reference | 1429 | 340363 | Skin and subcutaneous tissue disorders | 342 | 24680 | 4.02 (3.56-4.55) | 3.30 | 583.65 | 1.70 | 1.52 | 3 | Yes |
| metabolic_background_reference | 1429 | 340363 | Hepatobiliary disorders | 258 | 7658 | 9.57 (8.35-10.97) | 8.02 | 1571.14 | 2.92 | 2.73 | 3 | Yes |
| metabolic_background_reference | 1429 | 340363 | General disorders and administration site conditions | 457 | 115145 | 0.92 (0.82-1.03) | 0.95 | 2.18 | -0.08 | -0.24 | 0 | No |
| metabolic_background_reference | 1429 | 340363 | Respiratory, thoracic and mediastinal disorders | 43 | 23567 | 0.42 (0.31-0.57) | 0.43 | 33.92 | -1.18 | -1.62 | 0 | No |
| metabolic_background_reference | 1429 | 340363 | Musculoskeletal and connective tissue disorders | 86 | 24152 | 0.84 (0.67-1.04) | 0.85 | 2.51 | -0.24 | -0.55 | 0 | No |
| metabolic_background_reference | 1429 | 340363 | Infections and infestations | 68 | 31177 | 0.50 (0.39-0.63) | 0.52 | 33.19 | -0.93 | -1.29 | 0 | No |
| metabolic_background_reference | 1429 | 340363 | Vascular disorders | 18 | 17335 | 0.24 (0.15-0.38) | 0.25 | 43.39 | -1.95 | -2.62 | 0 | No |
| metabolic_background_reference | 1429 | 340363 | Metabolism and nutrition disorders | 64 | 40254 | 0.35 (0.27-0.45) | 0.38 | 73.85 | -1.38 | -1.75 | 0 | No |
| metabolic_background_reference | 1429 | 340363 | Social circumstances | 11 | 3953 | 0.66 (0.36-1.20) | 0.66 | 1.90 | -0.55 | -1.39 | 0 | No |
| metabolic_background_reference | 1429 | 340363 | Renal and urinary disorders | 52 | 17913 | 0.68 (0.52-0.90) | 0.69 | 7.54 | -0.52 | -0.93 | 0 | No |
| metabolic_background_reference | 1429 | 340363 | Psychiatric disorders | 77 | 23513 | 0.77 (0.61-0.97) | 0.78 | 5.12 | -0.35 | -0.69 | 0 | No |
| metabolic_background_reference | 1429 | 340363 | Blood and lymphatic system disorders | 16 | 7267 | 0.52 (0.32-0.85) | 0.52 | 7.04 | -0.89 | -1.59 | 0 | No |
| metabolic_background_reference | 1429 | 340363 | Immune system disorders | 33 | 6973 | 1.13 (0.80-1.60) | 1.13 | 0.48 | 0.17 | -0.34 | 0 | No |
| metabolic_background_reference | 1429 | 340363 | Cardiac disorders | 19 | 17303 | 0.25 (0.16-0.40) | 0.26 | 41.68 | -1.88 | -2.53 | 0 | No |
| metabolic_background_reference | 1429 | 340363 | Neoplasms benign, malignant and unspecified (incl cysts and polyps) | 20 | 8742 | 0.54 (0.35-0.84) | 0.54 | 7.78 | -0.84 | -1.48 | 0 | No |
| metabolic_background_reference | 1429 | 340363 | Reproductive system and breast disorders | 8 | 4099 | 0.46 (0.23-0.93) | 0.46 | 4.98 | -1.01 | -1.98 | 0 | No |
| metabolic_background_reference | 1429 | 340363 | Ear and labyrinth disorders | 8 | 4475 | 0.42 (0.21-0.85) | 0.43 | 6.27 | -1.13 | -2.10 | 0 | No |
| metabolic_background_reference | 1429 | 340363 | Surgical and medical procedures | 77 | 11714 | 1.60 (1.27-2.01) | 1.57 | 16.19 | 0.63 | 0.30 | 2 | No |
| metabolic_background_reference | 1429 | 340363 | Product issues | 4 | 10497 | 0.09 (0.03-0.24) | 0.09 | 37.57 | -3.17 | -4.46 | 0 | No |
| metabolic_background_reference | 1429 | 340363 | Eye disorders | 32 | 18125 | 0.41 (0.29-0.58) | 0.42 | 26.94 | -1.22 | -1.73 | 0 | No |
| metabolic_background_reference | 1429 | 340363 | Endocrine disorders | 5 | 2691 | 0.44 (0.18-1.06) | 0.44 | 3.53 | -1.03 | -2.22 | 0 | No |
| metabolic_background_reference | 1429 | 340363 | Congenital, familial and genetic disorders | 2 | 1314 | 0.36 (0.09-1.45) | 0.36 | 2.25 | -1.12 | -2.79 | 0 | No |
| exclude_target_cirrhosis_indication | 1394 | 3153534 | Investigations | 429 | 351080 | 3.55 (3.17-3.98) | 2.76 | 542.99 | 1.46 | 1.30 | 3 | Yes |
| exclude_target_cirrhosis_indication | 1394 | 3153534 | Nervous system disorders | 210 | 479908 | 0.99 (0.85-1.14) | 0.99 | 0.03 | -0.02 | -0.23 | 0 | No |
| exclude_target_cirrhosis_indication | 1394 | 3153534 | Injury, poisoning and procedural complications | 101 | 997506 | 0.17 (0.14-0.21) | 0.23 | 383.23 | -2.12 | -2.41 | 0 | No |
| exclude_target_cirrhosis_indication | 1394 | 3153534 | Gastrointestinal disorders | 538 | 479527 | 3.50 (3.15-3.90) | 2.54 | 590.79 | 1.34 | 1.19 | 3 | Yes |
| exclude_target_cirrhosis_indication | 1394 | 3153534 | Skin and subcutaneous tissue disorders | 337 | 370802 | 2.39 (2.12-2.70) | 2.06 | 206.96 | 1.03 | 0.86 | 3 | Yes |
| exclude_target_cirrhosis_indication | 1394 | 3153534 | Hepatobiliary disorders | 245 | 77914 | 8.42 (7.33-9.66) | 7.11 | 1315.82 | 2.79 | 2.59 | 3 | Yes |
| exclude_target_cirrhosis_indication | 1394 | 3153534 | General disorders and administration site conditions | 436 | 1161805 | 0.78 (0.70-0.87) | 0.85 | 18.54 | -0.24 | -0.39 | 0 | No |
| exclude_target_cirrhosis_indication | 1394 | 3153534 | Respiratory, thoracic and mediastinal disorders | 42 | 299269 | 0.30 (0.22-0.40) | 0.32 | 68.07 | -1.63 | -2.08 | 0 | No |
| exclude_target_cirrhosis_indication | 1394 | 3153534 | Musculoskeletal and connective tissue disorders | 85 | 289676 | 0.64 (0.52-0.80) | 0.66 | 15.93 | -0.59 | -0.91 | 0 | No |
| exclude_target_cirrhosis_indication | 1394 | 3153534 | Infections and infestations | 67 | 390576 | 0.36 (0.28-0.46) | 0.39 | 73.78 | -1.35 | -1.71 | 0 | No |
| exclude_target_cirrhosis_indication | 1394 | 3153534 | Vascular disorders | 18 | 159911 | 0.24 (0.15-0.39) | 0.25 | 41.36 | -1.92 | -2.58 | 0 | No |
| exclude_target_cirrhosis_indication | 1394 | 3153534 | Metabolism and nutrition disorders | 64 | 160622 | 0.90 (0.70-1.15) | 0.90 | 0.73 | -0.15 | -0.51 | 0 | No |
| exclude_target_cirrhosis_indication | 1394 | 3153534 | Social circumstances | 11 | 46547 | 0.53 (0.29-0.96) | 0.53 | 4.52 | -0.85 | -1.68 | 0 | No |
| exclude_target_cirrhosis_indication | 1394 | 3153534 | Renal and urinary disorders | 51 | 121194 | 0.95 (0.72-1.26) | 0.95 | 0.13 | -0.07 | -0.48 | 0 | No |
| exclude_target_cirrhosis_indication | 1394 | 3153534 | Psychiatric disorders | 75 | 270660 | 0.61 (0.48-0.76) | 0.63 | 18.22 | -0.67 | -1.01 | 0 | No |
| exclude_target_cirrhosis_indication | 1394 | 3153534 | Blood and lymphatic system disorders | 13 | 138429 | 0.21 (0.12-0.35) | 0.21 | 39.69 | -2.15 | -2.93 | 0 | No |
| exclude_target_cirrhosis_indication | 1394 | 3153534 | Immune system disorders | 33 | 107490 | 0.69 (0.49-0.97) | 0.69 | 4.59 | -0.51 | -1.01 | 0 | No |
| exclude_target_cirrhosis_indication | 1394 | 3153534 | Cardiac disorders | 19 | 144400 | 0.29 (0.18-0.45) | 0.30 | 32.99 | -1.70 | -2.35 | 0 | No |
| exclude_target_cirrhosis_indication | 1394 | 3153534 | Neoplasms benign, malignant and unspecified (incl cysts and polyps) | 20 | 144181 | 0.30 (0.20-0.47) | 0.31 | 31.44 | -1.62 | -2.26 | 0 | No |
| exclude_target_cirrhosis_indication | 1394 | 3153534 | Reproductive system and breast disorders | 8 | 47421 | 0.38 (0.19-0.76) | 0.38 | 8.14 | -1.29 | -2.25 | 0 | No |
| exclude_target_cirrhosis_indication | 1394 | 3153534 | Ear and labyrinth disorders | 7 | 34761 | 0.45 (0.22-0.95) | 0.46 | 4.60 | -1.03 | -2.06 | 0 | No |
| exclude_target_cirrhosis_indication | 1394 | 3153534 | Surgical and medical procedures | 75 | 155123 | 1.10 (0.87-1.39) | 1.09 | 0.63 | 0.13 | -0.21 | 0 | No |
| exclude_target_cirrhosis_indication | 1394 | 3153534 | Product issues | 4 | 184346 | 0.05 (0.02-0.12) | 0.05 | 78.26 | -4.04 | -5.34 | 0 | No |
| exclude_target_cirrhosis_indication | 1394 | 3153534 | Eye disorders | 32 | 147964 | 0.48 (0.34-0.68) | 0.49 | 17.90 | -1.01 | -1.52 | 0 | No |
| exclude_target_cirrhosis_indication | 1394 | 3153534 | Endocrine disorders | 5 | 26342 | 0.43 (0.18-1.03) | 0.43 | 3.82 | -1.08 | -2.26 | 0 | No |
| exclude_target_cirrhosis_indication | 1394 | 3153534 | Congenital, familial and genetic disorders | 2 | 18671 | 0.24 (0.06-0.97) | 0.24 | 4.77 | -1.63 | -3.29 | 0 | No |
| us_mash_indication_restricted | 1017 | 218 | Investigations | 347 | 27 | 3.66 (2.40-5.59) | 2.75 | 40.17 | 0.17 | -0.01 | 2 | No |
| us_mash_indication_restricted | 1017 | 218 | Nervous system disorders | 172 | 19 | 2.13 (1.30-3.51) | 1.94 | 9.23 | 0.12 | -0.16 | 1 | No |
| us_mash_indication_restricted | 1017 | 218 | Injury, poisoning and procedural complications | 81 | 122 | 0.07 (0.05-0.10) | 0.14 | 301.11 | -1.04 | -1.40 | 0 | No |
| us_mash_indication_restricted | 1017 | 218 | Gastrointestinal disorders | 410 | 56 | 1.95 (1.41-2.71) | 1.57 | 16.35 | 0.10 | -0.07 | 1 | No |
| us_mash_indication_restricted | 1017 | 218 | Skin and subcutaneous tissue disorders | 253 | 15 | 4.48 (2.60-7.72) | 3.62 | 34.22 | 0.19 | -0.03 | 2 | No |
| us_mash_indication_restricted | 1017 | 218 | Hepatobiliary disorders | 217 | 12 | 4.66 (2.55-8.49) | 3.88 | 29.79 | 0.20 | -0.05 | 2 | No |
| us_mash_indication_restricted | 1017 | 218 | General disorders and administration site conditions | 311 | 85 | 0.69 (0.51-0.93) | 0.78 | 5.83 | -0.07 | -0.26 | 0 | No |
| us_mash_indication_restricted | 1017 | 218 | Infections and infestations | 57 | 22 | 0.53 (0.32-0.89) | 0.56 | 6.04 | -0.20 | -0.69 | 0 | No |
| us_mash_indication_restricted | 1017 | 218 | Vascular disorders | 15 | 4 | 0.80 (0.26-2.44) | 0.80 | 0.15 | -0.12 | -1.09 | 0 | No |
| us_mash_indication_restricted | 1017 | 218 | Metabolism and nutrition disorders | 56 | 22 | 0.52 (0.31-0.87) | 0.55 | 6.38 | -0.21 | -0.70 | 0 | No |
| us_mash_indication_restricted | 1017 | 218 | Renal and urinary disorders | 46 | 9 | 1.10 (0.53-2.28) | 1.10 | 0.07 | -0.00 | -0.56 | 0 | No |
| us_mash_indication_restricted | 1017 | 218 | Psychiatric disorders | 64 | 18 | 0.75 (0.43-1.29) | 0.76 | 1.12 | -0.09 | -0.56 | 0 | No |
| us_mash_indication_restricted | 1017 | 218 | Musculoskeletal and connective tissue disorders | 65 | 18 | 0.76 (0.44-1.31) | 0.77 | 1.00 | -0.09 | -0.55 | 0 | No |
| us_mash_indication_restricted | 1017 | 218 | Blood and lymphatic system disorders | 12 | 3 | 0.86 (0.24-3.06) | 0.86 | 0.06 | -0.12 | -1.19 | 0 | No |
| us_mash_indication_restricted | 1017 | 218 | Immune system disorders | 25 | 3 | 1.81 (0.54-6.04) | 1.79 | 0.95 | 0.07 | -0.70 | 0 | No |
| us_mash_indication_restricted | 1017 | 218 | Cardiac disorders | 15 | 8 | 0.39 (0.16-0.94) | 0.40 | 4.73 | -0.37 | -1.30 | 0 | No |
| us_mash_indication_restricted | 1017 | 218 | Neoplasms benign, malignant and unspecified (incl cysts and polyps) | 19 | 5 | 0.81 (0.30-2.20) | 0.81 | 0.17 | -0.11 | -0.97 | 0 | No |
| us_mash_indication_restricted | 1017 | 218 | Reproductive system and breast disorders | 6 | 0 | 2.81 (0.16-50.04) | inf | 1.29 | 0.05 | -1.49 | 0 | No |
| us_mash_indication_restricted | 1017 | 218 | Respiratory, thoracic and mediastinal disorders | 33 | 12 | 0.58 (0.29-1.13) | 0.59 | 2.61 | -0.19 | -0.83 | 0 | No |
| us_mash_indication_restricted | 1017 | 218 | Ear and labyrinth disorders | 6 | 0 | 2.81 (0.16-50.04) | inf | 1.29 | 0.05 | -1.49 | 0 | No |
| us_mash_indication_restricted | 1017 | 218 | Surgical and medical procedures | 63 | 17 | 0.78 (0.45-1.36) | 0.79 | 0.76 | -0.08 | -0.55 | 0 | No |
| us_mash_indication_restricted | 1017 | 218 | Social circumstances | 8 | 2 | 0.86 (0.18-4.06) | 0.86 | 0.04 | -0.16 | -1.45 | 0 | No |
| us_mash_indication_restricted | 1017 | 218 | Product issues | 4 | 3 | 0.28 (0.06-1.27) | 0.29 | 3.08 | -0.60 | -2.24 | 0 | No |
| us_mash_indication_restricted | 1017 | 218 | Eye disorders | 28 | 3 | 2.03 (0.61-6.74) | 2.00 | 1.39 | 0.09 | -0.64 | 0 | No |
| us_mash_indication_restricted | 1017 | 218 | Endocrine disorders | 4 | 2 | 0.43 (0.08-2.34) | 0.43 | 1.02 | -0.43 | -2.12 | 0 | No |
| us_active_comparator_mash | 1017 | 144 | Investigations | 347 | 12 | 5.70 (3.11-10.43) | 4.09 | 39.27 | 0.14 | -0.04 | 2 | No |
| us_active_comparator_mash | 1017 | 144 | Nervous system disorders | 172 | 9 | 3.05 (1.52-6.11) | 2.71 | 10.90 | 0.11 | -0.17 | 2 | No |
| us_active_comparator_mash | 1017 | 144 | Injury, poisoning and procedural complications | 81 | 88 | 0.06 (0.04-0.08) | 0.13 | 286.46 | -0.87 | -1.24 | 0 | No |
| us_active_comparator_mash | 1017 | 144 | Gastrointestinal disorders | 410 | 45 | 1.49 (1.02-2.16) | 1.29 | 4.35 | 0.04 | -0.12 | 1 | No |
| us_active_comparator_mash | 1017 | 144 | Skin and subcutaneous tissue disorders | 253 | 7 | 6.48 (2.99-14.04) | 5.12 | 29.08 | 0.15 | -0.08 | 2 | No |
| us_active_comparator_mash | 1017 | 144 | Hepatobiliary disorders | 217 | 5 | 7.54 (3.05-18.63) | 6.15 | 26.03 | 0.15 | -0.09 | 2 | No |
| us_active_comparator_mash | 1017 | 144 | General disorders and administration site conditions | 311 | 59 | 0.63 (0.44-0.91) | 0.75 | 6.27 | -0.06 | -0.25 | 0 | No |
| us_active_comparator_mash | 1017 | 144 | Infections and infestations | 57 | 12 | 0.65 (0.34-1.25) | 0.67 | 1.68 | -0.10 | -0.60 | 0 | No |
| us_active_comparator_mash | 1017 | 144 | Vascular disorders | 15 | 3 | 0.70 (0.20-2.46) | 0.71 | 0.31 | -0.14 | -1.11 | 0 | No |
| us_active_comparator_mash | 1017 | 144 | Metabolism and nutrition disorders | 56 | 13 | 0.59 (0.31-1.10) | 0.61 | 2.80 | -0.13 | -0.63 | 0 | No |
| us_active_comparator_mash | 1017 | 144 | Renal and urinary disorders | 46 | 1 | 6.77 (0.93-49.51) | 6.51 | 4.76 | 0.13 | -0.45 | 1 | No |
| us_active_comparator_mash | 1017 | 144 | Psychiatric disorders | 64 | 7 | 1.31 (0.59-2.93) | 1.29 | 0.45 | 0.02 | -0.46 | 0 | No |
| us_active_comparator_mash | 1017 | 144 | Musculoskeletal and connective tissue disorders | 65 | 6 | 1.57 (0.67-3.69) | 1.53 | 1.09 | 0.05 | -0.43 | 0 | No |
| us_active_comparator_mash | 1017 | 144 | Blood and lymphatic system disorders | 12 | 3 | 0.56 (0.16-2.01) | 0.57 | 0.81 | -0.20 | -1.28 | 0 | No |
| us_active_comparator_mash | 1017 | 144 | Immune system disorders | 25 | 2 | 1.79 (0.42-7.64) | 1.77 | 0.63 | 0.03 | -0.75 | 0 | No |
| us_active_comparator_mash | 1017 | 144 | Cardiac disorders | 15 | 1 | 2.14 (0.28-16.33) | 2.12 | 0.57 | 0.01 | -0.99 | 0 | No |
| us_active_comparator_mash | 1017 | 144 | Neoplasms benign, malignant and unspecified (incl cysts and polyps) | 19 | 3 | 0.89 (0.26-3.06) | 0.90 | 0.03 | -0.08 | -0.95 | 0 | No |
| us_active_comparator_mash | 1017 | 144 | Reproductive system and breast disorders | 6 | 0 | 1.86 (0.10-33.14) | inf | 0.85 | -0.02 | -1.56 | 0 | No |
| us_active_comparator_mash | 1017 | 144 | Respiratory, thoracic and mediastinal disorders | 33 | 6 | 0.77 (0.32-1.87) | 0.78 | 0.33 | -0.08 | -0.74 | 0 | No |
| us_active_comparator_mash | 1017 | 144 | Ear and labyrinth disorders | 6 | 0 | 1.86 (0.10-33.14) | inf | 0.85 | -0.02 | -1.56 | 0 | No |
| us_active_comparator_mash | 1017 | 144 | Surgical and medical procedures | 63 | 12 | 0.73 (0.38-1.38) | 0.74 | 0.95 | -0.07 | -0.55 | 0 | No |
| us_active_comparator_mash | 1017 | 144 | Social circumstances | 8 | 1 | 1.13 (0.14-9.13) | 1.13 | 0.01 | -0.11 | -1.43 | 0 | No |
| us_active_comparator_mash | 1017 | 144 | Product issues | 4 | 2 | 0.28 (0.05-1.54) | 0.28 | 2.43 | -0.51 | -2.19 | 0 | No |
| us_active_comparator_mash | 1017 | 144 | Eye disorders | 28 | 3 | 1.33 (0.40-4.43) | 1.32 | 0.22 | 0.00 | -0.73 | 0 | No |
| us_active_comparator_mash | 1017 | 144 | Endocrine disorders | 4 | 0 | 1.28 (0.07-23.96) | inf | 0.57 | -0.10 | -1.92 | 0 | No |
| complete_age_sex_only | 63 | 1819373 | Skin and subcutaneous tissue disorders | 23 | 245826 | 3.68 (2.20-6.15) | 2.70 | 28.51 | 1.31 | 0.62 | 3 | Yes |
| complete_age_sex_only | 63 | 1819373 | Nervous system disorders | 20 | 301094 | 2.35 (1.38-3.99) | 1.92 | 10.53 | 0.86 | 0.13 | 2 | No |
| complete_age_sex_only | 63 | 1819373 | Gastrointestinal disorders | 33 | 307287 | 5.41 (3.30-8.87) | 3.10 | 56.53 | 1.53 | 0.91 | 3 | Yes |
| complete_age_sex_only | 63 | 1819373 | Investigations | 17 | 223648 | 2.64 (1.51-4.60) | 2.20 | 12.61 | 1.02 | 0.25 | 3 | Yes |
| complete_age_sex_only | 63 | 1819373 | Hepatobiliary disorders | 10 | 55520 | 5.99 (3.05-11.78) | 5.20 | 35.00 | 1.90 | 0.96 | 3 | Yes |
| complete_age_sex_only | 63 | 1819373 | Infections and infestations | 5 | 261323 | 0.51 (0.21-1.28) | 0.55 | 2.12 | -0.76 | -2.00 | 0 | No |
| complete_age_sex_only | 63 | 1819373 | Surgical and medical procedures | 6 | 90190 | 2.02 (0.87-4.68) | 1.92 | 2.79 | 0.75 | -0.40 | 0 | No |
| complete_age_sex_only | 63 | 1819373 | General disorders and administration site conditions | 20 | 666582 | 0.80 (0.47-1.37) | 0.87 | 0.65 | -0.22 | -0.94 | 0 | No |
| complete_age_sex_only | 63 | 1819373 | Musculoskeletal and connective tissue disorders | 8 | 183188 | 1.30 (0.62-2.73) | 1.26 | 0.48 | 0.27 | -0.75 | 0 | No |
| complete_age_sex_only | 63 | 1819373 | Social circumstances | 2 | 26574 | 2.21 (0.54-9.05) | 2.17 | 1.29 | 0.63 | -1.07 | 0 | No |
| complete_age_sex_only | 63 | 1819373 | Metabolism and nutrition disorders | 4 | 109833 | 1.06 (0.38-2.91) | 1.05 | 0.01 | 0.04 | -1.30 | 0 | No |
| complete_age_sex_only | 63 | 1819373 | Psychiatric disorders | 9 | 168552 | 1.63 (0.81-3.31) | 1.54 | 1.89 | 0.53 | -0.45 | 0 | No |
| complete_age_sex_only | 63 | 1819373 | Congenital, familial and genetic disorders | 2 | 9649 | 6.15 (1.50-25.15) | 5.99 | 8.35 | 1.16 | -0.54 | 0 | No |
| complete_age_sex_only | 63 | 1819373 | Respiratory, thoracic and mediastinal disorders | 4 | 195852 | 0.56 (0.20-1.55) | 0.59 | 1.28 | -0.66 | -2.00 | 0 | No |
| complete_age_sex_only | 63 | 1819373 | Immune system disorders | 2 | 68716 | 0.84 (0.20-3.42) | 0.84 | 0.06 | -0.19 | -1.89 | 0 | No |
| complete_age_sex_only | 63 | 1819373 | Renal and urinary disorders | 2 | 81169 | 0.70 (0.17-2.87) | 0.71 | 0.24 | -0.36 | -2.07 | 0 | No |
| complete_age_sex_only | 63 | 1819373 | Blood and lymphatic system disorders | 1 | 100389 | 0.28 (0.04-1.99) | 0.29 | 1.87 | -1.18 | -3.25 | 0 | No |
| complete_age_sex_only | 63 | 1819373 | Eye disorders | 2 | 89659 | 0.63 (0.15-2.59) | 0.64 | 0.41 | -0.47 | -2.17 | 0 | No |
| complete_age_sex_only | 63 | 1819373 | Vascular disorders | 2 | 106137 | 0.53 (0.13-2.16) | 0.54 | 0.81 | -0.66 | -2.36 | 0 | No |
| complete_age_sex_only | 63 | 1819373 | Ear and labyrinth disorders | 2 | 21771 | 2.71 (0.66-11.07) | 2.65 | 2.08 | 0.76 | -0.94 | 0 | No |
| complete_age_sex_only | 63 | 1819373 | Injury, poisoning and procedural complications | 3 | 579546 | 0.11 (0.03-0.34) | 0.15 | 21.30 | -2.42 | -3.91 | 0 | No |
| complete_age_sex_only | 63 | 1819373 | Neoplasms benign, malignant and unspecified (incl cysts and polyps) | 1 | 86479 | 0.32 (0.04-2.33) | 0.33 | 1.39 | -1.02 | -3.09 | 0 | No |

**Supplementary Table S14. Descriptive time-to-onset summary and exploratory Weibull model output.**

| **Group** | **Valid n** | **Median (IQR), days** | **Weibull shape (95% CI)** | **Interpretation** | **Day-0 handling** |
| --- | --- | --- | --- | --- | --- |
| overall | 206 | 14.0 (1.0-60.0) | 0.57 (0.51-0.64) | early_failure_pattern | 0-day onset shifted to 0.5 days for log-likelihood fitting |
| cluster_hepatocellular_injury | 57 | 28.0 (10.0-60.0) | 0.84 (0.67-1.00) | random_or_indeterminate_pattern | 0-day onset shifted to 0.5 days for log-likelihood fitting |
| cluster_cholestasis_biliary | 24 | 48.5 (14.0-72.0) | 0.85 (0.59-1.10) | random_or_indeterminate_pattern | 0-day onset shifted to 0.5 days for log-likelihood fitting |
| cluster_gastrointestinal_intolerance | 75 | 2.0 (0.0-28.0) | 0.49 (0.41-0.58) | early_failure_pattern | 0-day onset shifted to 0.5 days for log-likelihood fitting |
| cluster_neuropsychiatric | 31 | 2.0 (0.0-9.5) | 0.56 (0.42-0.71) | early_failure_pattern | 0-day onset shifted to 0.5 days for log-likelihood fitting |
| cluster_rash_hypersensitivity | 40 | 6.0 (1.0-51.8) | 0.55 (0.42-0.68) | early_failure_pattern | 0-day onset shifted to 0.5 days for log-likelihood fitting |
| excluded_missing_or_partial_start_dt | 523 | (-) |  |  |  |
| excluded_negative_onset | 194 | (-) |  |  |  |
| excluded_missing_or_partial_event_dt | 506 | (-) |  |  |  |

Note: Only 206 of 1,429 target reports (14.4%) had valid exact onset dates, and onset-available reports differed systematically from reports without valid dates. All onset medians are descriptive selected-subset summaries and do not estimate a causal drug-event latency. Weibull models are exploratory; the cholestasis/biliary fit used only 24 reports and is especially imprecise. Day-0 values were shifted to 0.5 days solely for likelihood fitting and excluded in a separate sensitivity analysis.

**Supplementary Table S15A. Selection analysis for valid exact time-to-onset data.**

Note: Odds ratios compare characteristic prevalence in reports with versus without valid exact dates. Differences in demographic completeness, serious-outcome coding, quarter, and hepatic/biliary prevalence indicate selection into the 206-report subset, not adverse-event associations. Where a characteristic has no variation in either group, its odds ratio is not estimable; no continuity-corrected estimate is presented.

| **Characteristic** | **Valid onset n/N (%)** | **No valid onset n/N (%)** | **Availability OR (95% CI)** | **Note** |
| --- | --- | --- | --- | --- |
| Any serious outcome code | 74/206 (35.9%) | 352/1223 (28.8%) | 1.39 (1.02-1.89) | OR compares characteristic prevalence in reports with versus without valid exact onset dates. |
| Death outcome code | 10/206 (4.9%) | 55/1223 (4.5%) | 1.08 (0.54-2.16) | OR compares characteristic prevalence in reports with versus without valid exact onset dates. |
| Primary-suspect coding | 206/206 (100.0%) | 1201/1223 (98.2%) | 7.73 (0.47-128.00) | OR compares characteristic prevalence in reports with versus without valid exact onset dates. |
| Age available | 33/206 (16.0%) | 130/1223 (10.6%) | 1.60 (1.06-2.43) | OR compares characteristic prevalence in reports with versus without valid exact onset dates. |
| Sex coded as female or male | 34/206 (16.5%) | 42/1223 (3.4%) | 5.56 (3.44-8.98) | OR compares characteristic prevalence in reports with versus without valid exact onset dates. |
| Consumer reporter occupation | 80/206 (38.8%) | 478/1223 (39.1%) | 0.99 (0.73-1.34) | OR compares characteristic prevalence in reports with versus without valid exact onset dates. |
| Report quarter 2024q1 | 0/206 (0.0%) | 0/1223 (0.0%) | Not estimable | No reports in this quarter in either group. |
| Report quarter 2024q2 | 0/206 (0.0%) | 2/1223 (0.2%) | 1.18 (0.06-24.73) | OR compares characteristic prevalence in reports with versus without valid exact onset dates. |
| Report quarter 2024q3 | 23/206 (11.2%) | 86/1223 (7.0%) | 1.66 (1.02-2.70) | OR compares characteristic prevalence in reports with versus without valid exact onset dates. |
| Report quarter 2024q4 | 39/206 (18.9%) | 102/1223 (8.3%) | 2.57 (1.71-3.84) | OR compares characteristic prevalence in reports with versus without valid exact onset dates. |
| Report quarter 2025q1 | 21/206 (10.2%) | 98/1223 (8.0%) | 1.30 (0.79-2.14) | OR compares characteristic prevalence in reports with versus without valid exact onset dates. |
| Report quarter 2025q2 | 25/206 (12.1%) | 126/1223 (10.3%) | 1.20 (0.76-1.90) | OR compares characteristic prevalence in reports with versus without valid exact onset dates. |
| Report quarter 2025q3 | 42/206 (20.4%) | 285/1223 (23.3%) | 0.84 (0.59-1.21) | OR compares characteristic prevalence in reports with versus without valid exact onset dates. |
| Report quarter 2025q4 | 33/206 (16.0%) | 230/1223 (18.8%) | 0.82 (0.55-1.23) | OR compares characteristic prevalence in reports with versus without valid exact onset dates. |
| Report quarter 2026q1 | 23/206 (11.2%) | 294/1223 (24.0%) | 0.40 (0.25-0.62) | OR compares characteristic prevalence in reports with versus without valid exact onset dates. |
| Clinical cluster hepatocellular_injury | 57/206 (27.7%) | 204/1223 (16.7%) | 1.91 (1.36-2.69) | OR compares characteristic prevalence in reports with versus without valid exact onset dates. |
| Clinical cluster cholestasis_biliary | 24/206 (11.7%) | 65/1223 (5.3%) | 2.35 (1.43-3.85) | OR compares characteristic prevalence in reports with versus without valid exact onset dates. |
| Clinical cluster gastrointestinal_intolerance | 75/206 (36.4%) | 411/1223 (33.6%) | 1.13 (0.83-1.54) | OR compares characteristic prevalence in reports with versus without valid exact onset dates. |
| Clinical cluster thyroid_metabolic | 14/206 (6.8%) | 89/1223 (7.3%) | 0.93 (0.52-1.67) | OR compares characteristic prevalence in reports with versus without valid exact onset dates. |
| Clinical cluster muscle_toxicity | 3/206 (1.5%) | 31/1223 (2.5%) | 0.57 (0.17-1.88) | OR compares characteristic prevalence in reports with versus without valid exact onset dates. |
| Clinical cluster neuropsychiatric | 31/206 (15.0%) | 140/1223 (11.4%) | 1.37 (0.90-2.09) | OR compares characteristic prevalence in reports with versus without valid exact onset dates. |
| Clinical cluster cardiovascular | 4/206 (1.9%) | 15/1223 (1.2%) | 1.59 (0.52-4.85) | OR compares characteristic prevalence in reports with versus without valid exact onset dates. |
| Clinical cluster renal | 2/206 (1.0%) | 8/1223 (0.7%) | 1.49 (0.31-7.06) | OR compares characteristic prevalence in reports with versus without valid exact onset dates. |
| Clinical cluster rash_hypersensitivity | 40/206 (19.4%) | 232/1223 (19.0%) | 1.03 (0.71-1.50) | OR compares characteristic prevalence in reports with versus without valid exact onset dates. |

**Supplementary Table S15B. Zero-day sensitivity analyses for descriptive time-to-onset summaries and exploratory Weibull fitting.**

Note: Day-0 intervals were either shifted to 0.5 days for the Weibull likelihood or excluded. Descriptive medians retain the recorded day-0 value unless the row explicitly excludes it. These medians describe only the selected complete-date subset and must not be interpreted as causal latency estimates.

| **Group** | **Day-0 handling** | **Valid n** | **Day-0 n** | **Median (IQR), days** | **Weibull shape (95% CI)** | **Interpretation** |
| --- | --- | --- | --- | --- | --- | --- |
| overall | zero_day_shifted_to_0_5 | 206 | 36 | 14.0 (1.0-60.0) | 0.57 (0.51-0.64) | early_failure_pattern |
| overall | zero_day_excluded | 170 | 36 | 26.0 (5.0-66.0) | 0.72 (0.64-0.80) | early_failure_pattern |
| cluster_hepatocellular_injury | zero_day_shifted_to_0_5 | 57 | 3 | 28.0 (10.0-60.0) | 0.84 (0.67-1.00) | random_or_indeterminate_pattern |
| cluster_hepatocellular_injury | zero_day_excluded | 54 | 3 | 29.0 (13.0-61.5) | 0.93 (0.74-1.11) | random_or_indeterminate_pattern |
| cluster_cholestasis_biliary | zero_day_shifted_to_0_5 | 24 | 2 | 48.5 (14.0-72.0) | 0.85 (0.59-1.10) | random_or_indeterminate_pattern |
| cluster_cholestasis_biliary | zero_day_excluded | 22 | 2 | 58.5 (18.2-74.0) | 1.01 (0.72-1.31) | random_or_indeterminate_pattern |
| cluster_gastrointestinal_intolerance | zero_day_shifted_to_0_5 | 75 | 24 | 2.0 (0.0-28.0) | 0.49 (0.41-0.58) | early_failure_pattern |
| cluster_gastrointestinal_intolerance | zero_day_excluded | 51 | 24 | 7.0 (2.0-45.5) | 0.61 (0.48-0.73) | early_failure_pattern |
| cluster_neuropsychiatric | zero_day_shifted_to_0_5 | 31 | 10 | 2.0 (0.0-9.5) | 0.56 (0.42-0.71) | early_failure_pattern |
| cluster_neuropsychiatric | zero_day_excluded | 21 | 10 | 7.0 (2.0-17.0) | 0.69 (0.47-0.92) | early_failure_pattern |
| cluster_rash_hypersensitivity | zero_day_shifted_to_0_5 | 40 | 8 | 6.0 (1.0-51.8) | 0.55 (0.42-0.68) | early_failure_pattern |
| cluster_rash_hypersensitivity | zero_day_excluded | 32 | 8 | 15.0 (3.0-61.5) | 0.68 (0.50-0.87) | early_failure_pattern |

**Supplementary Table S16. Co-medication patterns and co-medication-by-cluster distributions.**

| **Domain** | **Pattern/subgroup** | **n or subgroup denominator** | **%** | **Event cases** | **Note** |
| --- | --- | --- | --- | --- | --- |
| Pattern | none detected | 1,020 | 71.4 |  |  |
| Pattern | statin | 119 | 8.3 |  |  |
| Pattern | glp1 gip | 71 | 5.0 |  |  |
| Pattern | statin + glp1 gip | 44 | 3.1 |  |  |
| Pattern | statin + hepatotoxic | 24 | 1.7 |  |  |
| Pattern | hepatotoxic | 24 | 1.7 |  |  |
| Pattern | cyp2c8 | 18 | 1.3 |  |  |
| Pattern | statin + cyp2c8 | 17 | 1.2 |  |  |
| Pattern | statin + sglt2 | 16 | 1.1 |  |  |
| Pattern | statin + glp1 gip + sglt2 | 11 | 0.8 |  |  |
| Pattern | sglt2 | 9 | 0.6 |  |  |
| Pattern | statin + glp1 gip + cyp2c8 | 9 | 0.6 |  |  |
| Pattern | statin + glp1 gip + sglt2 + cyp2c8 | 6 | 0.4 |  |  |
| Pattern | statin + hepatotoxic + cyp2c8 | 6 | 0.4 |  |  |
| Pattern | hepatotoxic + cyp2c8 | 5 | 0.3 |  |  |
| Pattern | glp1 gip + sglt2 | 5 | 0.3 |  |  |
| Pattern | statin + glp1 gip + hepatotoxic | 5 | 0.3 |  |  |
| Pattern | statin + sglt2 + hepatotoxic | 4 | 0.3 |  |  |
| Pattern | glp1 gip + sglt2 + hepatotoxic | 3 | 0.2 |  |  |
| Pattern | statin + sglt2 + cyp2c8 | 3 | 0.2 |  |  |
| Pattern | glp1 gip + sglt2 + hepatotoxic + cyp2c8 | 2 | 0.1 |  |  |
| Pattern | statin + glp1 gip + sglt2 + hepatotoxic | 2 | 0.1 |  |  |
| Pattern | glp1 gip + hepatotoxic | 2 | 0.1 |  |  |
| Pattern | glp1 gip + hepatotoxic + cyp2c8 | 1 | 0.1 |  |  |
| Pattern | sglt2 + cyp2c8 | 1 | 0.1 |  |  |
| Pattern | glp1 gip + cyp2c8 | 1 | 0.1 |  |  |
| Pattern | statin + glp1 gip + hepatotoxic + cyp2c8 | 1 | 0.1 |  |  |
| Subgroup by cluster | statin: Hepatocellular injury | 267 | 30.0 | 80 | Co-reporting pattern only; not causal interaction evidence. |
| Subgroup by cluster | statin: Cholestasis/biliary events | 267 | 9.4 | 25 | Co-reporting pattern only; not causal interaction evidence. |
| Subgroup by cluster | statin: Gastrointestinal intolerance | 267 | 36.3 | 97 | Co-reporting pattern only; not causal interaction evidence. |
| Subgroup by cluster | statin: Thyroid/metabolic events | 267 | 9.4 | 25 | Co-reporting pattern only; not causal interaction evidence. |
| Subgroup by cluster | statin: Muscle toxicity | 267 | 7.5 | 20 | Co-reporting pattern only; not causal interaction evidence. |
| Subgroup by cluster | statin: Neuropsychiatric events | 267 | 17.2 | 46 | Co-reporting pattern only; not causal interaction evidence. |
| Subgroup by cluster | statin: Cardiovascular events | 267 | 1.9 | 5 | Co-reporting pattern only; not causal interaction evidence. |
| Subgroup by cluster | statin: Renal events | 267 | 1.1 | 3 | Co-reporting pattern only; not causal interaction evidence. |
| Subgroup by cluster | statin: Rash/hypersensitivity | 267 | 18.0 | 48 | Co-reporting pattern only; not causal interaction evidence. |
| Subgroup by cluster | glp1 gip: Hepatocellular injury | 163 | 27.6 | 45 | Co-reporting pattern only; not causal interaction evidence. |
| Subgroup by cluster | glp1 gip: Cholestasis/biliary events | 163 | 8.0 | 13 | Co-reporting pattern only; not causal interaction evidence. |
| Subgroup by cluster | glp1 gip: Gastrointestinal intolerance | 163 | 42.3 | 69 | Co-reporting pattern only; not causal interaction evidence. |
| Subgroup by cluster | glp1 gip: Thyroid/metabolic events | 163 | 11.7 | 19 | Co-reporting pattern only; not causal interaction evidence. |
| Subgroup by cluster | glp1 gip: Muscle toxicity | 163 | 4.9 | 8 | Co-reporting pattern only; not causal interaction evidence. |
| Subgroup by cluster | glp1 gip: Neuropsychiatric events | 163 | 16.0 | 26 | Co-reporting pattern only; not causal interaction evidence. |
| Subgroup by cluster | glp1 gip: Cardiovascular events | 163 | 0.6 | 1 | Co-reporting pattern only; not causal interaction evidence. |
| Subgroup by cluster | glp1 gip: Renal events | 163 | 0.6 | 1 | Co-reporting pattern only; not causal interaction evidence. |
| Subgroup by cluster | glp1 gip: Rash/hypersensitivity | 163 | 14.1 | 23 | Co-reporting pattern only; not causal interaction evidence. |
| Subgroup by cluster | sglt2: Hepatocellular injury | 62 | 33.9 | 21 | Co-reporting pattern only; not causal interaction evidence. |
| Subgroup by cluster | sglt2: Cholestasis/biliary events | 62 | 8.1 | 5 | Co-reporting pattern only; not causal interaction evidence. |
| Subgroup by cluster | sglt2: Gastrointestinal intolerance | 62 | 33.9 | 21 | Co-reporting pattern only; not causal interaction evidence. |
| Subgroup by cluster | sglt2: Thyroid/metabolic events | 62 | 9.7 | 6 | Co-reporting pattern only; not causal interaction evidence. |
| Subgroup by cluster | sglt2: Muscle toxicity | 62 | 4.8 | 3 | Co-reporting pattern only; not causal interaction evidence. |
| Subgroup by cluster | sglt2: Neuropsychiatric events | 62 | 19.4 | 12 | Co-reporting pattern only; not causal interaction evidence. |
| Subgroup by cluster | sglt2: Cardiovascular events | 62 | 4.8 | 3 | Co-reporting pattern only; not causal interaction evidence. |
| Subgroup by cluster | sglt2: Renal events | 62 | 1.6 | 1 | Co-reporting pattern only; not causal interaction evidence. |
| Subgroup by cluster | sglt2: Rash/hypersensitivity | 62 | 12.9 | 8 | Co-reporting pattern only; not causal interaction evidence. |
| Subgroup by cluster | hepatotoxic: Hepatocellular injury | 79 | 25.3 | 20 | Co-reporting pattern only; not causal interaction evidence. |
| Subgroup by cluster | hepatotoxic: Cholestasis/biliary events | 79 | 7.6 | 6 | Co-reporting pattern only; not causal interaction evidence. |
| Subgroup by cluster | hepatotoxic: Gastrointestinal intolerance | 79 | 41.8 | 33 | Co-reporting pattern only; not causal interaction evidence. |
| Subgroup by cluster | hepatotoxic: Thyroid/metabolic events | 79 | 10.1 | 8 | Co-reporting pattern only; not causal interaction evidence. |
| Subgroup by cluster | hepatotoxic: Muscle toxicity | 79 | 2.5 | 2 | Co-reporting pattern only; not causal interaction evidence. |
| Subgroup by cluster | hepatotoxic: Neuropsychiatric events | 79 | 19.0 | 15 | Co-reporting pattern only; not causal interaction evidence. |
| Subgroup by cluster | hepatotoxic: Cardiovascular events | 79 | 2.5 | 2 | Co-reporting pattern only; not causal interaction evidence. |
| Subgroup by cluster | hepatotoxic: Renal events | 79 | 1.3 | 1 | Co-reporting pattern only; not causal interaction evidence. |
| Subgroup by cluster | hepatotoxic: Rash/hypersensitivity | 79 | 17.7 | 14 | Co-reporting pattern only; not causal interaction evidence. |
| Subgroup by cluster | cyp2c8: Hepatocellular injury | 70 | 24.3 | 17 | Co-reporting pattern only; not causal interaction evidence. |
| Subgroup by cluster | cyp2c8: Cholestasis/biliary events | 70 | 8.6 | 6 | Co-reporting pattern only; not causal interaction evidence. |
| Subgroup by cluster | cyp2c8: Gastrointestinal intolerance | 70 | 41.4 | 29 | Co-reporting pattern only; not causal interaction evidence. |
| Subgroup by cluster | cyp2c8: Thyroid/metabolic events | 70 | 5.7 | 4 | Co-reporting pattern only; not causal interaction evidence. |
| Subgroup by cluster | cyp2c8: Muscle toxicity | 70 | 2.9 | 2 | Co-reporting pattern only; not causal interaction evidence. |
| Subgroup by cluster | cyp2c8: Neuropsychiatric events | 70 | 15.7 | 11 | Co-reporting pattern only; not causal interaction evidence. |
| Subgroup by cluster | cyp2c8: Cardiovascular events | 70 | 2.9 | 2 | Co-reporting pattern only; not causal interaction evidence. |
| Subgroup by cluster | cyp2c8: Renal events | 70 | 0.0 | 0 | Co-reporting pattern only; not causal interaction evidence. |
| Subgroup by cluster | cyp2c8: Rash/hypersensitivity | 70 | 20.0 | 14 | Co-reporting pattern only; not causal interaction evidence. |
| Subgroup by cluster | statin plus glp1 gip: Hepatocellular injury | 78 | 35.9 | 28 | Co-reporting pattern only; not causal interaction evidence. |
| Subgroup by cluster | statin plus glp1 gip: Cholestasis/biliary events | 78 | 6.4 | 5 | Co-reporting pattern only; not causal interaction evidence. |
| Subgroup by cluster | statin plus glp1 gip: Gastrointestinal intolerance | 78 | 35.9 | 28 | Co-reporting pattern only; not causal interaction evidence. |
| Subgroup by cluster | statin plus glp1 gip: Thyroid/metabolic events | 78 | 11.5 | 9 | Co-reporting pattern only; not causal interaction evidence. |
| Subgroup by cluster | statin plus glp1 gip: Muscle toxicity | 78 | 9.0 | 7 | Co-reporting pattern only; not causal interaction evidence. |
| Subgroup by cluster | statin plus glp1 gip: Neuropsychiatric events | 78 | 19.2 | 15 | Co-reporting pattern only; not causal interaction evidence. |
| Subgroup by cluster | statin plus glp1 gip: Cardiovascular events | 78 | 0.0 | 0 | Co-reporting pattern only; not causal interaction evidence. |
| Subgroup by cluster | statin plus glp1 gip: Renal events | 78 | 0.0 | 0 | Co-reporting pattern only; not causal interaction evidence. |
| Subgroup by cluster | statin plus glp1 gip: Rash/hypersensitivity | 78 | 12.8 | 10 | Co-reporting pattern only; not causal interaction evidence. |

Note: Co-medication results indicate co-reporting context only and should not be interpreted as interaction effects. The 70-report CYP2C8-related subgroup is a transparent screening category whose exact drug terms and authoritative source framework are provided in Supplementary Table S2.

**Supplementary Table S17. Deidentified gemfibrozil and clopidogrel co-report summaries.**

Note: All listed gemfibrozil and clopidogrel records were coded as concomitant in FAERS. The current REZDIFFRA label provides the resmetirom-specific interaction context (DailyMed, 2026). These deidentified summaries do not establish concurrent administration, dose, adherence, temporal interaction, or causal attribution and should be interpreted only as a medication-review reminder.

| **Co-medication** | **Case no.** | **Quarter** | **Age/sex** | **Serious outcome** | **Target indication(s)** | **Clinical cluster(s) and PTs** | **Onset days** |
| --- | --- | --- | --- | --- | --- | --- | --- |
| gemfibrozil | 1 | 2025q3 | NA/Unknown | Other serious | Hepatic steatosis; Metabolic dysfunction-associated steatohepatitis | None: Arthritis; Inflammation; Retinal migraine; Thrombosis; Yellow skin | NA |
| gemfibrozil | 2 | 2026q1 | NA/Unknown | None coded | Metabolic dysfunction-associated steatohepatitis | cholestasis_biliary; gastrointestinal_intolerance; neuropsychiatric: Abdominal pain; Headache; Jaundice; Nausea; Ocular icterus; Tongue disorder; Vomiting | 0 |
| gemfibrozil | 3 | 2026q1 | 54.0/Unknown | None coded | Metabolic dysfunction-associated steatohepatitis | muscle_toxicity: Muscle spasms | NA |
| clopidogrel | 1 | 2024q4 | NA/Unknown | None coded | Non-alcoholic steatohepatitis | gastrointestinal_intolerance: Abscess; Diarrhoea; Musculoskeletal stiffness; Pain | NA |
| clopidogrel | 2 | 2024q3 | NA/Unknown | None coded | Non-alcoholic steatohepatitis | hepatocellular_injury; gastrointestinal_intolerance; neuropsychiatric: Accidental overdose; Alanine aminotransferase increased; Alopecia; Aspartate aminotransferase increased; Diarrhoea; Dizziness; Nausea | NA |
| clopidogrel | 3 | 2024q4 | NA/Unknown | Other serious | Non-alcoholic steatohepatitis | None: Blood pressure decreased; Lip swelling | NA |
| clopidogrel | 4 | 2024q4 | NA/Unknown | Other serious | Non-alcoholic steatohepatitis | gastrointestinal_intolerance; neuropsychiatric; cardiovascular; rash_hypersensitivity: Abdominal distension; Abdominal pain; Asthenia; Headache; Hypersensitivity; Hypotension; Lip swelling | NA |
| clopidogrel | 5 | 2024q4 | NA/Unknown | Hospitalization; Other serious | Metabolic dysfunction-associated steatohepatitis | None: Blood potassium increased; Renal impairment; Sepsis | NA |
| clopidogrel | 6 | 2024q4 | NA/Unknown | None coded | Metabolic dysfunction-associated steatohepatitis | hepatocellular_injury; cholestasis_biliary: Alanine aminotransferase increased; Aspartate aminotransferase increased; Blood bilirubin increased | 66 |
| clopidogrel | 7 | 2024q4 | NA/Unknown | None coded | Metabolic dysfunction-associated steatohepatitis | hepatocellular_injury: Alanine aminotransferase increased; Aspartate aminotransferase increased | 11 |
| clopidogrel | 8 | 2025q2 | NA/Unknown | Death; Other serious | Metabolic dysfunction-associated steatohepatitis | None: Haemorrhagic stroke | NA |
| clopidogrel | 9 | 2025q3 | NA/Unknown | None coded | Metabolic dysfunction-associated steatohepatitis | gastrointestinal_intolerance: Abdominal discomfort; Nausea; Vomiting | NA |
| clopidogrel | 10 | 2025q3 | NA/Unknown | Death; Other serious | Metabolic dysfunction-associated steatohepatitis | None: Death | NA |
| clopidogrel | 11 | 2026q1 | NA/Unknown | Hospitalization; Other serious | Metabolic dysfunction-associated steatohepatitis | gastrointestinal_intolerance; cardiovascular; rash_hypersensitivity: Constipation; Dyspnoea; Hunger; Myocardial infarction; Pruritus; Therapy interrupted; Weight increased | 128 |
| clopidogrel | 12 | 2025q3 | NA/Unknown | Other serious | Metabolic dysfunction-associated steatohepatitis | None: Hepatic cirrhosis | NA |
| clopidogrel | 13 | 2025q3 | NA/Unknown | Other serious | Metabolic dysfunction-associated steatohepatitis | hepatocellular_injury; gastrointestinal_intolerance; neuropsychiatric: Abdominal pain; Diarrhoea; Disorientation; Dizziness; Hepatitis; Therapy interrupted | NA |
| clopidogrel | 14 | 2025q3 | NA/Unknown | None coded | Metabolic dysfunction-associated steatohepatitis | rash_hypersensitivity: Pruritus; Rash maculo-papular | NA |
| clopidogrel | 15 | 2025q3 | NA/Unknown | None coded | Metabolic dysfunction-associated steatohepatitis | None: Hepatic lesion | NA |
| clopidogrel | 16 | 2025q4 | NA/Unknown | Other serious | Metabolic dysfunction-associated steatohepatitis | None: Blood pressure increased; Pain in extremity | NA |
| clopidogrel | 17 | 2025q4 | NA/Unknown | None coded | Metabolic dysfunction-associated steatohepatitis | None: Yellow skin | 51 |
| clopidogrel | 18 | 2025q4 | NA/Unknown | None coded | Hepatic steatosis | hepatocellular_injury; cholestasis_biliary; rash_hypersensitivity: Hepatic enzyme increased; Jaundice; Liver disorder; Pruritus | NA |
| clopidogrel | 19 | 2026q1 | NA/Unknown | None coded | Metabolic dysfunction-associated steatohepatitis | None: Spinal stenosis; Vertebral foraminal stenosis | NA |
| clopidogrel | 20 | 2025q4 | NA/Unknown | None coded | Metabolic dysfunction-associated steatohepatitis | gastrointestinal_intolerance; thyroid_metabolic; neuropsychiatric: Hiccups; Hyperglycaemia; Insomnia; Nausea; Therapy interrupted; Vomiting; Weight increased | NA |
| clopidogrel | 21 | 2025q4 | NA/Unknown | None coded | Metabolic dysfunction-associated steatohepatitis | None: Serum ferritin increased | NA |
| clopidogrel | 22 | 2026q1 | NA/Unknown | Hospitalization; Other serious | Metabolic dysfunction-associated steatohepatitis | None: Autoimmune hepatitis; Chronic hepatitis; Dyspnoea; Hypoxia; Intraductal papillary mucinous neoplasm; Oxygen saturation abnormal; Oxygen saturation decreased; Pleural effusion; Primary biliary cholangitis; Weight increased | NA |
| clopidogrel | 23 | 2026q1 | NA/Unknown | Other serious | Metabolic dysfunction-associated steatohepatitis | None: Cerebrovascular accident; Condition aggravated; Metabolic dysfunction-associated steatohepatitis; Therapy interrupted | NA |
| clopidogrel | 24 | 2026q1 | 67.0/Unknown | Other serious | Hepatic fibrosis | hepatocellular_injury: Drug ineffective; Hepatotoxicity; Product use in unapproved indication | NA |
| clopidogrel | 25 | 2026q1 | 66.0/Unknown | Death; Other serious | Metabolic dysfunction-associated steatohepatitis | None: Death | NA |

**Supplementary Table S18. Exploratory serious-outcome analysis.**

| **Model** | **Predictor** | **n** | **Events** | **Odds ratio (95% CI)** | **P value** | **Note** |
| --- | --- | --- | --- | --- | --- | --- |
| descriptive_univariate | Age per 10 years | 63 | 39 |  |  | median serious=59.0; median non-serious=61.0 |
| univariate_2x2 | Male sex | 63 | 39 | 1.16 (0.408-3.294) |  | 2x2: serious+exposed=16, serious+unexposed=23, nonserious+exposed=9, nonserious+unexposed=15 |
| univariate_2x2 | US report | 63 | 39 | Not estimable |  | All 63 reports were US reports; country has no variation. |
| univariate_2x2 | Resmetirom primary suspect | 63 | 39 | 7.40 (1.999-27.427) |  | 2x2: serious+exposed=35, serious+unexposed=4, nonserious+exposed=13, nonserious+unexposed=11 |
| univariate_2x2 | Statin co-report | 63 | 39 | 0.98 (0.280-3.439) |  | 2x2: serious+exposed=8, serious+unexposed=31, nonserious+exposed=5, nonserious+unexposed=19 |
| univariate_2x2 | GLP-1RA/GIP-GLP-1RA co-report | 63 | 39 | 0.90 (0.275-2.949) |  | 2x2: serious+exposed=9, serious+unexposed=30, nonserious+exposed=6, nonserious+unexposed=18 |
| univariate_2x2 | Hepatocellular/biliary event cluster | 63 | 39 | 10.22 (1.234-84.694) |  | 2x2: serious+exposed=12, serious+unexposed=27, nonserious+exposed=1, nonserious+unexposed=23 |
| univariate_2x2 | GI intolerance cluster | 63 | 39 | 0.18 (0.060-0.557) |  | 2x2: serious+exposed=12, serious+unexposed=27, nonserious+exposed=17, nonserious+unexposed=7 |
| univariate_2x2 | Muscle toxicity cluster | 63 | 39 | 0.61 (0.036-10.152) |  | 2x2: serious+exposed=1, serious+unexposed=38, nonserious+exposed=1, nonserious+unexposed=23 |
| multivariable_logistic | Age per 10 years | 63 | 39 | 1.02 (0.634-1.644) | 0.934 | Predictors included: age decade, male; converged=True |
| multivariable_logistic | Male sex | 63 | 39 | 1.17 (0.406-3.355) | 0.774 | Predictors included: age decade, male; converged=True |

*Note: Models are exploratory because complete age/sex data were sparse.*

**Supplementary Table S19. Case-level serious-outcome disproportionality.**

Note: Outcome categories are case-level and not mutually exclusive. These exploratory RORs compare reporting composition, not clinical severity, incidence, hospitalization risk, or mortality risk. The lower serious-outcome ROR (0.35) may reflect enhanced early reporting of mild symptoms and tolerability events and a different target-versus-reference report composition; it must not be interpreted as lower clinical risk.

| **Outcome** | **Target event n** | **Reference event n** | **ROR (95% CI)** | **PRR** | **IC025** | **Interpretation** |
| --- | --- | --- | --- | --- | --- | --- |
| Any serious outcome code | 426 | 1719317 | 0.35 (0.32-0.40) | 0.55 | -1.03 | Exploratory reporting association; not a clinical severity or mortality risk estimate. |
| Death | 65 | 239435 | 0.58 (0.45-0.74) | 0.60 | -1.09 | Exploratory reporting association; not a clinical severity or mortality risk estimate. |
| Life-threatening | 8 | 102373 | 0.17 (0.08-0.34) | 0.17 | -3.36 | Exploratory reporting association; not a clinical severity or mortality risk estimate. |
| Hospitalization | 88 | 634157 | 0.26 (0.21-0.32) | 0.31 | -2.01 | Exploratory reporting association; not a clinical severity or mortality risk estimate. |
| Disability | 7 | 46628 | 0.33 (0.16-0.69) | 0.33 | -2.49 | Exploratory reporting association; not a clinical severity or mortality risk estimate. |
| Congenital anomaly | 0 | 8655 | 0.13 (0.01-2.03) | 0.00 | -5.19 | Exploratory reporting association; not a clinical severity or mortality risk estimate. |
| Required intervention | 4 | 11065 | 0.80 (0.30-2.13) | 0.80 | -1.56 | Exploratory reporting association; not a clinical severity or mortality risk estimate. |
| Other serious | 395 | 1238387 | 0.59 (0.53-0.66) | 0.70 | -0.67 | Exploratory reporting association; not a clinical severity or mortality risk estimate. |
